# Synthesis and Bioactivity of Phthalimide Analogs as Potential Drugs to Treat Schistosomiasis, a Neglected Disease of Poverty

**DOI:** 10.3390/ph13020025

**Published:** 2020-02-03

**Authors:** Snigdha Singh, Nelly El-Sakkary, Danielle E. Skinner, Prem Prakash Sharma, Sabine Ottilie, Yevgeniya Antonova-Koch, Prashant Kumar, Elizabeth Winzeler, Poonam, Conor R. Caffrey, Brijesh Rathi

**Affiliations:** 1 Laboratory for Translational Chemistry and Drug Discovery, Department of Chemistry, Hansraj College, University of Delhi, Delhi 110007, India; snigdhaantil@gmail.com (S.S.); premprakash.cr@gmail.com (P.P.S.); 2 Center for Discovery and Innovation in Parasitic Diseases, Skaggs School of Pharmacy and Pharmaceutical Sciences, University of California San Diego, 9500 Gilman Drive, La Jolla, CA 92093, USA; nelsakkary@health.ucsd.edu (N.E.-S.); deskinner@health.ucsd.edu (D.E.S.); 3 Department of Pediatrics, University of California, San Diego, School of Medicine, La Jolla, CA 92093, USA; sottilie2@gmail.com (S.O.); jantonovakoch@scripps.edu (Y.A.-K.); ewinzeler@ucsd.edu (E.W.); 4 Department of Chemistry, SRM University, Delhi-NCR, Sonepat, Haryana 131029, India; prashanttomar972@gmail.com; 5 Department of Chemistry, Miranda House, University of Delhi, Delhi 110007, India; poonam.chemistry@mirandahouse.ac.in

**Keywords:** phthalimide, benzimidazole, *Schistosoma*, click chemistry, anti-schistosomal activity, tropical disease, drug discovery

## Abstract

The neglected tropical disease, schistosomiasis, is caused by trematode blood flukes of the *Schistosoma* genus and infects approximately 200 million people worldwide. With just one partially effective drug available for disease treatment, new drugs are urgently needed. Herein, a series of 47 phthalimide (Pht) analogues possessing high-value bioactive scaffolds (i.e., benzimidazole and 1,2,3,-triazoles) was synthesized by click-chemistry. Compounds were evaluated for anti-schistosomal activity in culture against somules (post-infective larvae) and adults of *Schistosoma mansoni,* their predicted ADME (absorption, distribution, metabolism, and excretion) properties, and toxicity vs. HepG2 cells. The majority showed favorable parameters for surface area, lipophilicity, bioavailability and Lipinski score. Thirteen compounds were active at 10 µM against both somules and adults (**6d**, **6f**, **6i**–**6l**, **6n**–**6p**, **6s**, **6r’**, **6t’** and **6w**). Against somules, the majority caused degeneracy and/or death after 72 h; whereas against adult parasites, five compounds (**6l**, **6d**, **6f**, **6r’** and **6s**) elicited degeneracy, tegumental (surface) damage and/or death. Strongest potency against both developmental stages was recorded for compounds possessing *n*-butyl or isobutyl as a linker, and a pentafluorophenyl group on triazole. Apart from five compounds for which anti-parasite activity tracked with toxicity to HepG2 cells, there was apparently no toxicity to HepG2 cells (EC_50_ values ≥50 µM). The data overall suggest that phthaloyl-triazole compounds are favorable synthons for additional studies as anti-schistosomals.

## 1. Introduction

Schistosomiasis, also known as bilharzia, is a parasitic disease that infects approximately 200 million people and is caused by trematode flatworms of the genus *Schistosoma* [1]. The hundreds of eggs produced daily by mated pairs elicit a chronic and morbid immuno-inflammatory and fibrotic pathogenesis that can result in pain, malaise and a decreased ability to work [2,3,4,5,6,7]. Among the six principal species infecting humans, namely, *S. mansoni*, *S. japonicum*, S. *haematobium, S. guineensis, S. mekongi* and *S. intercalatum,* the first three are the most important medically [5,7]. Active egg-laying adults can live for years with the longest infections recorded at over 40 years [8,9,10,11]. There are two main forms of schistosomiasis, urinary schistosomiasis [12,13,14] and intestinal schistosomiasis [15,16,17,18]. The former (caused by *S. haematobium*) mainly affects the bladder, kidneys and urogenital system, whereas the latter (due to *S. mansoni* and *S. japonicum)* causes intestinal damage, and hypertension of the abdominal blood vessels, spleen and liver [5,19].

The pyrazylisoquinoline, praziquantel (PZQ), is the only WHO-recommended drug for treatment of schistosomiasis. It is administered orally as a single dose and is active against all schistosome species [20,21]. However, PZQ rarely cures at the 40–60 mg/kg dose offered due to a number of pharmaceutical and pharmacological limitations [21,22]. Also, as the only medication, the possible selection for PZQ-resistant parasites is a concern and such have been generated in the laboratory [23,24]. Therefore, new effective and inexpensive treatments are needed.

The phthalimide (Pht) scaffold has attracted great interest as the basis for the synthesis of various alkaloids and other biologically important pharmacophores [25,26,27,28,29]. Phts are lipophilic and neutral molecules that can easily cross biological membranes, and possess anti-microbial [30] and anti-inflammatory [31] activities. Likewise, *N*-containing heterocyclic moieties such as benzimidazole and triazole, which are found in various natural and synthetic alkaloids, also possess diverse therapeutic applications [32,33]. We have shown that Pht analogues embedded with benzimidazole and flexible triazoles are potent agents against *Plasmodium falciparum (Pf*3D7) and *Pf*W2 malaria strains with 50% inhibitory concentration (IC_50_) values of ~0.7 µM [34]. Also, Pht analogues possessing cyclic amines such as piperazine and piperidine displayed IC_50_ values of <1 µM against the *Pf*7GB malaria strain [35,36]. Encouraged by this, and with a view to discovering new anti-schistosomiasis agents, we employed the Pht synthon to perform the synergistic fusion of highly-valued heterocyclic scaffolds that included the benzimidazole and triazole moieties. Phthaloyl compounds **1**(**a**–**h**) were reacted with *o*-phenylenediamine in the presence of *N*,*N*-Diisopropylethylamine (DIPEA) and 2-(1*H*-Benzotriazole-1-yl)-1,1,3,3-tetramethylaminium tetrafluoroborate (TBTU) and then they were propargylated. Alkyne compounds **5**(**a**–**h**) were treated with different azides by Cu(I) mediated click reaction to provide the desired compounds **6**(**a**–**u’**), which were phenotypically screened against two different stages of the *S. mansoni* parasite; post-infective larvae known as schistosomula (somules) and adult parasites. Also, predicted ADME characteristics were calculated and counter-toxicity screens utilizing HepG2 cells were performed.

## 2. Results and Discussion

### 2.1. Compound Design and Synthesis

We synthesized Pht-scaffold derivatives with known pharmacophore-like substituents *viz.* benzimidazole and triazoles, using simple and cost-effective synthetic routes. The approach was inspired by our recent results that describe a synergistic association of Pht, benzimidazole and triazoles with antiplasmodial activity at submicromolar concentrations [34]. Although of proven value as starting synthons for the construction of bioactive anti-malarial molecules, the potential of Pht analogues against the schistosome parasite is unexplored. Accordingly, we continued to explore and fine-tune the blending of valuable heterocycles, including the introduction of new structural diversity such as a methyl substituent and fluorine on the Pht and on triazole scaffolds, respectively. The introduction of trifluoromethyl on the triazole was anticipated to balance the lipophilicity and improve the metabolic stability in the host. In drug molecules, trifluoromethyl is a popular lipophilic group, as it improves affinity with target enzymes and exerts significant changes on neighboring groups [37].

**Scheme 1:**
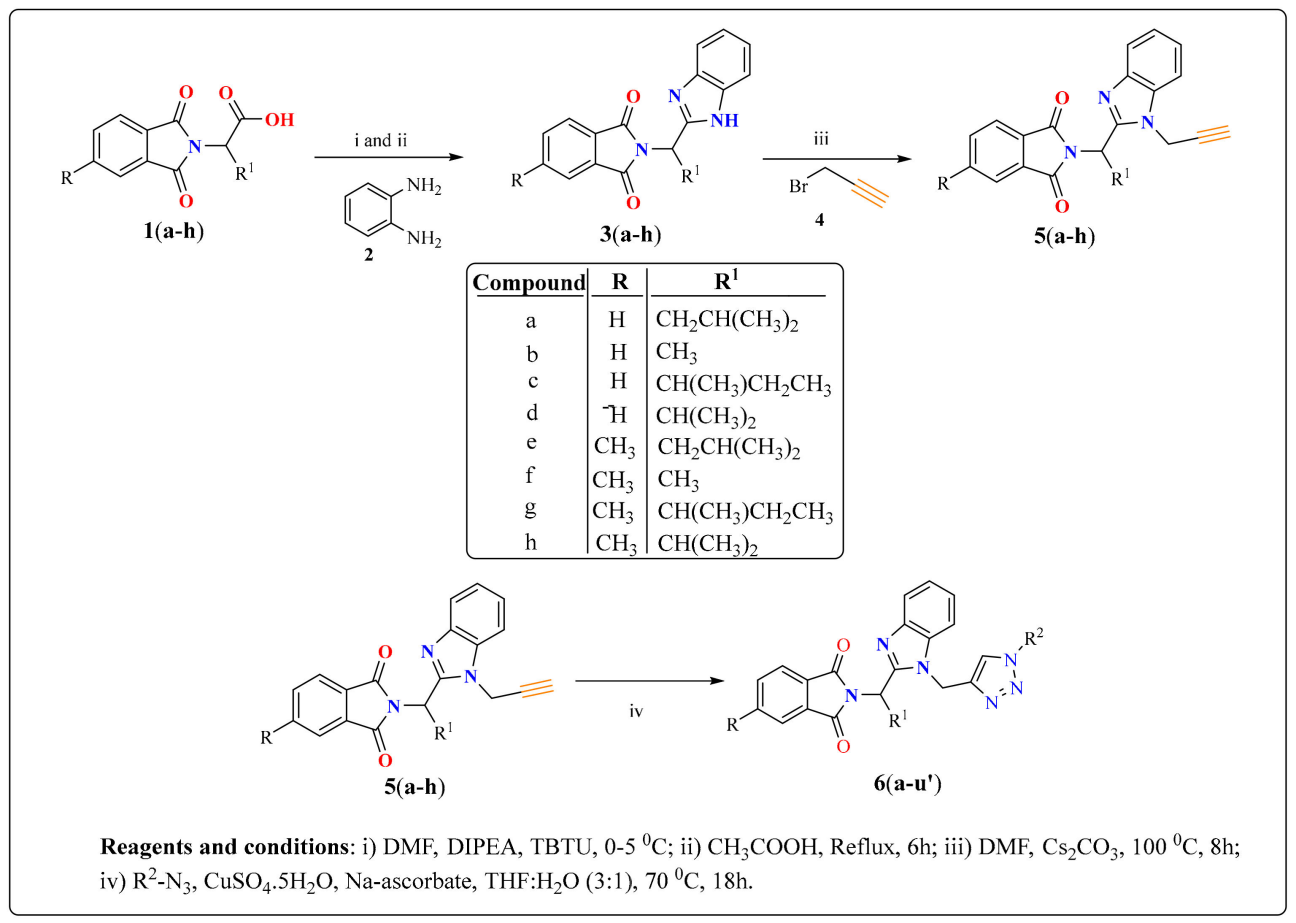
Synthesis of Pht analogues **6**(**a**–**u’**).

Here, we synthesized 47 Pht analogues **6**(**a**–**u’**) in fair to good yields (41 to 82%). The synthetic strategy involved methylated Pht, fluorinated triazoles and various amino acid linkers, as depicted in Scheme 1. The scope and bioactivity of the substituents against the two developmental stages of *S. mansoni* are presented in Table 1. The synthetic routes are simple and several variations on the triazole scaffold were attempted by substituting triazoles with (i) highly electronegative substituents like pentafluorophenyl, trifluoromethylphenyl and 2,4;2,6 difluoro phenyl, and (ii) electron-donating groups such as methyl and methoxyphenyl to try to improve the activity profile and provide insight regarding a structure activity relationship (SAR). Amino acids with aliphatic chains, i.e., alanine, valine, leucine and isoleucine were selected as linkers. The composition of the newly prepared molecules was confirmed by NMR (^1^H &^13^C), and high-resolution mass spectrometry (HR-MS). All the recorded data were in good agreement with the proposed structures.

### 2.2. Predicted ADME Profile of Pht Analogues **6**(**a**–**u’**)

SwissADME predictor was used to define molecular weight, H-bond donors, H-bond acceptors and rotatable bonds, total polar surface area (TPSA), the octanol/water partition coefficients, XLogP3 and MLogP, and the ESOL-LogS (Estimating Aqueous Solubility Directly from Molecular Structure) [38]. All of the listed analogues were also screened for Lipinski rule of 5 compliance using SwissADME [39]. All have upto 10 H-bond acceptors, less than five H-bond donors and less than nine rotatable bonds (Table S1). Compounds **6a**–**c**, **6f**–**k**, **6n**–**r**, **6u**, **6w**–**z**, **6a’**–**n’**, **6s’**–**u’** and **6o’**–**q’** have high predicted GI absorptions over **6l**, **6m**, **6e, 6d**, **6t**, **6r’** and **6s**. Compounds **6c**, **6u**, **6y**, **6z**, **6a’**, **6c’**–**f’**, **6g’**–**i’**, **6k’**–**n’**, **6s’** and **6o’**–**6q’** do not violate Lipinski rules, whereas **6a**, **6b**, **6d**–**t**, **6v**–**x**, **6j’**, **6b’**, **6r’**, **6t’** and **6u’** display one or two violations such as a higher molecular weight (>500 g/mol) and/or MLogP (>4.15). All the analogues have lipophilicity (measured via XlogP3) values within the acceptance range of −0.7 to +6.0 [38]. Also, they display theoretically high intestinal absorption except **6e**, **6d**, **6l**, **6m**, **6t**, **6r’** and **6s**. None of the analogues displayed good predicted blood brain barrier permeability due to their respective TPSA scores being >85 [38]. The ESOL-LogS values for **6c**, **6u**, **6y**, **6z**, **6h’**, **6l’**–**q’**, **6a’**, **6f’** and **6s’** were in the range of-5.85 to −4.63, which indicates moderate solubility in water, whereas **6a**, **6b**, **6d**–**p**, **6r**–**t**, **6v**–**x**, **6j’**, **6r’**, **6t’** and **6u’** exhibited poor solubility parameters. Compounds **6c**, **6h**, **6n**, **6u**, **6v**, **6y**, **6z**, **6a’**, **6c’**–**q’**, and **6s’** showed better bioavailability with a score of 0.55 compared to others with a score of 0.17. Only **6g’** and **6k’** presented good solubility parameters with the corresponding logS values of −3.5 and −3.8, respectively. Both showed a bioavailability score of 0.55.

### 2.3. Bioactivities and Structure-Activity Relationship (SAR)

Functionalized Pht-based molecules were synthesized, and phenotypically screened at 10 µM as a function of time against *S. Mansoni* somules (up to 72 h) and adults (up to 24 h). The resulting phenotypes were classified using a previously described nomenclature that records the many non-exclusive changes (descriptors) that can occur in the parasite in response to chemical insult. Each descriptor is converted into a value from 0 (no effect) to 4 (maximal effect) to allow for comparisons of effects on the parasite between compounds [40,41,42,43]. For consistency, we consider a score of 2 to be the minimum for a compound to be considered active.

Among the 47 compounds tested, 13 were active (i.e., scores of 2 or more) against both somules and adults, namely, **6d**, **6f**, **6i**–**6l**, **6n**–**6p**, **6s**, **6r’**, **6t’** and **6w**. These included compounds with fluorine substituted on the phenyl ring of the triazole with either one, two (**6n**, **6o**, **6p**) or five fluorine atoms (**6d**, **6l**, **6s**, **6r’**). Activity against somules for the majority of these compounds culminated in severe degeneracy or death of at least 50% of the parasites (severity scores of 4) over the 72 h incubation period. For the adult parasite, eight of these compounds (**6i**–**6k**, **6n**–**6p**, **6t’** and **6w**) induced uncoordinated movements and an inability to adhere to the floor of the well via its oral and/or ventral suckers (severity scores of 2) by the last time point, 24 h. The other five compounds (**6l**, **6d**, **6f**, **6r’** and **6s**) elicited more severe responses such as degeneracy, tegumental (surface) damage and/or death (scores ≥3). Interestingly, those analogues with a pentafluorophenyl group on the triazole (e.g., **6d**, **6l**, **6f** and **6s**) with a larger linker group such as *n*-butyl or isobutyl at R^1^ were the most potent, probably due to the high electronegativity and/or increased lipophilicity of the fluorine group. Furthermore, the 4-methyl-substituted (**6d**) and unsubstituted Pht analogues (**6t**), which possess a pentafluorophenyl ring on the triazole moiety and a *n*-butyl group at R^1^, were potently active against adult parasites. 4-methyl-substituted Pht analogues with an unsubstituted benzyl ring on the triazole also exhibited activity against somules (score of 4) and adults (score of 3) but only with *n*-butyl as a linker (e.g., **6f**) and not methyl (e.g., **6m’**).

A smaller number of compounds had stage-specific activities. Thus, for somules at 72 h, **6g**, **6h**, **6x** and **6u’** generated darkened parasites and/or death of at least 50% of the parasites (scores ≥2). Against adult parasites, compounds possessing a fluoro-substituted phenyl ring on the triazole moiety and a *n*-butyl or isobutyl linker (**6a** and **6t**) were potently active (generating a score of 4). In the case of the 4-methyl substituted Pht analogues, a para-fluoro phenyl ring was crucial for the potent activity observed (compare **6a** vs. **6g**), however fluorine present at the ortho position (**6i**) resulted in decreased activity against adults (as core that decreased from 4 to 2).

The screening data indicate the various contributions of R, R^1^and R^2^ to anti-schistosomal activity. As exemplified by the compounds **6d** and **6l**, which possess a *n*-butyl linker and a pentafluorophenyl group on the triazole, varying the R group on the Pht does not seem to alter activity as both compounds were strongly damaging to both stages of the parasite (scores of 4). Interestingly, with the exception of **6r’**, which possesses an isopropyl group, bulky substituents at R^1^ such as isobutyl or *sec*-butyl (**6a**, **6d**, **6f**, **6h**–**l**, **6n**–**p**, **6s** and **6t**), were principally responsible for the anti-parasite activity recorded and those with relatively smaller groups such as a methyl or isopropyl at R^1^ were less active (**6t’** and **6u’**) or completely inactive (**6b’**–**h’** and **6j’**–**n’**).

For all of the tested compounds, the best activity against both developmental stages were recorded with compounds possessing a *n*-butyl linker and a fluorine being substituted on the phenyl ring with either one, two or five fluorine atoms (i.e., **6d**, **6l** and **6f**) with the exception of **6r’** that possesses an isopropyl group as a linker.

For five of the 19 compounds, **6l**, **6d**, **6t**, **6f** (with a *n*-butyl or isobutyl linker) and **6r’**, that were potently active (severity scores ≥3) against somules and/or adults, the counter-toxicity profile with HepG2 cells tracked to some degree with antischistosomal activity: three compounds (**6d**, **6t** and **6r’**) possessed EC_50_ values <10 µM. This might suggest that a shared mechanism of action is being engaged and serves as a caution at this early stage in the SAR exploration. However, it is relevant to note, in the case of the adults at least, that the anti-parasite bioactivity recorded for these particular compounds was already pronounced (severity scores ≥2) at the 5 h time point whereas the HepG2 assay employed a 72 h time point. Thus, the possibility of a yet exploitable selectivity exists. Otherwise, for other 14 of the 19 compounds with anti-parasite activity, there was apparently no toxicity to HepG2 cells with EC_50_ values ≥50 µM (EC_50_ for **6i** = 32 µM). This included **6a**, which registered early and strong activity against adults specifically (severity score of 4 after 5 h).

## 3. Conclusions

Our report employs an economic synthetic route involving the Pht core and cost-effective starting materials and/or intermediates in various organic synthesis reactions [44]. These attributes are important in the context of the highly constrained economics associated with developing drugs for neglected diseases like schistosomiasis [45]. We synthesised via click chemistry a series of Pht compounds containing a unique combination of two different pharmacophores *viz.* benzimidazole and triazoles scaffolds. The compounds presented solid predicted ADME characteristics, and elicited varied and often severe phenotypic changes in one or both developmental stages of *S. mansoni* tested. In particular, **6d**, **6f**, **6l** and **6r’** were potent against both schistosomula and adults. Uniquely, **6a**, which possesses a *n*-butyl linker, was severely damaging to the adult worm (severity score of 4) but inactive against the HepG2 cell lines (EC_50_ > 50 µM). Together, the combination of the fluorinated aryl-triazole and long chain linker was noted to offer the high potency against *S. mansoni*. Future research will include additional medicinal chemistry as well as in vitro and in vivo ADME profiling, and efficacy testing in an animal model of *S. mansoni* infection.

## 4. Experimental

**Chemistry.** The chemicals were purchased from the commercial sources and used without any purification for the experiments. Purity of all the products was initially assayed by thin-layer chromatography (TLC) on alumina-coated plates (Merck). Samples in chloroform (CHCl_3_) were loaded on TLC plates and developed in Ethyl acetate/Petroleum ether (1:1, v/v). When slight impurities were detected by iodine vapour or Ultra Violet light visualization, the compounds were further purified by column chromatography on silica gel columns (100–200 mesh size, CDH). Melting points were determined on Melting point machine M-560 (Buchi). Nuclear Magnetic Resonance (NMR; ^1^H and ^13^C) spectra were recorded in CDCl_3_, DMSO-*d*_6_ medium on a JEOL ECX-400P NMR at 400 MHz and 100 MHz, respectively at University Scientific Instrumentation Center (USIC) and Department of Chemistry, University of Delhi, using TMS as an internal standard. Chemical shifts in ppm (δ-scale) and coupling constants (*J*) in Hz. Splitting patterns are described as singlet (s), doublet (d), doublet of doublet (dd), triplet (t), quartet (q) and multiplet (m). The high-resolution mass spectral (HRMS) data was obtained using a Agilent Technology-6530, Accurate mass, Q-TOF LCMS spectrometer at USIC, University of Delhi. Compounds **1**(**a**–**h**) were prepared following literature procedures [46].

### 4.1. General Procedure for the Synthesis of Compounds ***3**(**a**–**h**)*

In the first step, compounds **1a**–**h** (38 mmol) were dissolved in 250 mL of *N,N*-dimethylformamide (DMF), and DIPEA (45 mmol) was added drop-wise at 0–5 °C. After 10 min, the essential amount of TBTU (45 mmol) was added slowly and the reaction contents were stirred for 30 min at the same temperature. Thereafter, *o*-phenylenediamine (**2**) (38 mmol) was added to the reaction flask and the contents were stirred at 0–5 °C for 6 h. The progress of the reaction was monitored by thin layer chromatography. After completion of the reaction, the reaction mixture was quenched with ice-cold water, and the precipitate obtained was filtered off, washed with excess of cold water, and dissolved in appropriate amount (~300 mL) of ethyl acetate. The organic phase was washed with 1 N HCl followed by saturated solution of NaHCO_3_ and lastly with water. The organic layer was dried over Na_2_SO_4_, filtered and concentrated under reduced pressure that afforded the crude product, which was dissolved in 150 mL of glacial acetic acid and the resulting suspension was refluxed for 6 h. After completion of the reaction, the contents were cooled to room temperature, concentrated under reduced pressure and diluted with ice-cold water. After filtration, the obtained solid was washed with ice cold water followed by saturated NaHCO_3_ solution to obtain the desired products. The products were purified by silica gel column chromatography eluting with 20% mixture of ethyl acetate in hexane, and the final compounds **3**(**a**–**h**) were isolated.

### 4.2. General Procedure for Synthesis of Compounds ***5**(**a**–**h**)*

In a round bottom flask, respective compounds **3**(**a**–**h**) (15 mmol), Cs_2_CO_3_ (45 mmol) were dissolved in appropriate amount of DMF (50 mL) and the contents were heated at 100 °C for 20 min. Subsequently, propargyl bromide (**4**) (22 mmol) was added to the reaction mixture drop wise. This resulted in turbidity in the reaction mixture, which was stirred at 100 °C for next 8 h. After completion of the reaction, the reaction mixture was cooled to attain room temperature and concentrated under reduced pressure to afford residue. Thereafter, ice-cold water was added to the residue and the resulting precipitate was filtered and dried. The obtained solid was purified by silica gel column chromatography eluting with 10% mixture of ethyl acetate in hexane afforded the desired compounds **5**(**a**–**h**).

### 4.3. General Synthetic Procedure for Final Compounds ***6**(**a**–**u’**)*

The synthetic route to prepare new analogues **6(a**–**u’)** is depicted in Scheme 1. In an RB flask, compounds **5**(**a**–**h**) (1.34 mmol) and azides (2.02 mmol) were dissolved in THF:H_2_O (3:1). The required amount of CuSO_4_^.^5H_2_O (0.27 mmol) and sodium ascorbate (0.54 mmol) were added and heated at 70 °C for 18 h. The progress of the reaction was monitored by TLC. After completion, the reaction mixture was concentrated under reduced pressure to give a residue that was quenched with ammonia solution and the precipitate was filtered off. Thus, obtained solid was purified by silica gel column chromatography eluting with appropriate mixture (~25%) of ethyl acetate in petroleum ether to afford the titled compounds **6**(**a**–**u’**). All the novel compounds were identified by careful analysis of their analytical and spectral data, which have been provided in supporting information.

#### 4.3.1. Spectroscopic Data

**2-(1-(1-((1-(4-Fluorophenyl)-1*H*-1,2,3-triazol-4-yl)methyl)-1*H*-benzo[*d*]imidazol-2-yl)-3-methylbutyl)-5-methylisoindoline-1,3-dione (6a):** White solid; m.p. 208–210 °C; Yield: 59%. HRMS *m*/*z* calcd. (M + H)^+^ 523.2180, found 523.2278. ^1^H NMR (400 MHz, CDCl_3_): δ 7.92 (d, *J* = 8.6 Hz, 1H), 7.48 (d, *J* = 7.6 Hz, 1H), 7.34–7.26 (m, 7H), 7.12–7.08 (m, 3H), 5.79 (dd, *J* = 10.9, 4.2 Hz, 1H), 5.51 (s, 2H), 2.87–2.80 (m, 1H), 2.39–2.32 (m, 1H), 2.29 (s, 3H), 1.60 (s, 1H), 1.05 (d, *J* = 6.4 Hz, 3H), 0.99 (d, *J* = 6.6 Hz, 3H). ^13^C NMR (100 MHz, CDCl_3_): δ 167.65, 167.54, 162.30 (d, *J* = 249.5 Hz), 151.25, 144.88 (d, *J* = 119.8 Hz), 142.28, 135.43, 134.64, 132.63, 131.25, 128.33, 123.67 (d, *J* = 6.4 Hz), 122.75, 121.82 (d, *J* = 8.4 Hz), 119.87 (d, *J* = 167.9 Hz), 116.53 (d, *J* = 23.3 Hz), 109.19, 45.19, 39.83, 38.80, 24.84, 23.44, 21.90, 21.72.

**5-Methyl-2-(3-methyl-1-(1-((1-(p-tolyl)-1*H*-1,2,3-triazol-4-yl)methyl)-1*H*-benzo[*d*]imidazol-2-yl)butyl)isoindoline-1,3-dione** (**6b**): White solid; m.p. 160–162 °C; Yield: 78% yield. HRMS *m*/*z* calcd. (M + H)^+^ 519.6089, found 519.2533. ^1^H NMR (400 MHz, CDCl_3_): δ 7.92 (d, *J* = 8.1 Hz, 1H), 7.47 (d, *J* = 7.5 Hz, 1H), 7.35–7.23 (m, 6H), 7.18 (s, 4H), 7.09 (s, 1H), 5.78 (dd, *J* = 10.8, 4.1 Hz, 1H), 5.49 (s, 2H), 2.86–2.79 (m, 1H), 2.39 (m, 1H), 2.36 (s, 3H), 2.24 (s, 3H), 1.59 (d, *J* = 6.1 Hz, 1H), 1.05 (d, *J* = 6.4 Hz, 3H), 0.98 (d, *J* = 6.6 Hz, 3H). ^13^C NMR (100 MHz, CDCl_3_): δ 167.56, 151.21, 145.33, 143.83, 142.21, 138.84, 135.52, 134.63, 133.95, 130.01, 128.18, 123.61, 123.19, 122.68, 120.59, 119.75, 118.75, 109.13, 45.05, 39.94, 38.80, 24.83, 23.47, 21.75, 21.12.

**2-(1-(1-((1H-1,2,3-Triazol-4-yl)methyl)-1*H*-benzo[*d*]imidazol-2-yl)-3-methylbutyl)-5-methylisoindoline-1,3-dione** (**6c**)**:** Light yellow solid; m.p. 150–152 °C; Yield: 58%. HRMS *m*/*z* calcd. (M + H)^+^ 429.4864, found 429.2034. ^1^H NMR (400 MHz, CDCl_3_): δ 7.88 (dd, *J* = 6.3, 1.8 Hz, 1H), 7.57 (d, *J* = 7.6 Hz, 1H), 7.50 (s, 1H), 7.35 (d, *J* = 7.6 Hz, 1H), 7.36–7.24 (m, 5H), 5.79 (dd, *J* = 10.6, 4.7 Hz, 1H), 5.49 (s, 2H), 2.94–2.77 (m, 1H), 2.39 (s, 3H), 2.32–2.24 (m, 1H), 1.58 (s, 1H), 1.00 (d, *J* = 6.5 Hz, 3H), 0.93 (d, *J* = 6.7 Hz, 3H). ^13^C NMR (100 MHz, CDCl_3_): δ 167.85, 167.77, 151.23, 145.55, 141.87, 135.18, 134.77, 131.61, 128.66, 123.97, 123.58, 123.36, 122.77, 120.40, 109.69, 44.91, 39.40, 38.80, 24.87, 23.16, 21.89, 21.66.

**5-Methyl-2-(3-methyl-1-(1-((1-(perfluorophenyl)-1*H*-1,2,3-triazol-4-yl)methyl)-1*H*-benzo[*d*]imidazol-2-yl)butyl)isoindoline-1,3-dione (6d):** Light brown solid; m.p. 128–130 °C; Yield: 69%. HRMS *m/z* calcd. (M + H)^+^ 595.5346, found 595.1882. ^1^H NMR (400 MHz, CDCl_3_): δ 7.86 (d, *J* = 7.2 Hz, 1H), 7.61 (d, *J* = 7.5 Hz, 1H), 7.53 (s, 1H), 7.44 (d, *J* = 7.4 Hz, 1H), 7.38 (s, 1H), 7.30–7.25 (m, 4H), 5.79 (dd, *J* = 10.3, 4.2 Hz, 1H), 5.56 (d, *J* = 6.3 Hz, 2H), 2.91–2.80 (m, 1H), 2.47 (d, *J* = 17.6 Hz, 3H), 2.34–2.24 (m, 1H), 1.58 (brs, 1H), 1.01 (d, *J* = 6.3 Hz, 3H), 0.96 (d, *J* = 6.1 Hz, 3H). ^13^C NMR (100 MHz, CDCl_3_): δ 167.90, 167.80, 151.16, 144.73 (d, *J* = 210.4 Hz), 142.31, 135.10, 134.85, 131.70, 128.72, 124.10 (d, *J* = 26.3 Hz), 123.47 (d, *J* = 20.0 Hz), 121.65 (d, *J* = 211.1 Hz), 109.41, 45.23, 39.19, 38.86, 24.94, 23.23, 21.97, 21.68.

**5-Methyl-2-(3-methyl-1-(1-((1-(4-(trifluoromethyl)phenyl)-1*H*-1,2,3-triazol-4-yl)methyl)-1*H*-benzo[*d*]imidazol-2-yl)butyl)isoindoline-1,3-dione (6e):** White solid; m.p. 218–220 °C. Yield: 71%. HRMS *m/z* calcd. (M + H)^+^ 573.5803, found 573.2225. ^1^H NMR (400 MHz, CDCl_3_): δ 7.90 (d, *J* = 8.1 Hz, 1H), 7.66 (d, *J* = 8.4 Hz, 2H), 7.46 (dd, *J* = 17.9, 8.0 Hz, 3H), 7.27–7.21 (m, 7H), 5.77 (dd, *J* = 10.9, 4.1 Hz, 1H), 5.50 (s, 2H), 2.88–2.77 (m, 1H), 2.38–2.29 (m, 1H), 2.20 (s, 3H), 1.57 (brs, 1H), 1.03 (d, *J* = 6.6 Hz, 3H), 0.97 (d, *J* = 6.4 Hz, 3H). ^13^C NMR (100 MHz, CDCl_3_): δ 167.62, 167.48, 151.26, 145.50, 144.76, 142.35, 138.67, 135.43, 134.62, 131.23, 130.88, 130.55, 128.28, 126.91, 126.88, 124.83, 123.69, 123.65, 123.28, 122.80, 122.12, 120.80, 119.81, 118.82, 109.10, 45.21, 39.81, 38.81, 24.84, 23.44, 21.79, 21.71.

**2-(1-(1-((1-Benzyl-1*H*-1,2,3-triazol-4-yl)methyl)-1*H*-benzo[*d*]imidazol-2-yl)-3-methylbutyl)-5-methylisoindoline-1,3-dione** (**6f**)**:** Yellow solid; m.p. 142–144 °C; Yield: 41%. HRMS *m/z* calcd. (M + H)^+^ 519.6089, found 519.2505. ^1^H NMR (400 MHz, CDCl_3_): δ 7.85 (d, *J* = 7.7 Hz, 1H), 7.57 (d, *J* = 7.6 Hz, 1H), 7.52 (s, 1H), 7.45 (d, *J* = 7.6 Hz, 1H), 7.30–7.28 (m, 4H), 7.27–7.21 (m, 4H), 7.09–7.06 (m, 1H), 7.00 (s, 2H), 5.76 (dd, *J* = 10.7, 4.5 Hz, 1H), 5.51 (d, *J* = 8.4 Hz, 1H), 5.44 (s, 1H), 5.28–5.09 (m, 2H), 2.87–2.80 (m, 1H), 2.47 (s, 3H), 2.26–2.21 (m, 1H), 1.82 (brs, 1H), 0.95 (dd, *J* = 16.5, 6.6 Hz, 6H). ^13^C NMR (100 MHz, CDCl_3_): δ 167.83, 167.73, 151.19, 145.51, 142.30, 135.25, 134.79, 134.18, 131.79, 129.13, 128.83, 127.95, 123.94, 123.40, 123.30, 122.53, 121.39, 120.57, 109.59, 54.04, 45.21, 39.61, 38.90, 24.93, 23.32, 22.15, 21.71.

**5-Methyl-2-(3-methyl-1-(1-((1-phenyl-1*H*-1,2,3-triazol-4-yl)methyl)-1*H*-benzo[*d*]imidazol-2-yl)butyl)isoindoline-1,3-dione** (**6g**)**:** Yellow solid; m.p. 67–69 °C; Yield: 57%. HRMS *m/z* calcd. (M + H)^+^ 505.5823, found 505.2346. ^1^H NMR (400 MHz, CDCl_3_): δ 7.93 (d, *J* = 7.6 Hz, 1H), 7.47 (d, *J* = 7.4 Hz, 1H), 7.43–7.37 (m, 3H), 7.35–7.25 (m, 8H), 7.12 (s, 1H), 5.79 (dd, *J* = 10.9, 4.2 Hz, 1H), 5.51 (s, 2H), 2.89–2.78 (m, 1H), 2.41–2.31 (m, 1H), 2.22 (s, 3H), 1.68 (brs, 1H), 1.05 (d, *J* = 6.4 Hz, 3H), 0.99 (d, *J* = 6.5 Hz, 3H). ^13^C NMR (100 MHz, CDCl_3_): δ 167.68, 167.56, 156.41, 151.27, 145.56, 144.03, 142.01, 136.25, 135.38, 134.68, 131.13, 129.63, 129.60, 128.83, 128.21, 123.81, 123.76, 123.35, 122.94, 120.61, 120.21, 119.84, 118.82, 115.60, 109.25, 45.13, 39.98, 38.78, 24.82, 23.39, 21.87, 21.73.

**2-(1-(1-((1-(4-Methoxyphenyl)-1*H*-1,2,3-triazol-4-yl)methyl)-1*H*-benzo[*d*]imidazol-2-yl)-3-methylbutyl)-5-methylisoindoline-1,3-dione (6h):** Yellow solid; m.p. 101–103 °C; Yield: 82%. HRMS *m/z* calcd. (M + H)^+^ 535.6083, found 535.2448. ^1^H NMR (400 MHz, CDCl_3_): δ 7.92 (d, *J* = 7.9 Hz, 1H), 7.48 (d, *J* = 7.5 Hz, 1H), 7.31–7.21 (m, 8H), 7.05 (s, 1H), 6.88 (d, *J* = 9.0 Hz, 1H), 5.79 (dd, *J* = 10.9, 4.1 Hz, 1H), 5.49 (s, 2H), 3.82 (s, 3H), 2.88–2.78 (m, 1H), 2.40–2.31 (m, 1H), 2.27 (s, 3H), 1.60 (brs, 1H), 1.05 (d, *J* = 6.4 Hz, 3H), 0.98 (d, *J* = 6.6 Hz, 3H). ^13^C NMR (100 MHz, CDCl_3_): δ 167.68, 167.57, 159.87, 151.30, 145.45, 143.91, 142.45, 135.49, 134.64, 131.22, 129.81, 128.31, 123.75, 123.63, 123.32, 122.72, 121.47, 120.72, 118.89, 114.52, 109.22, 55.71, 45.16, 39.98, 38.79, 24.83, 23.49, 21.95, 21.73.

**2-(1-(1-((1-(2-Fluorophenyl)-1*H*-1,2,3-triazol-4-yl)methyl)-1*H*-benzo[*d*]imidazol-2-yl)-3-methylbutyl)-5-methylisoindoline-1,3-dione (6i):** Yellow solid; m.p. 138–140 °C; Yield: 67%. HRMS *m/z* calcd. (M + H)^+^ 523.5728, found 523.2246. ^1^H NMR (400 MHz, CDCl_3_): δ 7.88 (d, *J* = 8.3 Hz, 1H), 7.56–7.52 (m, 2H), 7.44–7.42 (m, 2H), 7.35–7.19 (m, 8H), 5.81 (dd, *J* = 10.8, 4.4 Hz, 1H), 5.52 (s, 2H), 2.91–2.78 (m, 1H), 2.33 (s, 3H), 1.64–1.53 (m, 1H), 1.23 (s, 1H), 1.03 (d, *J* = 6.4 Hz, 3H), 0.97 (d, *J* = 6.7 Hz, 3H). ^13^C NMR (100 MHz, CDCl_3_): δ 167.64 (d, *J* = 6.9 Hz), 151.67, 151.23, 145.43, 143.62, 142.32, 135.40, 134.64, 131.59, 130.30 (d, *J* = 7.7 Hz), 128.66, 125.04, 124.66, 124.40, 123.85, 123.47 (d, *J* = 17.8 Hz), 122.66, 122.37, 120.69, 117.04 (d, *J* = 20.1 Hz), 109.38, 45.17, 39.62, 38.81, 24.91, 23.39, 21.96, 21.74.

**2-(1-(1-((1-(2,4-Difluorophenyl)-1*H*-1,2,3-triazol-4-yl)methyl)-1*H*-benzo[*d*]imidazol-2-yl)-3-methylbutyl)-5-methylisoindoline-1,3-dione (6j):** Yellow solid; m.p. 72–74 °C; Yield: 50%. ^1^H NMR (400 MHz, CDCl_3_): δ 7.89 (s, 1H), 7.56–7.37 (m, 4H), 7.38 (d, *J* = 7.5 Hz, 2H), 7.26 (d, *J* = 11.9 Hz, 2H), 6.96 (d, *J* = 7.8 Hz, 2H), 5.84 (d, *J* = 4.8 Hz, 1H), 5.56 (s, 2H), 2.91 (brs, 1H), 2.37 (s, 3H), 1.85 (brs, 1H), 1.58 (s, 1H), 0.99 (dd, *J* = 22.8, 6.1 Hz, 6H). ^13^C NMR (100 MHz, CDCl_3_): δ 167.63, 162.44 (d, *J* = 264.6 Hz), 152.22, 151.22, 145.47, 143.01 (d, *J* = 142.5 Hz), 135.28, 134.68, 131.65, 128.72, 125.80 (d, *J* = 9.6 Hz), 123.91, 123.61, 123.41, 122.73, 122.39, 121.44, 120.74, 112.48 (d, *J* = 20.4 Hz), 109.40, 105.65, 105.40, 105.15, 45.17, 39.54, 38.85, 24.94, 23.37, 22.03, 21.74.

**2-(1-(1-((1-(2,6-Difluorophenyl)-1*H*-1,2,3-triazol-4-yl)methyl)-1*H*-benzo[*d*]imidazol-2-yl)-3-methylbutyl)-5-methylisoindoline-1,3-dione (6k):** White solid; m.p. 71–73 °C; Yield: 43%. HRMS *m/z* calcd. (M + H)^+^ 541.5632, found 541.2173. ^1^H NMR (400 MHz, CDCl_3_): δ 7.86 (d, *J* = 4.6 Hz, 1H), 7.60 (d, *J*= 7.4 Hz, 1H), 7.51 (s, 1H), 7.41 (d, *J* = 7.5 Hz, 2H), 7.37–7.31 (m, 2H), 7.25 (d, *J* = 3.9 Hz, 3H), 7.01 (t, *J* = 8.6 Hz, 2H), 5.82 (dd, *J* = 10.5, 4.6 Hz, 1H), 5.56 (s, 2H), 2.94–2.80 (m, 1H), 2.41 (s, 3H), 2.35–2.26 (m, 1H), 1.63–1.52 (m, 1H), 0.98 (dd, *J* = 22.6, 6.5 Hz, 6H). ^13^C NMR (100 MHz, CDCl_3_): δ 167.84, 156.51 (d, *J* = 257.0 Hz), 151.25, 145.67, 142.96, 142.20, 134.87, 131.59 (d, *J* = 14.6 Hz), 128.72, 124.21, 124.07, 123.69, 123.47, 122.79, 120.64, 112.49 (d, *J* = 19.5 Hz), 109.60, 45.12, 39.48, 38.91, 24.97, 23.27, 22.08, 21.74.

**2-(3-Methyl-1-(1-((1-(perfluorophenyl)-1*H*-1,2,3-triazol-4-yl)methyl)-1*H*-benzo[*d*]imidazol-2-yl)butyl)isoindoline-1,3-dione** (**6l**)**:** Light brown solid; m.p. 100–102 °C; Yield: 65%. HRMS *m/z* calcd. (M + H)^+^ 581.5081, found 581.1750. ^1^H NMR (400 MHz, CDCl_3_): δ 7.92–7.85 (m, 1H), 7.78–7.74 (m, 2H), 7.70–7.76 (m, 2H), 7.43 (s, 1H), 7.35–7.31 (m, 1H), 7.25–7.29 (m, 2H), 5.84 (dd, *J* = 10.6, 4.7 Hz, 1H), 5.65–5.53 (m, 2H), 2.93–2.81 (m, 1H), 2.39–2.25 (m, 1H), 1.61 (s, 1H), 1.03 (d, *J* = 6.6 Hz, 3H), 0.98 (d, *J* = 6.5 Hz, 3H). ^13^C NMR (100 MHz, CDCl_3_): δ 167.76, 151.03, 143.68, 142.37, 135.10, 134.32, 131.39, 124.19, 123.66, 123.50, 122.79, 120.77, 109.34, 45.33, 39.22, 38.90, 24.96, 23.24, 21.71.

**2-(3-Methyl-1-(1-((1-(4-(trifluoromethyl)phenyl)-1*H*-1,2,3-triazol-4-yl)methyl)-1*H*-benzo[*d*]imidazol-2-yl)butyl)isoindoline-1,3-dione (6m):** White solid; m.p. 216–218 °C; Yield: 62%. HRMS *m/z* calcd. (M + H)^+^ 559.5537, found 559.2070. ^1^H NMR (400 MHz, CDCl_3_): δ 7.91 (d, *J* = 8.5 Hz, 1H), 7.66 (d, *J* = 8.4 Hz, 2H), 7.58–7.54 (m, *J* = 5.3, 2.9 Hz, 2H), 7.48 (d, *J* = 7.3 Hz, 3H), 7.32–7.22 (m, 5H), 5.80 (dd, *J* = 10.7, 4.4 Hz, 1H), 5.51 (s, 2H), 2.89–2.77 (m, 1H), 2.39–2.29 (m, 1H), 1.60 (s, 1H), 1.04 (d, *J* = 6.4 Hz, 3H), 0.98 (d, *J* = 6.5 Hz, 3H). ^13^C NMR (100 MHz, CDCl_3_): δ 167.52, 151.13, 144.66, 142.38, 138.68, 135.38, 134.10, 130.99, 130.66, 126.97, 126.93, 124.83, 123.71, 123.26, 122.82, 122.12, 120.82, 120.02, 119.05, 109.14, 45.37, 39.74, 38.88, 24.88, 23.42, 21.70.

**2-(1-(1-((1-(2-Fluorophenyl)-1*H*-1,2,3-triazol-4-yl)methyl)-1*H*-benzo[*d*]imidazol-2-yl)-3-methylbutyl)isoindoline-1,3-dione** (**6n**)**:** Yellow solid; m.p. 107–109 °C; Yield: 78%. HRMS *m/z* calcd. (M + H)^+^ 509.5462, found 509.2106. ^1^H NMR (400 MHz, CDCl_3_): δ 7.85 (d, *J* = 8.0 Hz, 1H), 7.65 (dd, *J* = 5.1, 3.0 Hz, 2H), 7.60–7.46 (m, 4H), 7.38–7.10 (m, 7H), 5.84 (dd, *J* = 10.7, 4.4 Hz, 1H), 5.51 (s, 2H), 2.94–2.82 (m, 1H), 2.41–2.27 (m, 1H), 1.65–1.60 (brs, 1H), 1.03 (d, *J* = 6.5 Hz, 3H), 0.96 (d, *J* = 6.6 Hz, 3H). ^13^C NMR (100 MHz, CDCl_3_): δ 167.80, 152.94 (d, *J* = 251.5 Hz), 143.46, 142.29, 135.33, 134.12, 131.25, 130.40 (d, *J* = 7.7 Hz), 125.12 (d, *J* = 3.0 Hz), 124.67, 124.50, 123.55, 123.38, 122.65, 122.47 (d, *J* = 8.4 Hz), 120.64, 117.01 (d, *J* = 19.8 Hz), 109.43, 45.21, 39.50, 38.81, 24.90, 23.39, 21.73.

**2-(1-(1-((1-(2,4-Difluorophenyl)-1*H*-1,2,3-triazol-4-yl)methyl)-1*H*-benzo[*d*]imidazol-2-yl)-3-methylbutyl)isoindoline-1,3-dione (6o):** Yellow solid; m.p. 126–128 °C; Yield: 68%. HRMS *m/z* calcd. (M + H)^+^ 527.5367, found 527.2007. ^1^H NMR (400 MHz, CDCl_3_): δ 7.89 (d, *J* = 7.0 Hz, 1H), 7.71–7.67 (m, 2H), 7.65–7.58 (m, 2H), 7.55–7.44 (m, 2H), 7.34 (d, *J* = 7.1 Hz, 1H), 7.30–7.27 (m, 3H), 6.98–6.93 (m, 2H), 5.85 (dd, *J* = 10.4, 4.0 Hz, 1H), 5.55 (s, 2H), 2.95–2.83 (m, 1H), 2.40–2.27 (m, 1H), 1.69 (brs, 2H), 1.05 (d, *J* = 6.3 Hz, 3H), 0.99 (d, *J* = 6.5 Hz, 3H). ^13^C NMR (100 MHz, CDCl_3_): δ 167.59, 151.09, 142.96 (d, *J* = 132.2 Hz), 135.24, 134.13, 131.34, 125.90 (d, *J* = 9.9 Hz), 123.62, 123.43, 122.74, 122.44 (d, *J* = 7.4 Hz), 120.73, 112.53 (d, *J* = 19.2 Hz), 109.39, 105.65, 105.41, 105.39, 105.15, 45.25, 39.48, 38.86, 24.94, 23.35, 21.73.

**2-(1-(1-((1-(2,6-Difluorophenyl)-1*H*-1,2,3-triazol-4-yl)methyl)-1*H*-benzo[*d*]imidazol-2-yl)-3-methylbutyl)isoindoline-1,3-dione (6p):** Yellow solid; m.p. 198–200 °C; Yield: 65%. HRMS *m/z* calcd. (M + H)^+^ 527.5367, found 527.2014. ^1^H NMR (400 MHz, CDCl_3_): δ 7.83 (d, *J* = 4.4 Hz, 1H), 7.71 (s, 2H), 7.61 (s, 2H), 7.41–7.28 (m, 3H), 7.23 (d, *J* = 2.9 Hz, 2H), 6.98 (t, *J* = 8.2 Hz, 2H), 5.83 (dd, *J* = 9.8, 3.8 Hz, 1H), 5.54 (s, 2H), 2.93–2.77 (m, 1H), 2.34–2.23 (m, 1H), 1.58 (brs, 1H), 1.00 (d, *J* = 6.0 Hz, 3H), 0.95 (d, *J* = 6.2 Hz, 3H). ^13^C NMR (100 MHz, CDCl_3_): δ 167.79, 156.50 (d, *J* = 256.9 Hz), 151.13, 142.67 (d, *J* = 59.1 Hz), 135.24, 134.26, 131.65, 131.55, 131.45, 131.37, 124.24, 123.54, 123.48, 122.63, 120.66, 114.72, 112.51 (d, *J* = 22.8 Hz), 109.51, 45.30, 39.39, 38.89, 24.96, 23.30, 21.73.

**2-(1-(1-((1-(2-Fluorophenyl)-1*H*-1,2,3-triazol-4-yl)methyl)-1*H*-benzo[*d*]imidazol-2-yl)-2-methylbutyl)-5-methylisoindoline-1,3-dione (6q):** Light green solid; m.p. 114–116 °C; Yield: 60%. HRMS *m/z* calcd. (M + H)^+^ 523.5725, found 523.2258. ^1^H NMR (400 MHz, CDCl_3_): δ 7.86 (s, 1H), 7.66 (t, *J* = 29.0 Hz, 3H), 7.50 (s, 2H), 7.38 (s, 2H), 7.23 (d, *J* = 13.0 Hz, 4H), 5.76 (s, 1H), 5.57 (s, 1H), 5.44 (s, 1H), 3.57 (brs, 1H), 2.37 (s, 3H), 1.52 (brs, 1H), 1.11 (s, 1H), 0.94 (d, *J* = 22.6 Hz, 6H). ^13^C NMR (100 MHz, CDCl_3_): δ 167.78, 154.39, 145.70, 134.90, 131.66, 130.51, 128.67, 125.29, 124.57, 124.11, 123.86, 123.58, 122.91, 120.51, 117.20, 117.01, 27.01, 25.50, 22.08, 16.92, 15.54, 11.24, 10.58.

**2-(1-(1-((1-(2,4-Difluorophenyl)-1*H*-1,2,3-triazol-4-yl)methyl)-1*H*-benzo[*d*]imidazol-2-yl)-2-methylbutyl)-5-methylisoindoline-1,3-dione (6r):** Yellow solid; m.p. 89–91 °C; Yield: 50%. HRMS *m/z* calcd. (M + H)^+^ 541.5632, found 541.2164. ^1^H NMR (400 MHz, CDCl_3_): δ 7.84 (d, *J* = 4.8 Hz, 1H), 7.58–7.56 (m, 3H), 7.47 (s, 1H), 7.37 (d, *J* = 6.5 Hz, 2H), 7.25–7.18 (m, 2H), 6.93 (d, *J* = 7.6 Hz, 2H), 5.73–5.50 (m, 2H), 5.38 (d, *J* = 10.8 Hz, 1H), 3.41 (brs, 1H), 2.37 (s, 3H), 1.53 (d, *J* = 42.3 Hz, 1H), 1.15–1.10 (m, 1H), 1.00–0.85 (m, 6H). ^13^C NMR (100 MHz, CDCl_3_): δ 167.79, 150.43 (d, *J* = 16.4 Hz), 145.52, 143.83, 143.78, 142.53, 134.73, 130.18 (d, *J* = 297.2 Hz), 126.01, 125.91, 123.96, 123.43, 122.62, 120.68, 112.54 (d, *J* = 24.4 Hz), 109.77, 105.61, 105.36, 105.10, 51.81, 51.51, 39.19, 34.45, 33.94, 27.10, 25.45, 22.00, 17.02, 15.41, 11.13, 10.66.

**5-Methyl-2-(2-methyl-1-(1-((1-(perfluorophenyl)-1*H*-1,2,3-triazol-4-yl)methyl)-1H-benzo[d]imidazol-2-yl)butyl)isoindoline-1,3-dione (6s):** White solid; m.p. 196–198 °C; Yield: 64%. ^1^H NMR (400 MHz, CDCl_3_): δ 7.86–7.83 (m, 1H), 7.63 (d, *J* = 7.7 Hz, 1H), 7.55–7.52 (m, 2H), 7.45 (d, *J* = 7.6 Hz, 1H), 7.38 (dd, *J* = 6.1, 3.0 Hz, 1H), 7.24 (dd, *J* = 6.0, 3.2 Hz, 3H), 5.82–5.68 (m, 1H), 5.60 (dd, *J* = 16.8, 7.9 Hz, 1H), 5.35 (d, *J* = 11.0 Hz, 1H), 3.52–3.30 (m, 1H), 2.44 (s, 3H), 1.54–1.47 (m, 1H), 0.96–0.81 (m, 7H).^13^C NMR (100 MHz, CDCl_3_): δ 167.98, 167.90, 150.58, 150.38, 145.77, 143.99, 143.92, 142.57, 135.16, 134.87, 134.70, 134.57, 131.72, 128.73, 124.39, 124.09, 123.56, 123.49, 122.76, 120.78, 109.72, 77.48, 77.16, 76.84, 51.88, 51.66, 38.98, 34.42, 33.92, 27.02, 25.49, 22.03, 16.87, 15.45, 11.04, 10.59.

**2-(2-Methyl-1-(1-((1-(perfluorophenyl)-1*H*-1,2,3-triazol-4-yl)methyl)-1*H*-benzo[*d*]imidazol-2-yl)butyl)isoindoline-1,3-dione** (**6t**)**:** Yellow solid; m.p. 86–88 °C; Yield: 77%. HRMS *m/z* calcd. (M + H)^+^ 581.5081, found 581.1716. ^1^H NMR (400 MHz, CDCl_3_): δ 7.82 (s, 2H), 7.74 (s, 2H), 7.65 (s, 2H), 7.56 (s, 1H), 7.37 (s, 1H), 7.23 (s, 2H), 5.71 (dd, *J* = 45.9, 26.5 Hz, 2H), 5.38 (s, 1H), 3.44 (s, 1H), 1.50 (s, 1H), 1.22 (s, 1H), 0.93 (d, *J* = 16.9 Hz, 6H). ^13^C NMR (100 MHz, CDCl_3_): δ 167.85, 150.52, 150.31, 143.89, 142.55, 141.05, 134.61, 134.32, 131.37, 124.48, 123.77, 123.56, 122.76, 120.77, 109.70, 51.94, 51.75, 38.94, 34.44, 33.92, 26.99, 25.50, 16.83, 15.48, 11.04, 10.57.

**2-(2-Methyl-1-(1-((1-(pyridin-4-yl)-1*H*-1,2,3-triazol-4-yl)methyl)-1*H*-benzo[*d*]imidazol-2-yl)butyl)isoindoline-1,3-dione** (**6u**)**:** White solid; m.p. 101–103 °C; Yield: 42%. HRMS *m/z* calcd. (M + H)^+^ 492.5438, found 492.2151. ^1^H NMR (400 MHz, CDCl_3_): δ 8.63 (brs, 1H), 7.80 (s, 1H), 7.71 (d, *J* = 6.1 Hz, 1H), 7.60 (s, 2H), 7.49 (s, 3H), 7.32 (d, *J* = 5.9 Hz, 2H), 7.31 (s, 2H), 5.67–5.48 (m, 2H), 5.38 (dd, *J* = 10.1, 4.1 Hz, 1H), 3.36 (s, 1H), 1.57 (d, *J* = 80.9 Hz, 1H), 1.26–1.11 (m, 1H), 1.05 (d, *J* = 6.0 Hz, 1H), 0.94–0.87 (m, 6H). ^13^C NMR (100 MHz, CDCl_3_): δ 167.60, 167.55, 150.31, 150.17, 144.61, 142.48, 141.17, 134.93, 134.78, 134.22, 131.08, 127.54, 123.54, 123.41, 122.74, 120.73, 119.58, 109.61, 51.91, 51.47, 39.39, 34.51, 34.03, 27.16, 25.42, 17.17, 15.41, 11.19, 10.73.

**2-(1-(1-((1-(2-Fluorophenyl)-1*H*-1,2,3-triazol-4-yl)methyl)-1*H*-benzo[*d*]imidazol-2-yl)-2-methylbutyl)isoindoline-1,3-dione** (**6v**)**:** Yellow solid; m.p. 85–87 °C; Yield: 79%. HRMS *m/z* calcd. (M + H)^+^ 509.5462, found 509.2094. ^1^H NMR (400 MHz, CDCl_3_): δ 7.88–7.86 (m, 1H), 7.73 (d, *J* = 3.1 Hz, 2H), 7.61 (s, 4H), 7.39–7.36 (m, 2H), 7.25–7.15 (m, 4H), 5.75–5.52 (m, 2H), 5.42 (d, *J* = 10.7 Hz, 1H), 3.45 (brs, 1H), 1.56 (d, *J* = 42.6 Hz, 1H), 1.17 (s, 1H), 1.02–0.91 (m, 6H). ^13^C NMR (100 MHz, CDCl_3_): δ 167.70, 153.08 (d, *J* = 253.3 Hz), 150.36 (d, *J* = 16.5 Hz), 143.63, 142.56, 134.88, 134.75, 134.63, 134.16, 131.33, 130.42 (d, *J* = 7.8 Hz), 125.20 (d, *J* = 3.4 Hz), 124.84, 124.67, 123.79, 123.51, 123.46, 122.84, 122.65, 120.73, 117.02 (d, *J* = 19.9 Hz), 109.80, 51.92, 51.63, 39.26, 34.45, 33.95, 27.11, 25.48, 17.03, 15.46, 11.14, 10.68.

**2-(1-(1-((1-(2,4-Difluorophenyl)-1*H*-1,2,3-triazol-4-yl)methyl)-1*H*-benzo[*d*]imidazol-2-yl)-2-methylbutyl)isoindoline-1,3-dione (6w):** Yellow solid; m.p. 86–88 °C; Yield: 75%. HRMS *m/z* calcd. (M + H)^+^ 527.5367, found 527.1997. ^1^H NMR (400 MHz, CDCl_3_): δ 7.88 (s, 1H), 7.74 (s, 2H), 7.64 (s, 4H), 7.41 (s, 1H), 7.25 (s, 2H), 7.02–6.91 (q, 2H), 5.82–5.51 (m, 2H), 5.44 (d, *J* = 10.4 Hz, 1H), 3.44 (brs, 1H), 1.55 (brs, 1H), 1.24–1.06 (m, 1H), 1.04–0.87 (m, 6H). ^13^C NMR (100 MHz, CDCl_3_): δ 167.72, 162.53 (d, *J* = 264.4 Hz), 153.60 (d, *J* = 266.1 Hz), 150.51, 142.41, 134.66, 134.26, 131.35, 126.08 (d, *J* = 9.4 Hz), 123.58, 122.88, 120.68, 112.64 (d, *J* = 22.6 Hz), 109.94, 105.66, 105.41, 105.16, 53.59, 51.80, 51.59, 39.25, 34.59, 34.02, 27.05, 25.51, 16.95, 15.51, 11.15, 10.58.

**2-(1-(1-((1-(2,6-Difluorophenyl)-1*H*-1,2,3-triazol-4-yl)methyl)-1*H*-benzo[*d*]imidazol-2-yl)-2-methylbutyl)isoindoline-1,3-dione (6x):** White solid; m.p. 59–61 °C; Yield: 43%. HRMS *m/z* calcd. (M + H)^+^ 527.5367, found 527.2021. ^1^H NMR (400 MHz, CDCl_3_): δ 7.87 (s, 1H), 7.78 (s, 2H), 7.66 (s, 2H), 7.44 (d, *J* = 19.2 Hz, 2H), 7.25 (d, *J* = 6.1 Hz, 3H), 7.04 (dd, *J* = 13.9, 7.5 Hz, 2H), 5.83–5.73 (m, 1H), 5.62–5.58 (m, 1H), 5.41 (dd, *J* = 10.2, 5.1 Hz, 1H), 3.50–3.41 (m, 1H), 2.15 (brs, 1H), 1.51 (s, 1H), 0.98–0.89 (m, 6H). ^13^C NMR (100 MHz, CDCl_3_): δ 167.84, 157.98, 155.44, 150.42, 143.31, 142.61, 134.19, 131.45, 123.58, 120.82, 120.74, 112.62, 112.42, 109.97, 109.66, 52.03, 51.84, 39.20, 34.46, 33.91, 16.79, 15.46, 11.01, 10.61.

**2-(1-(1-((1-(4-Fluorophenyl)-1*H*-1,2,3-triazol-4-yl)methyl)-1*H*-benzo[*d*]imidazol-2-yl)ethyl)isoindoline-1,3-dione** (**6y**)**:** White solid; m.p. 241–243 °C; Yield: 74%. HRMS *m/z* calcd. (M + H)^+^ 467.4665, found 467.1627. ^1^H NMR (400 MHz, CDCl_3_): δ 7.91 (d, *J* = 8.0 Hz, 1H), 7.61–7.49 (m, 4H), 7.39–7.25 (m, 5H), 7.19 (s, 1H), 7.10 (t, *J* = 8.3 Hz, 2H), 5.88 (q, *J* = 6.8 Hz, 1H), 5.47 (s, 2H), 2.09 (d, *J* = 6.9 Hz, 3H). ^13^C NMR (100 MHz, CDCl_3_): δ 167.24, 162.39 (d, *J* = 249.5 Hz), 151.19, 144.16, 142.33, 135.59, 134.11, 132.63, 131.13, 123.70, 123.22, 122.78, 121.99 (d, *J* = 8.1 Hz), 120.77, 119.29 (d, *J* = 6.1 Hz), 116.60 (d, *J* = 22.8 Hz), 109.14, 42.80, 42.74, 39.87, 17.10.

**2-(1-(1-((1-(p-Tolyl)-1*H*-1,2,3-triazol-4-yl)methyl)-1*H*-benzo[*d*]imidazol-2-yl)ethyl)isoindoline-1,3-dione** (**6z**)**:** Yellow solid; m.p. 118–120 °C; Yield: 65%. HRMS *m/z* calcd. (M + H)^+^ 463.5026, found 463.1884. ^1^H NMR (400 MHz, CDCl_3_): δ 7.92 (d, *J* = 8.4 Hz, 1H), 7.62–7.48 (m, 4H), 7.36–7.25 (m, 5H), 7.23–7.14 (m, 4H), 5.88 (q, 1H), 5.47 (s, 2H), 2.37 (s, 3H), 2.09 (d, *J* = 6.9 Hz, 3H). ^13^C NMR (100 MHz, CDCl_3_): δ 167.26, 151.24, 143.79, 142.30, 139.00, 135.63, 134.09, 131.08, 130.08, 123.67, 123.24, 122.75, 120.73, 119.95, 119.07, 109.19, 42.73, 39.98, 21.14, 17.10.

**2-(1-(1-((1-Phenyl-1*H*-1,2,3-triazol-4-yl)methyl)-1*H*-benzo[*d*]imidazol-2-yl)ethyl)isoindoline-1,3-dione** (**6a’**)**:** Brown solid; m.p. 187–189 °C; Yield: 51%. HRMS *m/z* calcd. (M + H)^+^ 449.4760, found 449.1713. ^1^H NMR (400 MHz, CDCl_3_): δ 7.88 (dd, *J* = 6.0, 2.0 Hz, 1H), 7.61 (d, *J* = 5.2 Hz, 2H), 7.54 (d, *J* = 3.1 Hz, 2H), 7.47–7.31 (m, 8H), 5.91 (dd, *J* = 13.8, 6.8 Hz, 1H), 5.52 (d, J= 6.6 Hz, 2H), 2.13 (d, *J* = 6.9 Hz, 3H). ^13^C NMR (100 MHz, CDCl_3_): δ 167.23, 151.21, 143.84, 141.95, 136.26, 135.46, 134.12, 131.04, 129.61, 128.90, 123.80, 123.23, 122.91, 120.54, 120.00, 119.14, 109.28, 42.67, 39.92, 21.12, 17.03.

**2-(1-(1-((1-(Perfluorophenyl)-1*H*-1,2,3-triazol-4-yl)methyl)-1*H*-benzo[*d*]imidazol-2-yl)ethyl)isoindoline-1,3-dione** (**6b’**)**:** White solid; m.p. 215–217 °C; Yield: 62%. HRMS *m/z* calcd. (M + H)^+^ 539.4283, found 539.1247. ^1^H NMR (400 MHz, CDCl_3_): δ 7.88 (dd, *J* = 6.0, 2.0 Hz, 1H), 7.76–7.73 (m, 2H), 7.69–7.67 (m, 2H), 7.42 (s, 1H), 7.29 (dd, *J* = 7.9, 3.1 Hz, 3H), 5.91 (q, 1H), 5.52 (d, *J* = 6.6 Hz, 2H), 2.10 (d, *J* = 6.9 Hz, 3H). ^13^C NMR (100 MHz, CDCl_3_): δ 167.45, 151.15, 143.54, 142.31, 140.87, 139.25, 136.70, 135.25, 134.32, 131.49, 124.28, 123.65, 123.41, 122.78, 120.70, 109.28, 42.82, 39.33, 17.11.

**2-(1-(1-((1-(2-Fluorophenyl)-1*H*-1,2,3-triazol-4-yl)methyl)-1*H*-benzo[*d*]imidazol-2-yl)ethyl)isoindoline-1,3-dione** (**6c’**)**:** White solid; m.p. 186–188 °C; Yield: 64%. HRMS *m/z* calcd. (M + H)^+^ 467.4665, found 467.1624. ^1^H NMR (400 MHz, CDCl_3_): δ 7.82 (s, 1H), 7.62–7.56 (m, 3H), 7.55–7.47 (m, 3H), 7.29 (s, 1H), 7.23 (s, 2H), 7.15 (d, *J* = 6.3 Hz, 2H), 5.87 (d, *J* = 4.5 Hz, 1H), 5.44 (s, 2H), 2.06 (d, *J* = 4.5 Hz, 3H).^13^C NMR (100 MHz, CDCl_3_): δ 167.28, 150.98, 142.76 (d, *J* = 120.9 Hz), 135.47, 134.11, 131.37, 130.42 (d, *J* = 8.0 Hz), 125.12 (d, *J* = 3.4 Hz), 124.51, 123.63, 123.38, 123.33, 122.75, 122.51 (d, *J* = 8.3 Hz), 120.58, 117.04 (d, *J* = 19.8 Hz), 42.74, 39.65, 17.09.

**2-(1-(1-((1-(2,4-Difluorophenyl)-1*H*-1,2,3-triazol-4-yl)methyl)-1*H*-benzo[*d*]imidazol-2-yl)ethyl)isoindoline-1,3-dione** (**6d’**)**:** White solid; m.p. 195–197 °C; Yield: 43%. HRMS *m/z* calcd. (M + H)^+^ 485.4569, found 485.1532. ^1^H NMR (400 MHz, CDCl_3_): δ 7.82 (d, *J* = 28.8 Hz, 2H), 7.66 (s, 2H), 7.58 (s, 2H), 7.45 (d, *J* = 19.8 Hz, 2H), 7.35–7.16 (m, 3H), 6.93 (s, 1H), 5.89 (s, 1H), 5.46 (s, 2H), 2.07 (s, 3H). ^13^C NMR (100 MHz, CDCl_3_): δ 167.25, 151.18, 142.85 (d, *J* = 129.4 Hz), 135.41, 134.10, 131.41, 125.80, 123.59, 123.31), 122.60 (d, *J* = 23.1 Hz), 120.61, 112.49 (d, *J* = 18.8 Hz), 109.29, 105.40, 42.74, 39.55, 17.08.

**2-(1-(1-((1-Benzyl-1*H*-1,2,3-triazol-4-yl)methyl)-1*H*-benzo[*d*]imidazol-2-yl)ethyl)isoindoline-1,3-dione** (**6e’**)**:** Yellow solid; m.p. 181–183 °C; Yield: 61%. HRMS *m/z* calcd. (M + H)^+^ 463.5026, found 463.1881. ^1^H NMR (400 MHz, CDCl_3_): δ 7.85 (d, *J* = 7.5 Hz, 1H), 7.65 (s, 4H), 7.39–7.20 (m, 7H), 7.07 (d, *J* = 4.3 Hz, 2H), 6.98 (s, 1H), 5.85 (q, 1H), 5.38 (s, 2H), 5.12 (q, *J* = 31.3, 14.9 Hz, 2H), 2.06 (d, *J* = 6.9 Hz, 3H). ^13^C NMR (100 MHz, CDCl_3_): δ 167.32, 151.25, 143.30, 142.25, 135.45, 134.19, 131.51, 129.11, 128.80, 127.97, 123.43, 123.27, 122.56, 121.45, 120.53, 109.48, 54.00, 42.78, 39.74, 17.17.

**2-(1-(1-((1-(2,6-Difluorophenyl)-1*H*-1,2,3-triazol-4-yl)methyl)-1*H*-benzo[*d*]imidazol-2-yl)ethyl)isoindoline-1,3-dione** (**6f’**)**:** Green solid; m.p. 94–96 °C; Yield: 54%. HRMS *m/z* calcd. (M + H)^+^ 485.4569, found 485.1531. ^1^H NMR (400 MHz, CDCl_3_): δ 7.67 (d, *J* = 34.1 Hz, 1H), 7.39 (s, 3H), 7.24 (s, 2H), 7.00 (s, 3H), 5.92 (s, 1H), 5.52 (s, 2H), 2.11 (s, 3H). ^13^C NMR (100 MHz, CDCl_3_): δ 167.49, 156.48 (d, *J* = 256.5 Hz), 134.35, 131.59 (d, *J* = 20.5 Hz), 128.86, 126.99, 123.81, 123.49, 120.58, 114.76, 112.57 (d, *J* = 19.0 Hz), 29.76, 22.77, 17.05, 14.24.

**2-(1-(1-((1H-1,2,3-Triazol-4-yl)methyl)-1*H*-benzo[*d*]imidazol-2-yl)ethyl)isoindoline-1,3-dione** (**6g’**)**:** White solid; m.p. 97–99 °C; Yield: 57%. HRMS *m/z* calcd. (M + H)^+^ 373.3800, found 373.1433. ^1^H NMR (400 MHz, DMSO): δ 7.77–7.73 (m, 2H), 7.71–7.67 (m, 2H), 7.63 (d, *J* = 4.9 Hz, 1H), 7.48–7.29 (m, 2H), 7.20–7.12 (m, 2H), 5.90 (s, 1H), 5.37 (dd, *J* = 41.7, 16.5 Hz, 2H), 1.86 (d, *J* = 6.6 Hz, 3H). ^13^C NMR (100 MHz, DMSO): δ 167.66, 152.15, 142.16, 136.15, 134.90, 131.76, 123.44, 123.08, 122.30, 119.77, 110.91, 79.71, 42.85, 17.25.

**2-(1-(1-((1-(4-Fluorophenyl)-1*H*-1,2,3-triazol-4-yl)methyl)-1*H*-benzo[*d*]imidazol-2-yl)ethyl)-5-methylisoindoline-1,3-dione** (**6h’**)**:** White solid; m.p. 222–224 °C; Yield: 64%. HRMS *m/z* calcd. (M + H)^+^ 481.4930, found 481.1785. ^1^H NMR (400 MHz, CDCl_3_): δ 7.88 (d, *J* = 7.6 Hz, 1H), 7.44 (d, *J* = 7.8 Hz, 1H), 7.35–7.21 (m, 8H), 7.08 (dd, *J* = 16.9, 8.7 Hz, 3H), 5.82 (q, *J* = 6.7 Hz, 1H), 5.43 (s, 2H), 2.25 (s, 3H), 2.04 (d, *J* = 6.9 Hz, 3H). ^13^C NMR (100 MHz, CDCl_3_): δ 167.21, 151.31, 144.82 (d, *J* = 125.8 Hz), 142.28, 135.60, 134.63, 131.99 (d, *J* = 124.4 Hz), 128.48, 123.64, 122.97 (d, *J* = 45.8 Hz), 121.83 (d, *J* = 8.4 Hz), 119.12, 116.54 (d, *J* = 23.2 Hz), 109.12, 42.62, 39.89, 31.01, 21.89, 17.05.

**5-Methyl-2-(1-(1-((1-(p-tolyl)-1*H*-1,2,3-triazol-4-yl)methyl)-1*H*-benzo[*d*]imidazol-2-yl)ethyl)isoindoline-1,3-dione** (**6i’**)**:** White solid; m.p. 216–218 °C; Yield: 83%. HRMS *m/z* calcd. (M + H)^+^ 477.5292, found 477.2035. ^1^H NMR (400 MHz, CDCl_3_): δ 7.87 (d, *J* = 7.8 Hz, 1H), 7.42 (d, *J* = 8.1 Hz, 1H), 7.24 (m, 5H), 7.15 (d, *J* = 2.4 Hz, 4H), 7.10 (s, 1H), 5.80 (q, *J* = 6.9 Hz, 1H), 5.40 (d, *J* = 2.7 Hz 2H), 2.33 (s, 3H), 2.20 (s, 3H), 2.03 (d, *J* = 7.0 Hz, 3H). ^13^C NMR (100 MHz, CDCl_3_): δ 167.35, 167.16, 151.38, 145.38, 143.75, 142.21, 138.82, 135.64, 134.58, 134.03, 131.28, 129.99, 128.40, 123.57, 123.16, 122.63, 120.55, 119.68, 118.83, 109.23, 42.53, 39.91, 21.84, 21.09, 17.03.

**5-Methyl-2-(1-(1-((1-(4-(trifluoromethyl)phenyl)-1*H*-1,2,3-triazol-4-yl)methyl)-1*H*-benzo[*d*]imidazol-2-yl)ethyl)isoindoline-1,3-dione (6j’):** White solid; m.p. 265–267 °C; Yield: 74%. HRMS *m/z* calcd. (M + H)^+^ 477.5292, found 477.2035. ^1^H NMR (400 MHz, CDCl_3_): δ 7.91 (d, *J* = 7.6 Hz, 1H), 7.67 (d, *J* = 8.5 Hz, 2H), 7.50 (d, *J* = 8.5 Hz, 2H), 7.43 (d, *J* = 8.1 Hz, 1H), 7.34–7.22 (m, 6H), 5.83 (q, *J* = 6.9 Hz, 1H), 5.46 (s, 2H), 2.21 (s, 3H), 2.06 (d, *J* = 7.0 Hz, 3H). ^13^C NMR (100 MHz, CDCl_3_): δ 167.39, 167.18, 151.31, 145.50, 144.68, 142.35, 138.69, 135.60, 134.64, 131.35, 128.43, 126.94, 123.75, 123.60, 123.23, 122.84, 120.84, 119.85, 118.91, 109.05, 42.66, 39.89, 21.81, 17.07.

**2-(1-(1-((1*H*-1,2,3-Triazol-4-yl)methyl)-1*H*-benzo[*d*]imidazol-2-yl)ethyl)-5-methylisoindoline-1,3-dione** (**6k’**)**:** Brown solid; m.p. 100–102 °C; Yield: 54%. HRMS *m/z* calcd. (M + H)^+^ 387. 4066, found 387.1605. ^1^H NMR (400 MHz, CDCl_3_): δ 7.86 (d, *J* = 7.1 Hz, 1H), 7.54 (d, *J* = 7.5 Hz, 1H), 7.47 (s, 1H), 7.32 (d, *J* = 7.5 Hz, 1H), 7.25 (t, *J* = 6.5 Hz, 4H), 5.84 (q, *J* = 13.4, 6.5 Hz 1H), 5.41 (s, 2H), 2.35 (s, 3H), 2.04 (d, *J* = 6.8 Hz, 3H). ^13^C NMR (100 MHz, CDCl_3_): δ 167.54, 151.35, 145.59, 141.88, 135.44, 134.80, 131.76, 128.81, 123.91, 123.63, 123.34, 122.80, 120.45, 109.55, 42.49, 39.43, 21.96, 17.08.

**5-Methyl-2-(1-(1-((1-phenyl-1*H*-1,2,3-triazol-4-yl)methyl)-1*H*-benzo[*d*]imidazol-2-yl)ethyl)isoindoline-1,3-dione** (**6l’**)**:** White solid; m.p. 185–187 °C; Yield: 63%. HRMS *m/z* calcd. (M + H)^+^ 463.5026, found 463.1893. ^1^H NMR (400 MHz, CDCl_3_): δ 7.89 (d, *J* = 7.8 Hz, 1H), 7.43 (d, *J* = 8.1 Hz, 1H), 7.40–7.34 (m, 3H), 7.33–7.22 (m, 8H), 7.16 (s, 1H), 5.82 (q, *J* = 6.9 Hz, 1H), 5.48–5.37 (m, 2H), 2.20 (s, 3H), 2.05 (d, *J* = 6.9 Hz, 3H). ^13^C NMR (100 MHz, CDCl_3_): δ 167.32, 167.14, 151.31, 145.40, 143.92, 142.16, 136.23, 135.60, 134.57, 131.21, 129.53, 128.70, 128.34, 123.58, 123.15, 122.67, 120.58, 119.73, 118.82, 109.15, 42.50, 39.90, 21.79, 16.99.

**2-(1-(1-((1-Benzyl-1*H*-1,2,3-triazol-4-yl)methyl)-1*H*-benzo[*d*]imidazol-2-yl)ethyl)-5-methylisoindoline-1,3-dione** (**6m’**)**:** Green solid; m.p. 142–144 °C; Yield: 74%. HRMS *m/z* calcd. (M+H)^+^ 477.5292, found 477.2048. ^1^H NMR (400 MHz, CDCl_3_): δ 7.76 (d, *J* = 7.3 Hz, 1H), 7.51–7.33 (m, 3H), 7.32–7.08 (m, 7H), 7.08–6.91 (m, 3H), 5.77 (d, *J* = 6.8 Hz, 1H), 5.29 (s, 2H), 5.07 (q, *J* = 15.0 Hz, 2H), 2.40 (s, 3H), 1.99 (d, *J* = 6.7 Hz, 3H). ^13^C NMR (100 MHz, CDCl_3_): δ 167.50, 167.40, 151.36, 145.50, 143.28, 142.16, 135.39, 134.79, 134.29, 131.89, 129.07, 128.94, 128.74, 127.90, 123.83, 123.39, 123.19, 122.52, 121.61, 120.41, 109.60, 53.95, 42.72, 39.68, 22.12, 17.15.

**5-Methyl-2-(1-(1-((1-(pyridin-4-yl)-1*H*-1,2,3-triazol-4-yl)methyl)-1*H*-benzo[*d*]imidazol-2-yl)ethyl)isoindoline-1,3-dione** (**6n’**)**:** White solid; m.p. 236–238 °C; Yield: 75%. HRMS *m/z* calcd. (M + H)^+^ 463.4906, found 464.1820. ^1^H NMR (400 MHz, CDCl_3_): δ 8.63 (d, *J* = 5.2 Hz, 2H), 7.91 (d, *J* = 8.0 Hz, 1H), 7.72 (d, *J* = 8.1 Hz, 1H), 7.48 (d, *J* = 7.5 Hz, 1H), 7.41–7.36 (m, 1H), 7.34–7.27 (m, 6H), 5.86 (q, *J* = 6.9 Hz, 1H), 5.49 (s, 2H), 2.29 (s, 3H), 2.08 (d, *J* = 7.0 Hz, 3H). ^13^C NMR (100 MHz, CDCl_3_): δ 167.37, 167.20, 151.31, 149.95, 145.65, 144.64, 142.31, 141.25, 135.57, 134.70, 131.43, 128.49, 127.26, 124.01, 123.72, 123.67, 123.24, 122.82, 120.79, 119.08, 109.09, 42.65, 39.85, 21.91, 17.07.

**2-(1-(1-((1-(2-Fluorophenyl)-1*H*-1,2,3-triazol-4-yl)methyl)-1*H*-benzo[*d*]imidazol-2-yl)ethyl)-5-methylisoindoline-1,3-dione** (**6o’**)**:** White solid; m.p. 185–187 °C; Yield: 64%. ^1^H NMR (400 MHz, CDCl_3_): δ 7.87 (d, *J* = 7.4 Hz, 1H), 7.51 (dd, *J* = 13.7, 7.5 Hz, 2H), 7.41 (d, *J* = 12.7 Hz, 2H), 7.36–7.23 (m, 5H), 7.17 (dd, *J* = 17.5, 9.2 Hz, 2H), 5.86 (q, *J* = 13.6, 6.7 Hz, 1H), 5.46 (s, 2H), 2.31 (s, 3H), 2.06 (d, *J* = 6.9 Hz, 3H). ^13^C NMR (100 MHz, CDCl_3_): δ 167.38, 167.27, 152.89 (d, *J* = 251.9 Hz), 151.32, 145.43, 143.50, 142.27, 135.59, 134.65, 131.66, 130.33 (d, *J* = 7.7 Hz), 128.76, 125.08, 125.05, 124.67 (d, *J* = 9.9 Hz), 124.35, 123.78, 123.58, 123.31, 122.67, 122.35 (d, *J* = 8.6 Hz), 120.65, 117.05 (d, *J* = 20.0 Hz), 109.32, 42.63, 39.71, 21.96, 17.07.

**2-(1-(1-((1-(2,4-Difluorophenyl)-1*H*-1,2,3-triazol-4-yl)methyl)-1*H*-benzo[*d*]imidazol-2-yl)ethyl)-5-methylisoindoline-1,3-dione** (**6p’**)**:** White solid; m.p. 206–208 °C; Yield: 52%. HRMS *m/z* calcd. (M + H)^+^ 499.4835, found 499.1699. ^1^H NMR (400 MHz, CDCl_3_): δ 7.85 (d, *J* = 7.5 Hz, 1H), 7.53 (d, *J* = 7.5 Hz, 1H), 7.47 (d, *J* = 5.9 Hz, 1H), 7.40 (d, *J* = 5.8 Hz, 2H), 7.35 (d, *J* = 7.5 Hz, 1H), 7.31–7.21 (m, 3H), 6.93 (t, *J* = 8.1 Hz, 2H), 5.84 (q, 1H), 5.45 (s, 2H), 2.35 (s, 3H), 2.05 (d, *J* = 6.6 Hz, 3H). ^13^C NMR (100 MHz, CDCl_3_): δ 167.38, 167.27, 154.14, 151.64, 151.32, 144.54 (d, *J* = 183.5 Hz), 142.27, 135.59, 134.65, 131.66, 130.36, 130.29, 128.76, 125.77 (d, *J* = 9.8 Hz), 124.72, 124.62, 124.35, 123.78, 123.58, 123.31, 122.67, 122.36 (d, *J* = 7.5 Hz), 120.65, 117.15, 116.95, 112.46 (d, *J* = 19.5 Hz), 109.32, 42.63, 39.71, 21.96, 17.07.

**2-(1-(1-((1-(2,6-Difluorophenyl)-1*H*-1,2,3-triazol-4-yl)methyl)-1*H*-benzo[*d*]imidazol-2-yl)ethyl)-5-methylisoindoline-1,3-dione** (**6q’**)**:** Green solid; m.p.166–168 °C; Yield: 52%. HRMS *m/z* calcd. (M + H)^+^ 499.4835, found 499.1698. ^1^H NMR (400 MHz, CDCl_3_): δ 7.87 (s, 1H), 7.60 (d, *J* = 7.5 Hz, 1H), 7.50 (s, 1H), 7.41 (d, *J* = 7.9 Hz, 2H), 7.35 (s, 2H), 7.29–7.24 (m, 2H), 7.02 (t, *J* = 8.4 Hz, 2H), 5.90 (d, *J* = 6.4 Hz, 1H), 5.52 (s, 2H), 2.41 (s, 3H), 2.10 (d, *J* = 6.3 Hz, 3H). ^13^C NMR (100 MHz, CDCl_3_): δ 167.62, 167.51, 156.45 (d, *J* = 257.1 Hz), 145.71, 134.90, 131.67 (d, *J* = 18.4 Hz), 128.83, 123.94 (d, *J* = 17.2 Hz), 123.43, 122.90, 120.52, 114.72, 112.52 (d, *J* = 20.4 Hz), 109.71, 42.58, 39.63, 29.77, 22.08, 17.14, 14.24.

**2-(2-Methyl-1-(1-((1-(perfluorophenyl)-1*H*-1,2,3-triazol-4-yl)methyl)-1*H*-benzo[*d*]imidazol-2-yl)propyl)isoindoline-1,3-dione** (**6r’**)**:** Yellow solid; m.p. 170–172 °C; Yield: 59%. HRMS *m/z* calcd. (M + H)^+^ 567.4815, found 567.1564. ^1^H NMR (400 MHz, CDCl_3_): δ 7.73 (dd, *J* = 5.1, 3.1 Hz, 1H), 7.73–7.71 (m, 2H), 7.66–7.61 (m, 2H), 7.60 (s, 1H), 7.38–7.31 (m, 1H), 7.20–7.18 (m, 2H), 5.65 (dd, *J* = 53.0, 16.7 Hz, 2H), 5.29 (d, *J* = 10.8 Hz, 1H), 3.64–3.55 (m, 1H), 0.99 (dd, *J* = 6.4, 4.1 Hz, 6H). ^13^C NMR (100 MHz, CDCl_3_): δ 167.80, 150.27, 143.80, 142.54, 134.63, 134.33, 131.34, 124.52, 123.52, 122.70, 120.70, 109.67, 53.23, 38.93, 28.45, 20.87, 19.47.

**2-(1-(1-((1-(2-Fluorophenyl)-1*H*-1,2,3-triazol-4-yl)methyl)-1*H*-benzo[*d*]imidazol-2-yl)-2-methylpropyl)isoindoline-1,3-dione** (**6s’**)**:** Yellow solid; m.p. 115–117 °C; Yield: 62%. HRMS *m/z* calcd. (M + H)^+^ 495.1937, found 495.5196. ^1^H NMR (400 MHz, CDCl_3_): δ 7.92–7.86 (m, 1H), 7.73 (dd, *J* = 5.3, 3.0 Hz, 2H), 7.64–7.57 (m, 4H), 7.44–7.32 (m, 2H), 7.29–7.14 (m, 4H), 5.63 (dd, *J* = 55.0, 16.8 Hz, 2H), 5.35 (d, *J* = 10.8 Hz, 1H), 3.69–3.59 (m, 1H), 1.10 (d, *J* = 6.6 Hz, 3H), 1.02 (d, *J* = 6.6 Hz, 3H). ^13^C NMR (100 MHz, CDCl_3_): δ 167.66, 153.05 (d, *J* = 251.2 Hz), 150.19, 143.06 (d, *J* = 115.3 Hz), 134.77, 134.18, 131.27, 130.42 (d, *J* = 7.8 Hz), 125.20, 125.17, 124.65, 123.52, 122.70, 120.74, 117.02 (d, *J* = 20.0 Hz), 109.74, 53.09, 39.31, 28.48, 21.08, 19.48.

**2-(1-(1-((1-(2,4-Difluorophenyl)-1*H*-1,2,3-triazol-4-yl)methyl)-1*H*-benzo[*d*]imidazol-2-yl)-2-methylpropyl)isoindoline-1,3-dione** (**6t’**)**:** Yellow solid; m.p. 100–102 °C; Yield: 59%. HRMS *m/z* calcd. (M + H)^+^ 513.5101, found 513.1841. ^1^H NMR (400 MHz, CDCl_3_): δ 7.84 (d, *J* = 8.0 Hz, 1H), 7.69 (dd, *J* = 4.0, 3.0 Hz, 2H), 7.62–7.47 (m, 4H), 7.36 (d, *J* = 4.7 Hz, 1H), 7.30–7.15 (m, 2H), 6.92 (d, *J* = 6.0 Hz, 2H), 5.59 (dd, *J* = 51.8, 16.8 Hz, 2H), 5.32 (d, *J* = 10.8 Hz, 1H), 3.70–3.49 (m, 1H), 1.06 (d, *J* = 6.3 Hz, 3H), 0.98 (d, *J* = 6.3 Hz, 3H). ^13^C NMR (100 MHz, CDCl_3_): δ 177.92, 167.65, 162.50 (d, *J* = 253.1 Hz), 153.49 (d, *J* = 241.9 Hz), 150.15, 143.70, 142.45, 134.76, 134.63, 134.21, 131.25, 126.00 (d, *J* = 9.9 Hz), 123.47, 122.74, 122.66, 121.43, 120.64, 112.56 (d, *J* = 19.4 Hz), 109.73, 105.61, 105.37, 105.11, 68.62, 53.62, 53.07, 39.18, 28.43, 27.85, 22.22, 21.04, 19.45.

**2-(1-(1-((1-(2,6-Difluorophenyl)-1*H*-1,2,3-triazol-4-yl)methyl)-1*H*-benzo[*d*]imidazol-2-yl)-2-methylpropyl)isoindoline-1,3-dione** (**6u’**)**:** Yellow solid; m.p. 91–93 °C; Yield: 50%. HRMS *m/z* calcd. (M + H)^+^ 513.5101, found 513.1866. ^1^H NMR (400 MHz, CDCl_3_): δ 7.84 (s, 1H), 7.75 (s, 2H), 7.64 (s, 2H), 7.42 (d, *J* = 20.8 Hz, 3H), 7.24 (s, 3H), 7.01 (t, *J* = 7.1 Hz, 2H), 5.66 (dd, *J* = 63.4, 15.7 Hz, 2H), 5.31 (d, *J* = 8.6 Hz, 1H), 3.63 (brs, 1H), 0.99 (d, *J* = 1.4 Hz, 6H). ^13^C NMR (100 MHz, CDCl_3_): δ 167.83, 156.60 (d, *J* = 255.5 Hz), 150.35, 142.78 (d, *J* = 71.6 Hz), 134.65, 134.33, 131.74, 131.65, 131.55, 131.31, 124.51, 123.59, 122.75, 120.66, 112.55 (d, *J* = 19.5 Hz), 109.86, 53.16, 39.15, 28.46, 20.85, 19.52.

#### 4.3.2. Biology

Maintenance of the *S. mansoni* (Naval Medical Research Institute (NMRI) isolate), life cycle involved male Golden Syrian Hamsters and *Biomphalaria glabrata* snails (NMRI isolate), as the definitive and intermediate hosts, respectively [40,41,47]. Use of hamsters was approved by the Institutional Animal Care and Use Committee of the University of California San Diego. The preparation of somules (post-infective larvae derived from infectious larvae called cercariae) and adult worms (≥42-days-old), and their co-incubation with test compounds using a customized Basch incubation medium were as described [40,41,48]. Screens of two developmental stages were employed in order to identify compounds with a potential broader spectrum of efficacy than the current drug, PZQ, which is deficient in this regard [49,50]. Screens with somules employed 96-well round-bottomed plates and 40–50 animals/well, whereas screens with adults employed 24-well flat-bottomed plates and approximately 5–7 males and 2–3 females/well. Screens employed a final compound concentration of 10 µM in DMSO (at 0.5% and 0.1% final concentrations for somules and adults, respectively). The complex and often dynamic phenotypic responses that the schistosome parasite is capable of were observed using an Axiovert A1 inverted microscope at the times indicated in the data table. Observations were codified using a defined nomenclature that involves simple “descriptors”, to convey changes in shape, motility and appearance compared to DMSO controls [40]. Each descriptor was given a value of 1 and these were added up to yield a “severity score” with a maximum of 4. Evidence of degeneracy or death was awarded a value of 4 for adult parasites specifically, the inability to adhere to the bottom of the well using oral or ventral suckers was given a score of 1 and damage to the tegument (surface) was awarded a value of 4 on the understanding that such damage in vivo would compromise survival [42,43]. Scores were averaged across the two experiments.

#### 4.3.3. HepG2 Cell Cytotoxicity Assay

HepG2-A16-CD81EGFP cells (human hepatocarcinoma HepG2 cells stably transformed to express the tetraspanin CD81 receptor [51,52]) were cultured at 37 °C in 5% CO_2_ in DMEM (without phenol red; Thermo Fisher Scientific, Waltham, MA, USA) supplemented with 10% FBS, 0.29 mg/mL glutamine, 100-unit penicillin and 100 μg/mL streptomycin. For cytotoxicity assays, compounds were prepared in 12-point 1:3 serial dilutions in DMSO starting at 10 mM. Then, 50 nL of compound (0.5% final DMSO concentration) were transferred with an Acoustic Transfer System (ATS) (Biosero) into white, solid bottom 1536-well assay plates (Greiner BioOne custom GNF mold; Monroe, NC, USA). Puromycin (12-point serial dilution starting at 10 μM) and 0.5% DMSO were used as positive and negative controls, respectively. After addition of compound, HepG2-A16-CD81EGFP cells in 10 µL of the above medium (5% FBS) were seeded into the assay plates at a density of 3,000 cells per well. Assay plates were incubated at 37 °C in 5% CO_2_for 72 h. To assess the HepG2 viability, the assay medium was removed by inverted centrifugation of the plate at 150 x*g* for 30 s. Then, 2 μL per well of CellTiter-Glo reagent (Promega diluted 1:2 with deionized water) was added using a MicroFlo liquid handler (BioTek). Immediately after addition of the CellTiter-Glo reagent, the luminescence was measured with an EnVision Multilabel reader (PerkinElmer). EC_50_ values were calculated using the normalized bioluminescence intensity and a non-linear, variable slope, four-parameter regression curve-fitting model in Prism 5 (GraphPad Software Inc., San Diego, CA, USA).

## Figures and Tables

**Table 1:** Bioactivity and counter toxicity data for Pht analogues **6**(**a**–**u’**).

Code	R	R^1^	R^2^	Somule Severity Score (10 µM) ^a^			Adult Severity Score (10 µM)			HepG2 (EC_50_ µM)
				24 h	48 h	72 h	1 h	5 h	24 h	72 h
**6a**	CH_3_	CH_2_CH(CH_3_)_2_	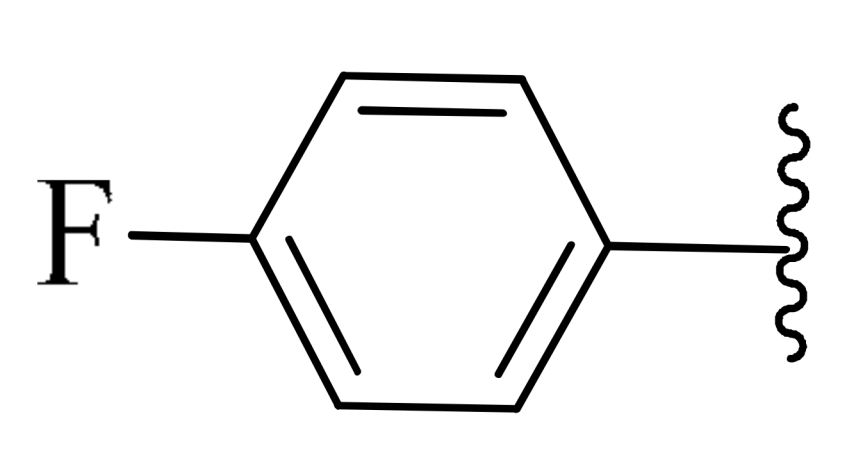	0	0	0	2	4	4	>50
**6b**	CH_3_	CH_2_CH(CH_3_)_2_	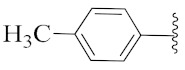	0	0	0	0	0	0	3.292
**6c**	CH_3_	CH_2_CH(CH_3_)_2_	H	0	0	0	0	0	0	73.7
**6d**	CH_3_	CH_2_CH(CH_3_)_2_	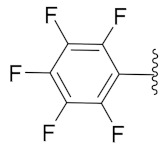	1	4	4	1	2	4	4.47
**6e**	CH_3_	CH_2_CH(CH_3_)_2_	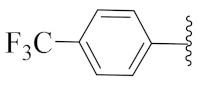	0	0	0	0	0	0	>50
**6f**	CH_3_	CH_2_CH(CH_3_)_2_	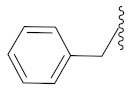	1	4	4	2	2	3	13.65
**6g**	CH_3_	CH_2_CH(CH_3_)_2_	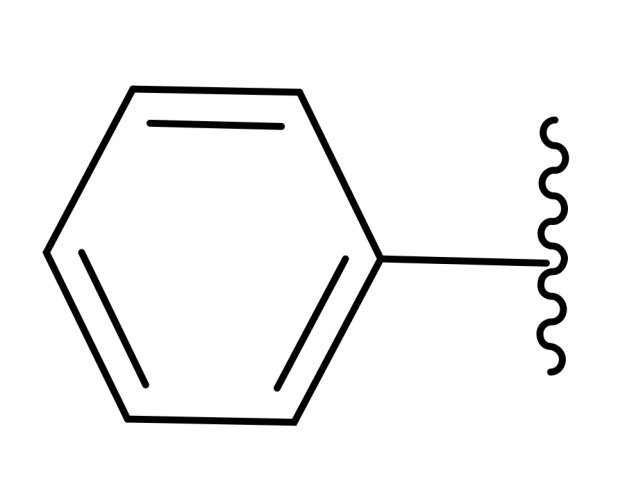	0	2	3	0	0	0	69.93
**6h**	CH_3_	CH_2_CH(CH_3_)_2_	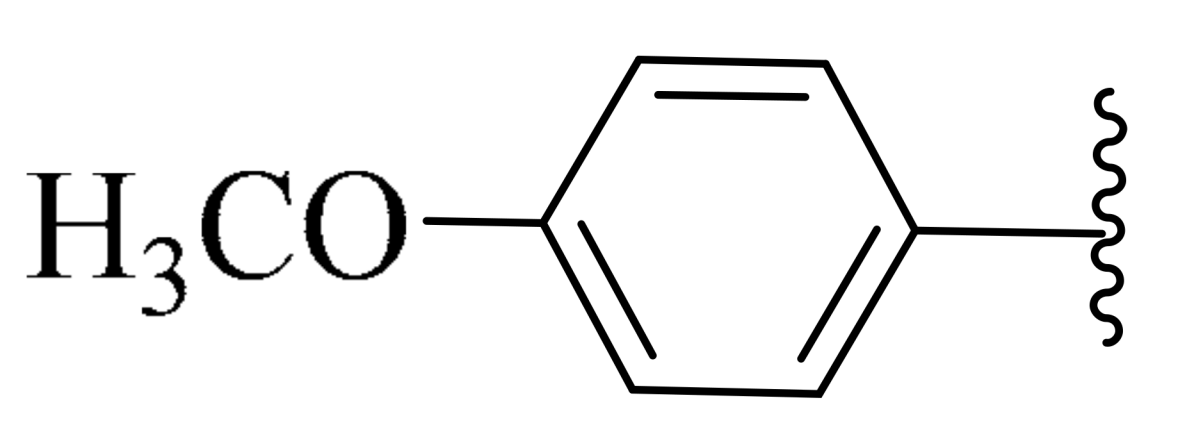	0	2	4	1	1	1	61.02
**6i**	CH_3_	CH_2_CH(CH_3_)_2_	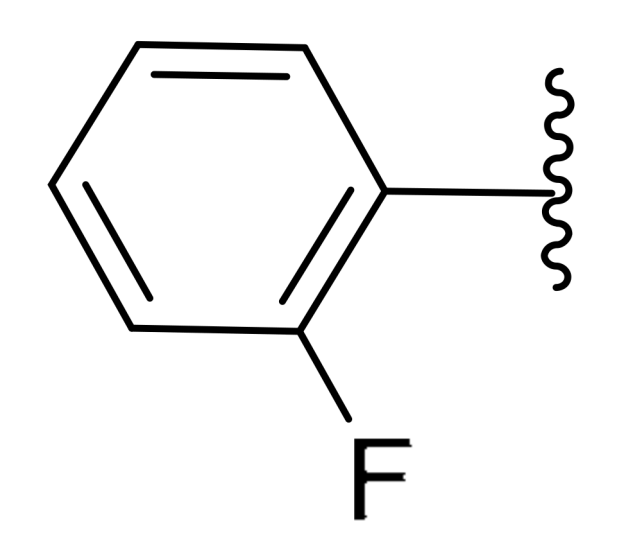	0	4	4	2	2	2	32.18
**6j**	CH_3_	CH_2_CH(CH_3_)_2_	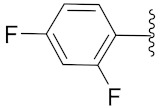	4	4	4	1	1	2	56.92
**6k**	CH_3_	CH_2_CH(CH_3_)_2_	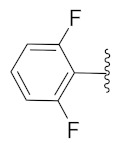	1	4	4	2	1	2	>100
**6l**	H	CH_2_CH(CH_3_)_2_	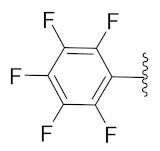	0	4	4	2	2	4	18.2
**6m**	H	CH_2_CH(CH_3_)_2_	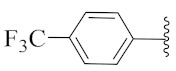	0	0	0	0	0	0	>100
**6n**	H	CH_2_CH(CH_3_)_2_	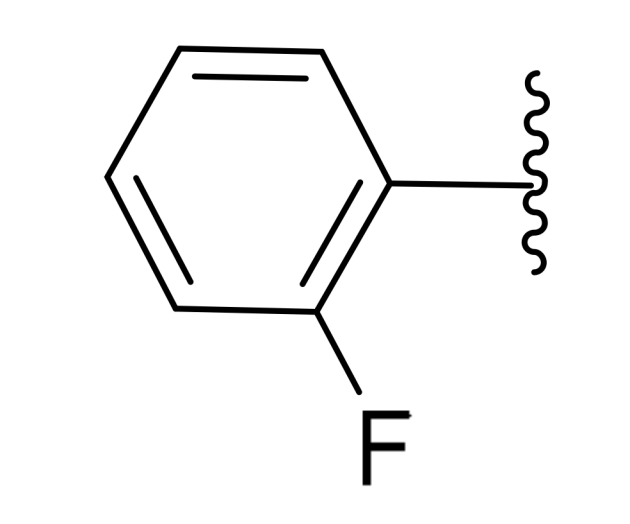	0	2	4	2	2	2	100
**6o**	H	CH_2_CH(CH_3_)_2_	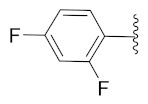	1	4	4	2	2	2	>100
**6p**	H	CH_2_CH(CH_3_)_2_	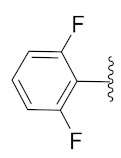	0	4	4	1	2	2	>100
**6q**	CH_3_	CH(CH_3_)CH_2_CH_3_	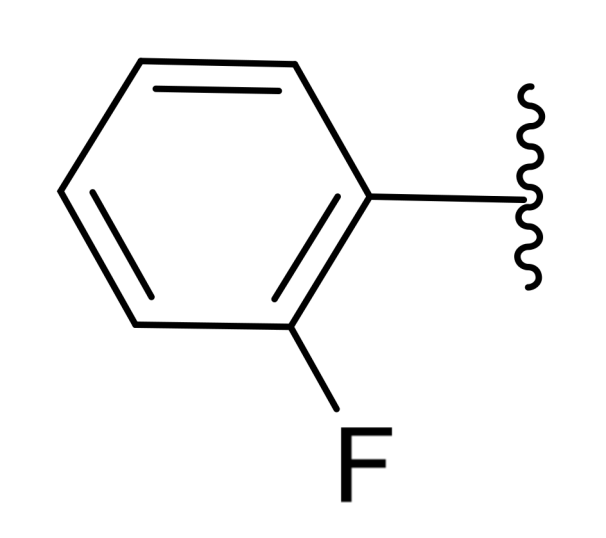	1	0	0	0	0	0	>100
**6r**	CH_3_	CH(CH_3_)CH_2_CH_3_	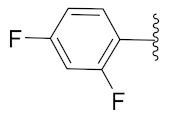	0	0	0	0	0	1	>100
**6s**	CH_3_	CH(CH_3_)CH_2_CH_3_	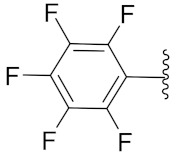	0	0	4	1	2	4	49.43
**6t**	H	CH(CH_3_)CH_2_CH_3_	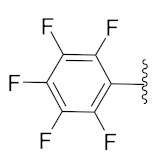	0	0	0	0	2	4	3.604
**6u**	H	CH(CH_3_)CH_2_CH_3_	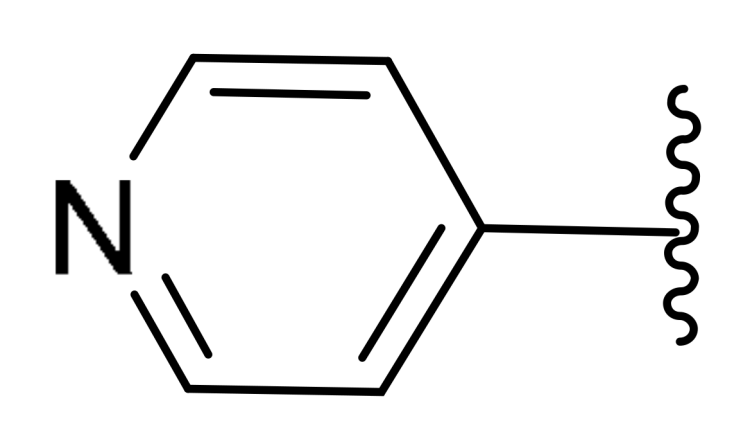	0	0	0	0	0	0	>100
**6v**	H	CH(CH_3_)CH_2_CH_3_	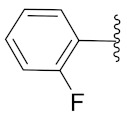	0	0	0	0	0	0	>100
**6w**	H	CH(CH_3_)CH_2_CH_3_	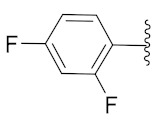	0	0	2	0	2	2	>100
**6x**	H	CH(CH_3_)CH_2_CH_3_	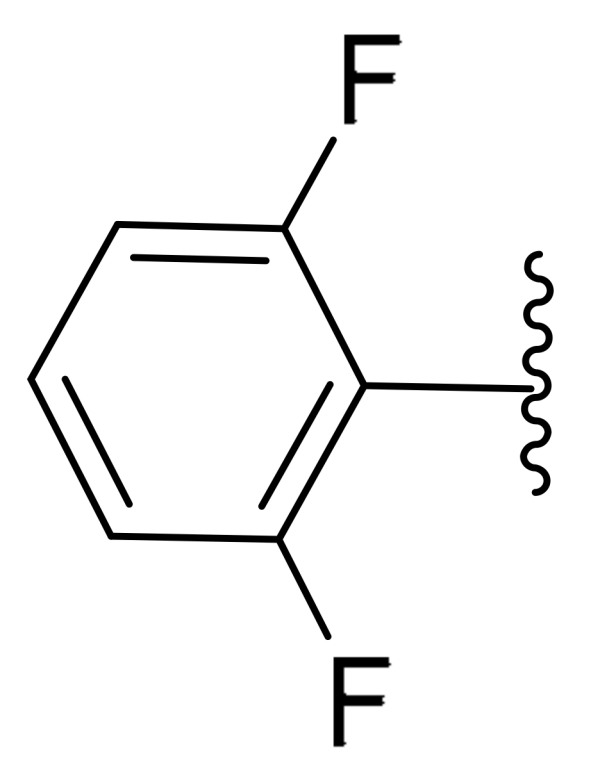	0	0	2	0	0	0	>100
**6y**	H	CH_3_	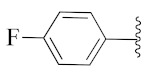	0	0	0	0	0	0	>100
**6z**	H	CH_3_	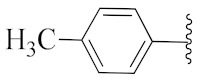	1	0	0	0	0	0	>100
**6a’**	H	CH_3_	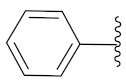	0	0	0	0	0	0	>50
**6b’**	H	CH_3_	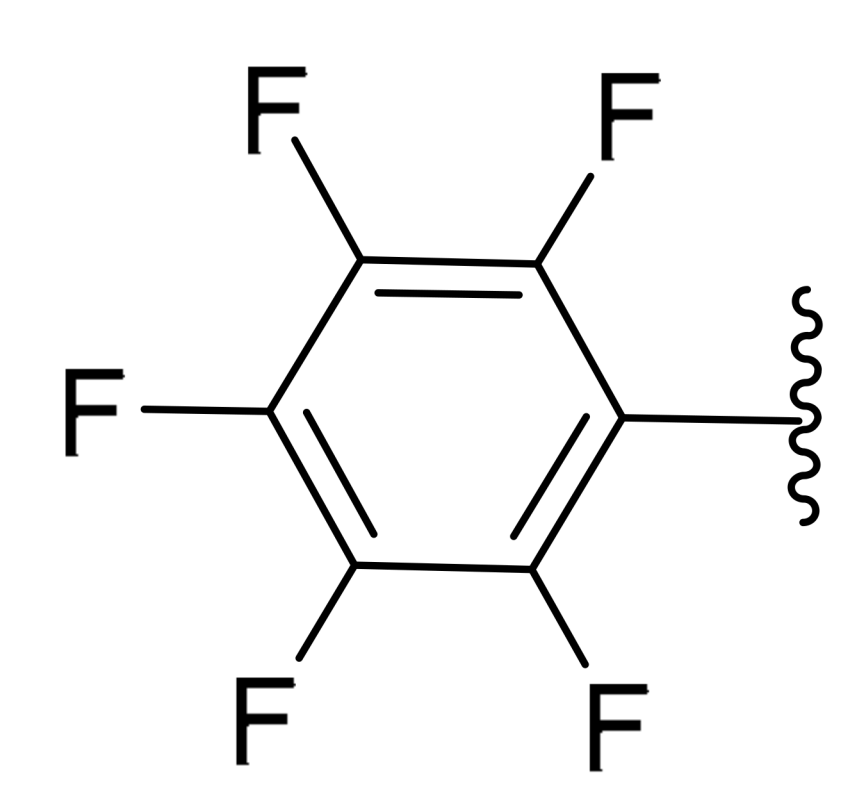	0	0	0	0	0	0	>100
**6c’**	H	CH_3_	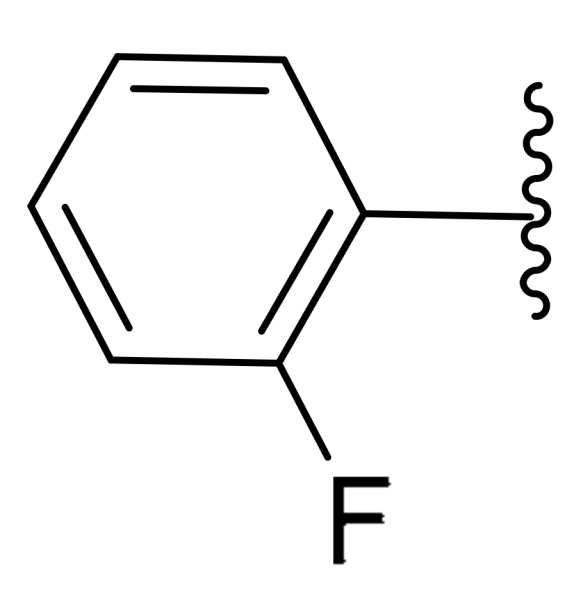	0	0	0	0	0	0	>50
**6d’**	H	CH_3_	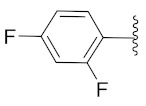	0	0	0	0	0	0	>100
**6e’**	H	CH_3_	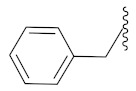	0	0	0	0	0	0	>100
**6f’**	H	CH_3_	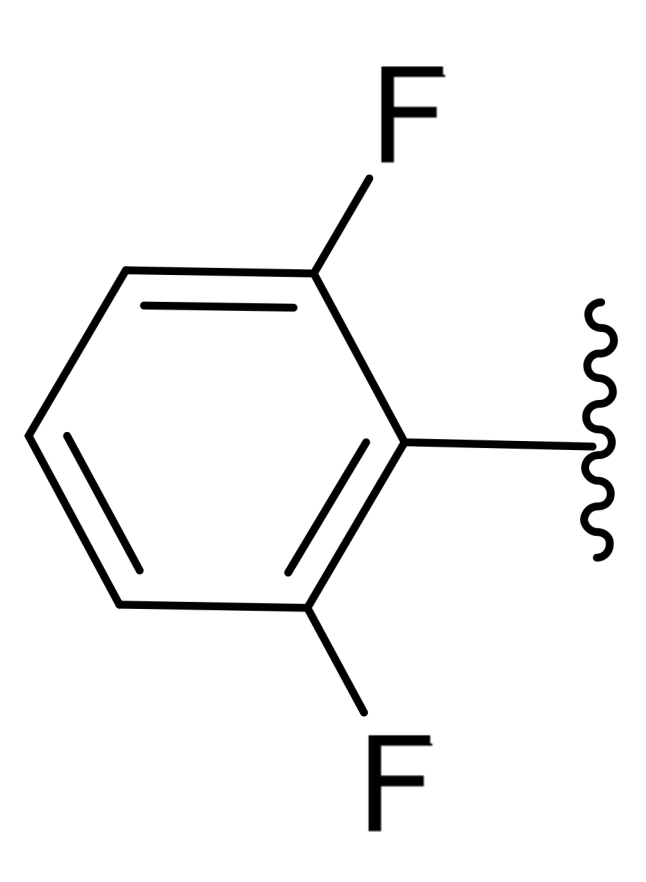	0	0	0	0	0	0	>100
**6g’**	H	CH_3_	H	0	0	0	0	0	0	>100
**6h’**	CH_3_	CH_3_	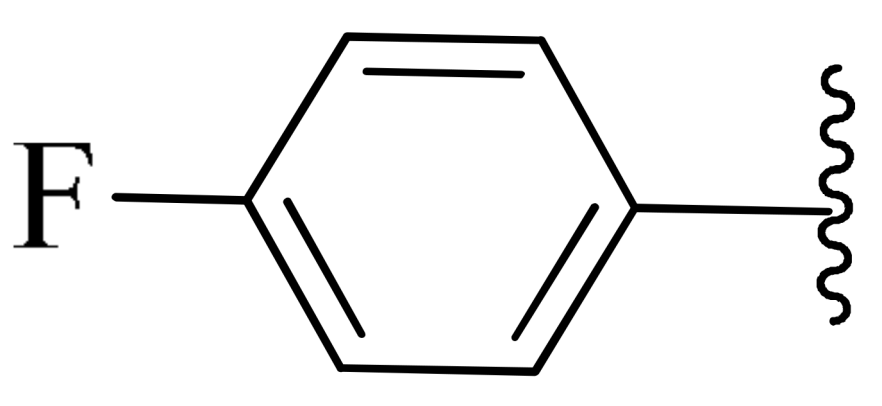	0	0	0	0	0	0	>100
**6i’**	CH_3_	CH_3_	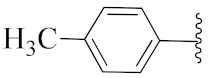	0	0	0	0	0	1	>50
**6j’**	CH_3_	CH_3_	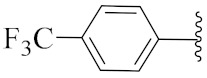	0	0	0	0	0	0	>100
**6k’**	CH_3_	CH_3_	H	1	0	0	0	0	0	>100
**6l’**	CH_3_	CH_3_	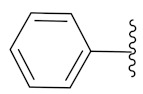	0	0	0	0	0	0	>100
**6m’**	CH_3_	CH_3_	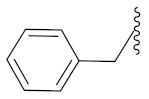	0	0	0	0	0	0	>100
**6n’**	CH_3_	CH_3_	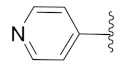	0	0	0	0	0	0	>50
**6o’**	CH_3_	CH_3_	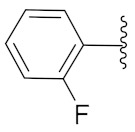	0	0	0	0	0	0	>50
**6p’**	CH_3_	CH_3_	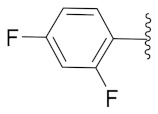	0	0	0	0	1	1	>100
**6q’**	CH_3_	CH_3_	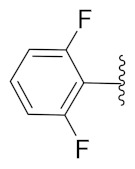	0	0	0	0	0	0	>100
**6r’**	H	CH(CH_3_)_2_	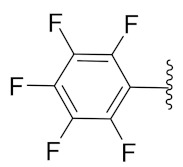	2	4	4	2	4	4	5.524
**6s’**	H	CH(CH_3_)_2_	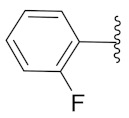	0	0	0	0	0	0	>100
**6t’**	H	CH(CH_3_)_2_	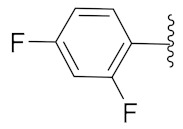	0	2	2	0	2	2	84.48
**6u’**	H	CH(CH_3_)_2_	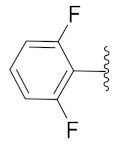	0	2	2	0	0	0	>100

^a^ Severity scores ranged from 0 (no effect) to a maximum of 4 and the descriptors underlying the scores presented are listed in supplementary data. Scores were averaged across two experiments.

## Supplementary Materials

The following are available online at https://www.mdpi.com/1424-8247/13/2/25/s1, Bioactivity data for phthalimide analogues.

## References

[1] Rollinson D., Simpson A.J.G., Rollinson D., Simpson A.J.G. (1987). The Biology of Schistosomes: From Genes to Latrines.

[2] Steinmann P., Keiser J., Bos R., Tanner M., Utzinger J. (2006). Schistosomiasis and water resources development: Systematic review, meta-analysis, and estimates of people at risk. Lancet Infect. Dis..

[3] Fenwick A. (2017). Schistosomiasis research and control since the retirement of Sir Patrick Manson in 1914. Trans. R. Soc. Trop. Med. Hyg..

[4] Bergquist R., Zhou X.N., Rollinson D., Reinhard-Rupp J., Klohe K. (2017). Elimination of schistosomiasis: The tools required. Infect. Dis Poverty.

[5] Colley D.G., Bustinduy A.L., Secor W.E., King C.H. (2014). Human schistosomiasis. Lancet.

[6] Utzinger J., N’goran E.K., Caffrey C.R., Keiser J. (2010). From innovation to application: Social-ecological context, diagnostics, drugs and integrated control of schistosomiasis. Acta Trop..

[7] McManus D.P., Dunne D.W., Sacko M., Utzinger J., Vennervald B.J., Zhou X.N. (2018). Schistosomiasis. Nat. Rev. Dis Primers.

[8] Warren K.S., Mahmoud A.A.F., Cummings P., Murphy D.J., Houser H.B. (1974). Schistosomiasis Mansoni in Yemeni in California: Duration of Infection, Presence of Disease, Therapeutic Management. Am. J. Trop. Med. Hyg..

[9] Chabasse D., Bertrand G., Leroux J.P., Gauthey N., Hocquet P. (1985). Developmental bilharziasis caused by Schistosoma mansoni discovered 37 years after infestation. Bull. Soc. Pathol. Exot. Filiales.

[10] Gryseels B. (2012). Schistosomiasis. Infect. Dis Clin. N. Am..

[11] Ross A.G.P., Bartley P.B., Sleigh A.C., Olds G.R., Li Y., Williams G.M., McManus D.P. (2002). Schistosomiasis. N. Engl. J. Med..

[12] Barsoum R.S. (2013). Urinary schistosomiasis: Review. J. Adv. Res..

[13] Deribe K., Eldaw A., Hadziabduli S., Kailie E., Omer M.D., Mohammed A.E., Jamshed T., Mohammed E.A., Mergani A., Ali G.A. (2011). High prevalence of urinary schistosomiasis in two communities in South Darfur: Implication for interventions. Parasite Vectors.

[14] Brindicci G., Santoro C.R., De Laurentiis V., Capolongo C., Solarino M.E., Papagni R., Cirac E., Gatti P., Loconsole D., Monno R. (2017). Prevalence of Urinary Schistosomiasis in Migrants in Apulia, a Region of Southern Italy, in the Years 2006–2016. BioMed. Res. Int..

[15] Munisi D. (2016). Intestinal Schistosomiasis among Primary Schoolchildren in Two On-Shore Communities in Rorya District, Northwestern Tanzania: Prevalence, Intensity of Infection and Associated Risk Factors. J. Parasitol. Res..

[16] Elbaz T., Esmat G. (2013). Hepatic and Intestinal Schistosomiasis: Review. J. Adv. Res..

[17] Teklemariam D., Legesse M., Degarege A., Liang S., Erko B. (2018). Schistosoma mansoni and other intestinal parasitic infections in schoolchildren and vervet monkeys in Lake Ziway area, Ethiopia. BMC Res. Notes.

[18] Ghiwot Y., Degarege A., Erko B. (2014). Prevalence of Intestinal Parasitic Infections among Children under Five Years of Age with Emphasis on Schistosoma mansoni in Wonji Shoa Sugar Estate, Ethiopia. PLoS ONE.

[19] Nour N.M. (2010). Schistosomiasis: Health effects on women. Rev. Obstet. Gynecol..

[20] Caffrey C.R., Secor W.E. (2011). Schistosomiasis: From drug deployment to drug development. Curr. Opin. Infect. Dis..

[21] Caffrey C.R. (2015). Schistosomiasis and its treatment. Future Med. Chem..

[22] Wolfe A.R., Neitz R.J., Burlingame M., Suzuki B.M., Lim K.C., Scheideler M., Nelson D.L., Benet L.Z., Caffrey C.R. (2018). TPT sulfonate, a single, oral dose schistosomicidal prodrug: In vivo efficacy, disposition and metabolic profiling. Int. J. Parasitol. Drugs Drug.

[23] Couto F.F., Coelho P.M., Araújo N., Kusel J.R., Katz N., Jannotti-Passos L.K., Mattos A.C. (2011). Schistosoma mansoni: A method for inducing resistance to praziquantel using infected Biomphalaria glabrata snails. Mem. Inst. Oswaldo Cruz.

[24] Fallon P.G., Doenhoff M.J. (1994). Drug-resistant schistosomiasis: Resistance to praziquantel and oxamniquine induced in Schistosoma mansoni in mice is drug specific. Am. J. Trop. Med. Hyg..

[25] Shiheido H., Terada F., Tabata N., Hayakawa I., Matsumura N., Takashima H., Ogawa Y., Du W., Yamada T., Shoji M. (2012). A Phthalimide Derivative That Inhibits Centrosomal Clustering Is Effective on Multiple Myeloma. PLoS ONE.

[26] Coêlho L.C.D., Cardoso M.V.D.O., Moreira D.R.M., Gomes P.A.T.D.M., Cavalcanti S.M.T., Oliveira A.R., Filho G.B.D.O., Siqueira L.R.P.D., Barbosa M.D.O., Borba E.F.D.O. (2014). Novel phthalimide derivatives with TNF-α and IL-1β expression inhibitory and apoptotic inducing properties. MedChemComm.

[27] Sharma U., Kumar P., Kumar N., Singh B. (2010). Recent Advances in the Chemistry of Phthalimide Analogues and their Therapeutic Potential. Mini Rev. Med. Chem..

[28] Horvat M., Uzelac L., Marjanović M., Cindro N., Franković O., Mlinarić-Majerski K., Kralj M., Basarić N. (2012). Evaluation of Antiproliferative Effect of N-(alkyladamantyl)phthalimides In vitro. Chem. Biol. Drug Des..

[29] Júnior J. (2019). Therapeutic Potential of Phthalimide Derivatives: A Review. Am. J. Biomed. Sci..

[30] Akgün H., Karamelekoğlu İ., Berk B., Kurnaz I., Sarıbıyık G., Öktem S., Kocagöz T. (2012). Synthesis and antimycobacterial activity of some phthalimide derivatives. Bioorg. Med. Chem..

[31] Alanazi A.M., El-Azab A.S., Al-Suwaidan I.A., ElTahir K.E.H., Asiri Y.A., Abdel-Aziz N.I., Abdel-Aziz A.A.M. (2015). Structure-based design of phthalimide derivatives as potential cyclooxygenase-2 (COX-2) inhibitors: Anti-inflammatory and analgesic activities. Eur. J. Med. Chem..

[32] Kushwaha N., Kaushik D. (2016). Recent Advances and Future Prospects of Phthalimide Derivatives. J. App. Pharm. Sci..

[33] Shah K., Chhabra S., Shrivastava S.K., Mishra P. (2013). Benzimidazole: A promising pharmacophore. Med. Chem. Res..

[34] Kumar P., Achieng A.O., Rajendran V., Ghosh P.C., Singh B.K., Rawat M., Perkins D.J., Kempaiah P., Rathi B. (2017). Synergistic blending of high-valued heterocycles inhibits growth of Plasmodium falciparum in culture and P. berghei infection in mouse model. Sci. Rep..

[35] Singh A.K., Rajendran V., Pant A., Ghosh P.C., Singh N., Latha N., Garg S., Pandey K.C., Singh B.K., Rathi B. (2015). Design, synthesis and biological evaluation of functionalized phthalimides: A new class of antimalarials and inhibitors of falcipain-2, a major hemoglobinase of malaria parasite. Bioorg. Med. Chem..

[36] Singh A.K., Rathore S., Tang Y., Goldfarb N.E., Dunn B.M., Rajendran V., Ghosh P.C., Singh N., Latha N., Singh B.K. (2015). Hydroxyethylamine Based Phthalimides as New Class of Plasmepsin Hits: Design, Synthesis and Antimalarial Evaluation. PLoS ONE.

[37] Boechat N., Pinheiro L.C.S., Silva T.S., Aguiar A.C.C., Carvalho A.S., Bastos M.M., Costa C.C.P., Pinheiro S., Pinto A.C., Mendonça J.S. (2012). New Trifluoromethyl Triazolopyrimidines as Anti-Plasmodium falciparum Agents. Molecules.

[38] Daina A., Michielin O., Zoete V. (2017). SwissADME: A free web tool to evaluate pharmacokinetics, drug-likeness and medicinal chemistry friendliness of small molecules. Sci. Rep..

[39] Di L., Kerns E.H., Di L., Kerns E.H. (2016). Chapter 10—Blood-Brain Barrier. Drug-Like Properties.

[40] Abdulla M.H., Ruelas D.S., Wolff B., Snedecor J., Lim K.C., Xu F., Renslo A.R., Williams J., McKerrow J.H., Caffrey C.R. (2009). Drug discovery for schistosomiasis: Hit and lead compounds identified in a library of known drugs by medium-throughput phenotypic screening. PLoS Negl. Trop. Dis..

[41] Maccesi M., Aguiar P.H.N., Pasche V., Padilla M., Suzuki B.M., Montefusco S., Abagyan R., Keiser J., Mourão M.M., Caffrey C.R. (2019). Multi-center screening of the Pathogen Box collection for schistosomiasis drug discovery. Parasite Vectors.

[42] Long T., Neitz R.J., Beasley R., Kalyanaraman C., Suzuki B.M., Jacobson M.P., Dissous C., McKerrow J.H., Drewry D.H., Zuercher W.J. (2016). Structure-Bioactivity Relationship for Benzimidazole Thiophene Inhibitors of Polo-Like Kinase 1 (PLK1), a Potential Drug Target in Schistosoma mansoni. PLoS Negl. Trop. Dis..

[43] Long T., Rojo-Arreola L., Shi D., El-Sakkary N., Jarnagin K., Rock F., Meewan M., Rascón A.A., Lin L., Cunningham K.A. (2017). Phenotypic, chemical and functional characterization of cyclic nucleotide phosphodiesterase 4 (PDE4) as a potential anthelmintic drug target. PLoS Negl. Trop. Dis..

[44] Lamie P.F., Philoppes J.N., El-Gendy A.O., Rarova L., Gruz J. (2015). Design, Synthesis and Evaluation of Novel Phthalimide Derivatives as in Vitro Anti-Microbial, Anti-Oxidant and Anti-Inflammatory Agents. Molecules.

[45] Caffrey C.R., El-Sakkary N., Mäder P., Krieg R., Becker K., Schlitzer M., Drewry D.H., Vennerstrom J.L., Grevelding C.G., Swinney D., Pollastri M., Mannhold R., Buschmann H., Holenz J. (2020). Drug Discovery and Development for Schistosomiasis. Neglected Tropical Diseases.

[46] Furniss B.S., Hannafold A.J., Smith P.W.G., Tatchell A.R. (1989). Vogel’s Textbook of Practical Organic Chemistry.

[47] Abdulla M.H., Lim K.C., Sajid M., McKerrow J.H., Caffrey C.R. (2007). Schistosomiasis mansoni: Novel chemotherapy using a cysteine protease inhibitor. PLoS Med..

[48] Stefanić S., Dvořák J., Horn M., Braschi S., Sojka D., Ruelas D.S., Suzuki B., Lim K.C., Hopkins S.D., McKerrow J.H. (2010). RNA interference in Schistosoma mansoni schistosomula: Selectivity, sensitivity and operation for larger-scale screening. PLoS Negl. Trop. Dis..

[49] Sabah A.A., Fletcher C., Webbe G., Doenhoff M.J. (1986). Schistosoma mansoni: Chemotherapy of infections of different ages. Exp. Parasitol..

[50] Caffrey C.R. (2007). Chemotherapy of schistosomiasis: Present and future. Curr. Opin. Chem. Biol..

[51] Silvie O., Rubinstein E., Franetich J.F., Prenant M., Belnoue E., Rénia L., Hannoun L., Eling W., Levy S., Boucheix C. (2003). Hepatocyte CD81 is required for Plasmodium falciparum and Plasmodium yoelii sporozoite infectivity. Nat. Med..

[52] Yalaoui S., Huby T., Franetich J.F., Gego A., Rametti A., Moreau M., Collet X., Siau A., van Gemert G.J., Sauerwein R.W. (2008). Scavenger receptor BI boosts hepatocyte permissiveness to Plasmodium infection. Cell Host Microbe.

