# Supplementary material for: Synthesis and Bioactivity of Phthalimide Analogs as Potential Drugs to Treat Schistosomiasis, a Neglected Disease of Poverty
Source: Pharmaceuticals (Basel). 2020 Feb 3;13(2):25. doi: 10.3390/ph13020025 (PMC7169845; doi:10.3390/ph13020025)
Supplement: Supplementary file 1 [file pharmaceuticals-13-00025-s001.zip › Chemistry_Suppl Data.docx]

**Synthesis and bioactivity of phthalimide analogs as potential drugs to treat schistosomiasis, a neglected disease of poverty**

Snigdha Singh^1,4^, Nelly El-Sakkary^2^, Danielle Skinner^2^, Prem P Sharma^1^, Sabine Ottilie^3^, Yevgeniya Antonova-Koch^6^, Prashant Kumar^4^, Elizabeth Winzeler^3^, Poonam^5^, Conor R. Caffrey^2,*^ and Brijesh Rathi^1,*^

^1^Laboratory for Translational Chemical Biology, Department of Chemistry, Hansraj College University Enclave, University of Delhi, Delhi-110007, India

^2^Center for Discovery and Innovation in Parasitic Diseases, Skaggs School of Pharmacy and Pharmaceutical Sciences, University of California San Diego, 9500 Gilman Drive, La Jolla, CA 92093, United States of America

^3^ Department of Pediatrics, University of California, San Diego, School of Medicine, La Jolla, CA 92093, United States

^4^Department of Chemistry, University of Delhi, Delhi-110007, India

^5^Department of Chemistry, Miranda House, University of Delhi, Delhi-110007, India

^6^Calibr – A Division of Scripps Research, 11119 N Torrey Pines Rd, La Jolla, CA 92037

**Corresponding Authors**

Conor Caffrey, Ph.D.

Email: [ccaffrey@ucsd.edu](mailto:ccaffrey@ucsd.edu)

Brijesh Rathi, PhD

Email: [brijeshrathi@hrc.du.ac.in](mailto:brijeshrathi@hrc.du.ac.in)

**Table S1:** Predicted ADME profiles of Pht analogues **6**(**a-u’**)

| **Code** | **MW** | **Rotatable bonds** | **H-bond acceptors** | **H-bond donors** | **TPSA** | **XLOGP3** | **MLOGP** | **ESOL Log S** | **ESOL Class** | **GI absorption** | **BBB permeant** | **Lipinski violations** | **Bioavailabilty Score** |
| --- | --- | --- | --- | --- | --- | --- | --- | --- | --- | --- | --- | --- | --- |
| 6a | 522.57 | 7 | 6 | 0 | 85.91 | 5.19 | 4.3 | -6.38 | Poorly soluble | High | No | 2 | 0.17 |
| 6b | 518.61 | 7 | 5 | 0 | 85.91 | 5.45 | 4.39 | -6.52 | Poorly soluble | High | No | 2 | 0.17 |
| 6y | 466.47 | 5 | 6 | 0 | 85.91 | 3.5 | 3.52 | -5.16 | Moderately soluble | High | No | 0 | 0.55 |
| 6c | 428.49 | 6 | 5 | 1 | 96.77 | 3.41 | 3.21 | -4.71 | Moderately soluble | High | No | 0 | 0.55 |
| 6z | 462.5 | 5 | 5 | 0 | 85.91 | 3.77 | 3.62 | -5.3 | Moderately soluble | High | No | 0 | 0.55 |
| 6g’ | 372.38 | 4 | 5 | 1 | 96.77 | 1.73 | 2.34 | -3.5 | Soluble | High | No | 0 | 0.55 |
| 6l | 580.51 | 7 | 10 | 0 | 85.91 | 5.23 | 5.56 | -6.73 | Poorly soluble | Low | No | 2 | 0.17 |
| 6m | 558.55 | 8 | 8 | 0 | 85.91 | 5.61 | 4.49 | -6.78 | Poorly soluble | Low | No | 2 | 0.17 |
| 6e | 572.58 | 8 | 8 | 0 | 85.91 | 5.97 | 4.67 | -7.08 | Poorly soluble | Low | No | 2 | 0.17 |
| 6d | 594.53 | 7 | 10 | 0 | 85.91 | 5.59 | 5.75 | -7.03 | Poorly soluble | Low | No | 2 | 0.17 |
| 6t | 580.51 | 7 | 10 | 0 | 85.91 | 5.23 | 5.56 | -6.73 | Poorly soluble | Low | No | 2 | 0.17 |
| 6h’ | 480.49 | 5 | 6 | 0 | 85.91 | 3.87 | 3.72 | -5.46 | Moderately soluble | High | No | 0 | 0.55 |
| 6i’ | 476.53 | 5 | 5 | 0 | 85.91 | 4.13 | 3.81 | -5.6 | Moderately soluble | High | No | 0 | 0.55 |
| 6j’ | 530.5 | 6 | 8 | 0 | 85.91 | 4.65 | 4.11 | -6.16 | Poorly soluble | High | No | 1 | 0.55 |
| 6k’ | 386.41 | 4 | 5 | 1 | 96.77 | 2.09 | 2.56 | -3.8 | Soluble | High | No | 0 | 0.55 |
| 6l’ | 462.5 | 5 | 5 | 0 | 85.91 | 3.77 | 3.62 | -5.3 | Moderately soluble | High | No | 0 | 0.55 |
| 6m’ | 476.53 | 6 | 5 | 0 | 85.91 | 3.7 | 3.81 | -5.26 | Moderately soluble | High | No | 0 | 0.55 |
| 6n’ | 463.49 | 5 | 6 | 0 | 98.8 | 2.7 | 2.64 | -4.63 | Moderately soluble | High | No | 0 | 0.55 |
| 6a’ | 448.48 | 5 | 5 | 0 | 85.91 | 3.4 | 3.41 | -5 | Moderately soluble | High | No | 0 | 0.55 |
| 6e’ | 462.5 | 6 | 5 | 0 | 85.91 | 3.34 | 3.62 | -4.97 | Moderately soluble | High | No | 0 | 0.55 |
| 6u | 491.54 | 7 | 6 | 0 | 98.8 | 3.65 | 3.04 | -5.25 | Moderately soluble | High | No | 0 | 0.55 |
| 6r’ | 566.48 | 6 | 10 | 0 | 85.91 | 4.87 | 5.37 | -6.49 | Poorly soluble | Low | No | 2 | 0.17 |
| 6b’ | 538.43 | 5 | 10 | 0 | 85.91 | 3.91 | 4.98 | -5.8 | Moderately soluble | High | No | 2 | 0.17 |
| 6f | 518.61 | 8 | 5 | 0 | 85.91 | 5.03 | 4.39 | -6.19 | Poorly soluble | High | No | 2 | 0.17 |
| 6g | 504.58 | 7 | 5 | 0 | 85.91 | 5.09 | 4.2 | -6.22 | Poorly soluble | High | No | 2 | 0.17 |
| 6h | 534.61 | 8 | 6 | 0 | 95.14 | 5.06 | 3.61 | -6.3 | Poorly soluble | High | No | 1 | 0.55 |
| 6n | 508.55 | 7 | 6 | 0 | 85.91 | 4.82 | 4.11 | -6.07 | Poorly soluble | High | No | 1 | 0.55 |
| 6o | 526.54 | 7 | 7 | 0 | 85.91 | 4.93 | 4.47 | -6.24 | Poorly soluble | High | No | 2 | 0.17 |
| 6v | 508.55 | 7 | 6 | 0 | 85.91 | 4.82 | 4.11 | -6.07 | Poorly soluble | High | No | 1 | 0.55 |
| 6w | 526.54 | 7 | 7 | 0 | 85.91 | 4.93 | 4.47 | -6.24 | Poorly soluble | High | No | 2 | 0.17 |
| 6s’ | 494.52 | 6 | 6 | 0 | 85.91 | 4.47 | 3.91 | -5.85 | Moderately soluble | High | No | 0 | 0.55 |
| 6t’ | 512.51 | 6 | 7 | 0 | 85.91 | 4.57 | 4.28 | -6.01 | Poorly soluble | High | No | 2 | 0.17 |
| 6i | 522.57 | 7 | 6 | 0 | 85.91 | 5.19 | 4.3 | -6.38 | Poorly soluble | High | No | 2 | 0.17 |
| 6j | 540.56 | 7 | 7 | 0 | 85.91 | 5.29 | 4.66 | -6.54 | Poorly soluble | High | No | 2 | 0.17 |
| 6o’ | 480.49 | 5 | 6 | 0 | 85.91 | 3.87 | 3.72 | -5.46 | Moderately soluble | High | No | 0 | 0.55 |
| 6p’ | 498.48 | 5 | 7 | 0 | 85.91 | 3.97 | 4.08 | -5.62 | Moderately soluble | High | No | 0 | 0.55 |
| 6c’ | 466.47 | 5 | 6 | 0 | 85.91 | 3.5 | 3.52 | -5.16 | Moderately soluble | High | No | 0 | 0.55 |
| 6d’ | 484.46 | 5 | 7 | 0 | 85.91 | 3.6 | 3.88 | -5.32 | Moderately soluble | High | No | 0 | 0.55 |
| 6q | 522.57 | 7 | 6 | 0 | 85.91 | 5.19 | 4.3 | -6.38 | Poorly soluble | High | No | 2 | 0.17 |
| 6p | 526.54 | 7 | 7 | 0 | 85.91 | 4.93 | 4.47 | -6.24 | Poorly soluble | High | No | 2 | 0.17 |
| 6x | 526.54 | 7 | 7 | 0 | 85.91 | 4.93 | 4.47 | -6.24 | Poorly soluble | High | No | 2 | 0.17 |
| 6u’ | 512.51 | 6 | 7 | 0 | 85.91 | 4.57 | 4.28 | -6.01 | Poorly soluble | High | No | 2 | 0.17 |
| 6k | 540.56 | 7 | 7 | 0 | 85.91 | 5.29 | 4.66 | -6.54 | Poorly soluble | High | No | 2 | 0.17 |
| 6q’ | 498.48 | 5 | 7 | 0 | 85.91 | 3.97 | 4.08 | -5.62 | Moderately soluble | High | No | 0 | 0.55 |
| 6f’ | 484.46 | 5 | 7 | 0 | 85.91 | 3.6 | 3.88 | -5.32 | Moderately soluble | High | No | 0 | 0.55 |
| 6r | 540.56 | 7 | 7 | 0 | 85.91 | 5.29 | 4.66 | -6.54 | Poorly soluble | High | No | 2 | 0.17 |
| 6s | 594.53 | 7 | 10 | 0 | 85.91 | 5.59 | 5.75 | -7.03 | Poorly soluble | Low | No | 2 | 0.17 |

**Spectroscopic data (^1^H, ^13^C, Mass)**

**Figure S1:**^1^H NMR spectrum of compound **6a** (CDCl_3_, 400MHz)

**Figure S2:**^13^C NMR spectrum of compound **6a** (CDCl_3_, 100MHz)


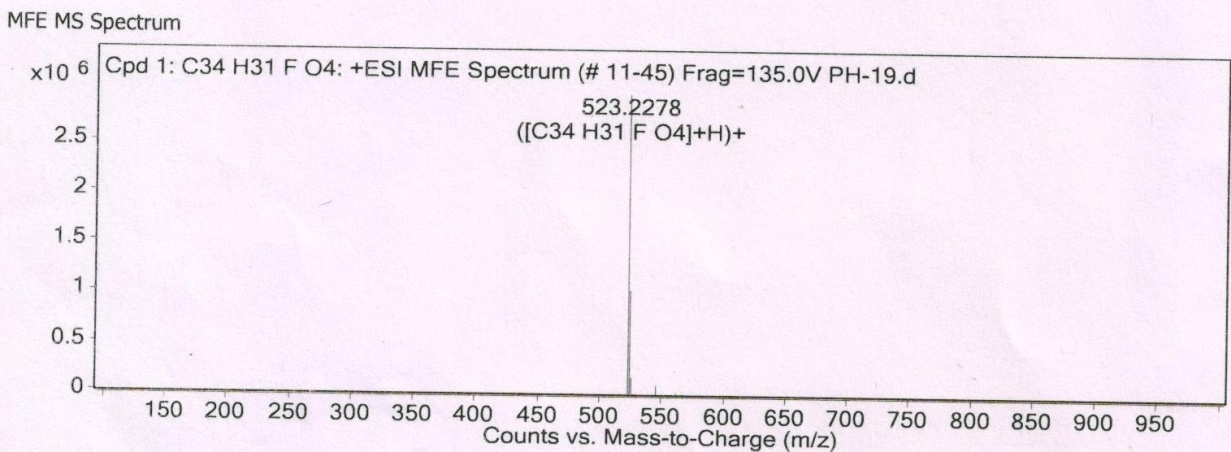


**Figure S3:**HRMS spectrum of compound **6a**

**Figure S4:**^1^H NMR spectrum of compound **6b** (CDCl_3_, 400MHz)

**Figure S5:**^13^C NMR spectrum of compound **6b** (CDCl_3_, 100MHz)


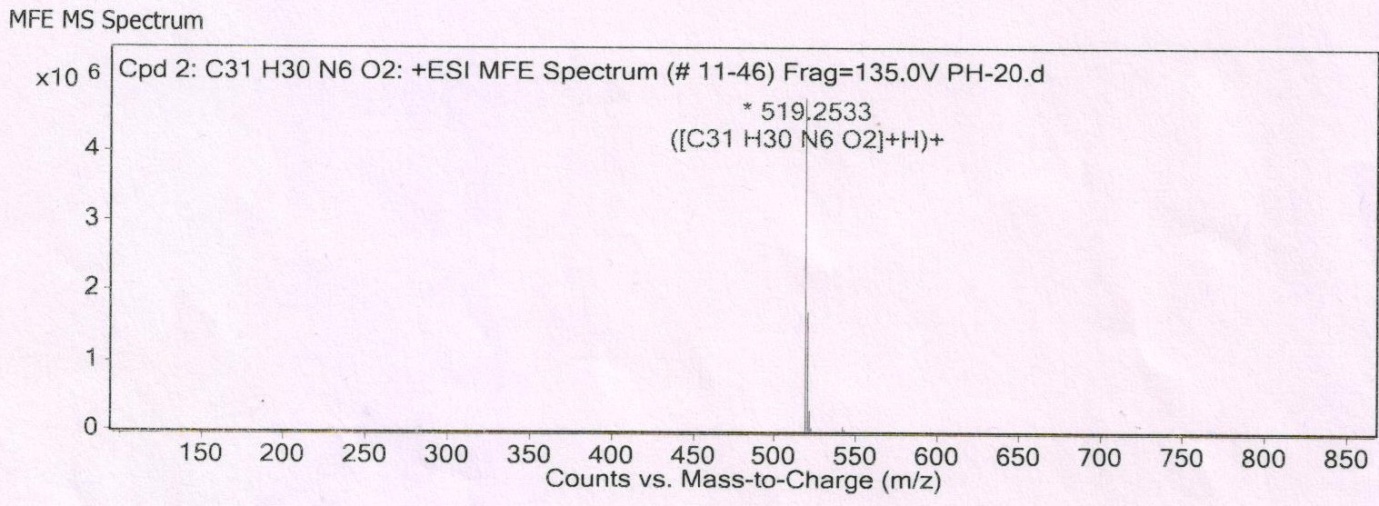
**Figure S6:** HRMS spectrum of compound **6b****Figure S7:**^1^H NMR spectrum of compound **6c** (CDCl_3_, 400MHz)

**Figure S8:**^13^C NMR spectrum of compound **6c** (CDCl_3_, 100MHz)


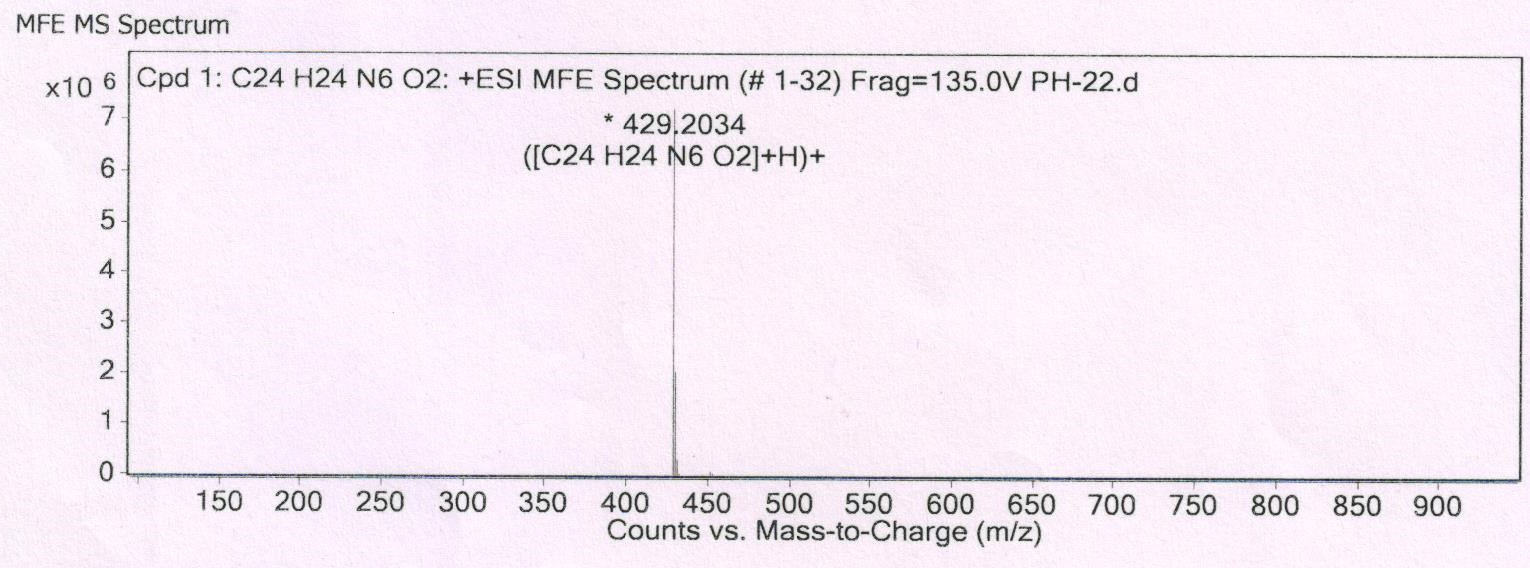


**Figure S9:**HRMS spectrum of compound **6c**

**Figure S10:**^1^H NMR spectrum of compound **6d** (CDCl_3_, 400MHz)

**Figure S11:**^13^C NMR spectrum of compound **6d** (CDCl_3_, 100MHz)


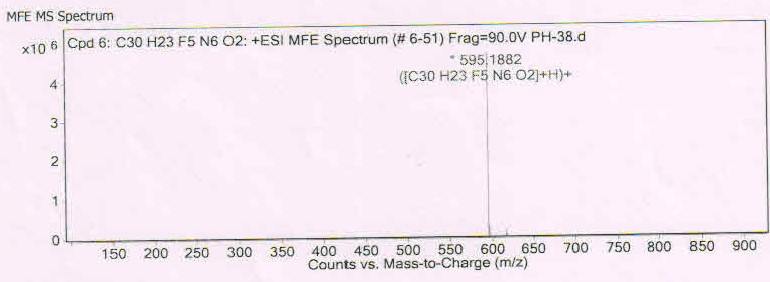


**Figure S12:**HRMS spectrum of compound **6d**

**Figure S13:**^1^H NMR spectrum of compound **6e** (CDCl_3_, 400MHz)

**Figure S14:**^13^C NMR spectrum of compound **6e** (CDCl_3_, 100MHz)


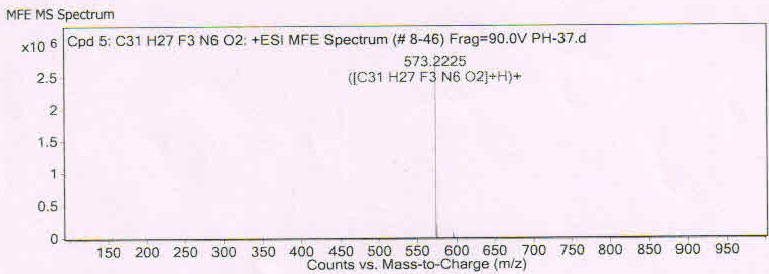


**Figure S15:**HRMS spectrum of compound **6e**

**Figure S16:**^1^H NMR spectrum of compound **6f** (CDCl_3_, 400MHz)

**Figure S17:**^13^C NMR spectrum of compound **6f** (CDCl_3_, 100MHz)


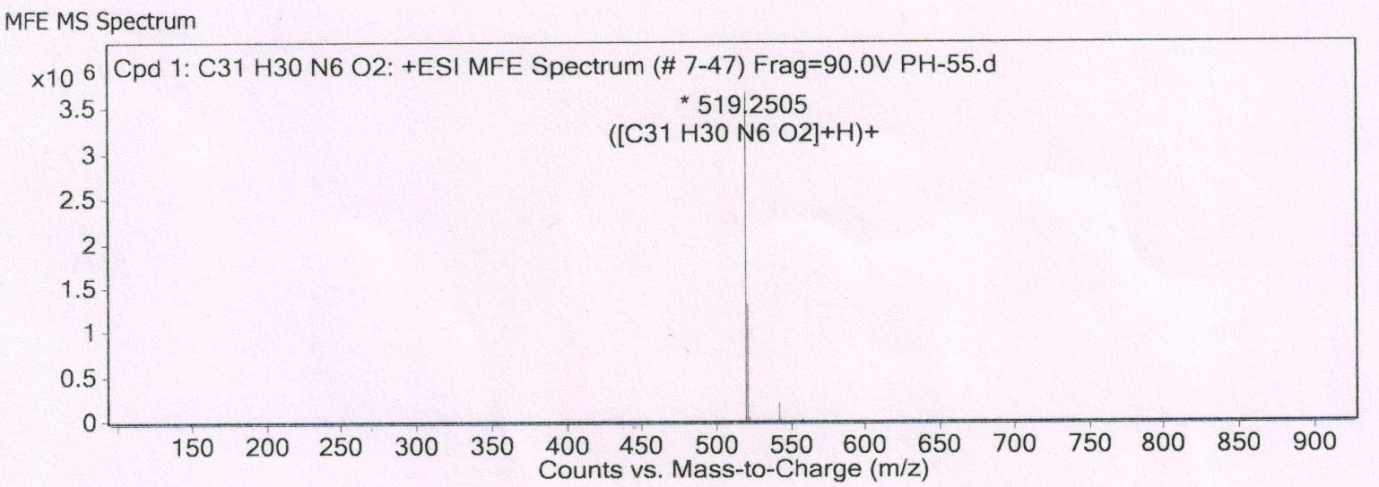


**Figure S18:**HRMS spectrum of compound **6f**

**Figure S19:**^1^H NMR spectrum of compound **6g** (CDCl_3_, 400MHz)

**Figure S20:**^13^C NMR spectrum of compound **6g** (CDCl_3_, 100MHz)


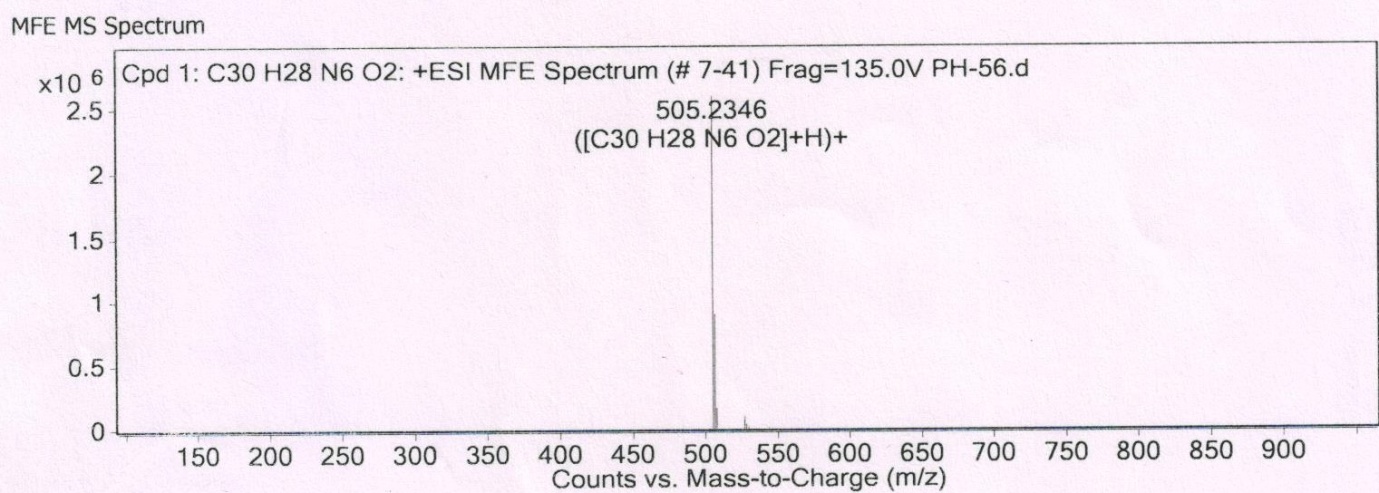


**Figure S21:**HRMS spectrum of compound **6g**

**Figure S22:**^1^H NMR spectrum of compound **6h** (CDCl_3_, 400MHz)

**Figure S23:**^13^C NMR spectrum of compound **6h** (CDCl_3_, 100MHz)


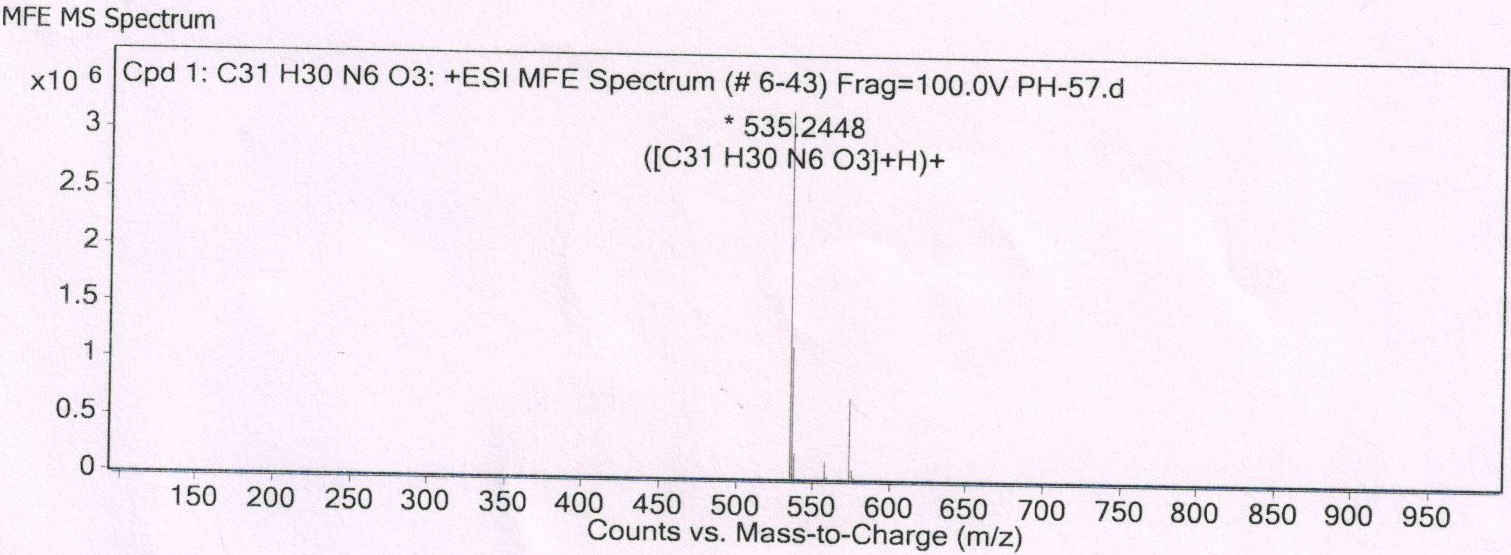


**Figure S24:**HRMS spectrum of compound **6h**

**Figure S25:**^1^H NMR spectrum of compound **6i** (CDCl_3_, 400MHz)

**Figure S26:**^13^C NMR spectrum of compound **6i** (CDCl_3_, 100MHz)


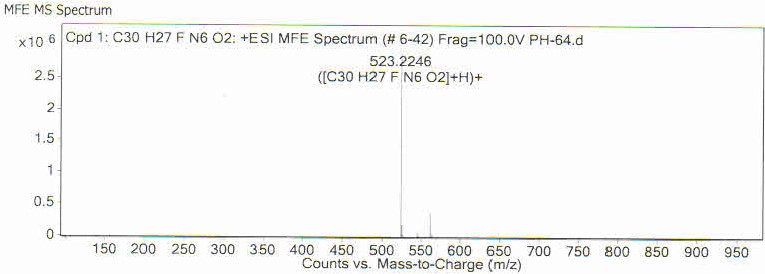


**Figure S27:**HRMS spectrum of compound **6i**

**Figure S28:**^1^H NMR spectrum of compound **6j** (CDCl_3_, 400MHz)

**Figure S29:**^13^C NMR spectrum of compound **6j** (CDCl_3_, 100MHz)

**Figure S30:**^1^H NMR spectrum of compound **6k** (CDCl_3_, 400MHz)

**Figure S31:**^13^C NMR spectrum of compound **6k** (CDCl_3_, 100MHz)


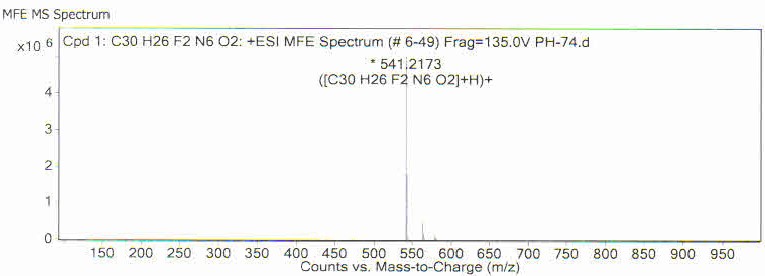


**Figure S32:**HRMS spectrum of compound **6k**

**Figure S33:**^1^H NMR spectrum of compound **6l** (CDCl_3_, 400MHz)

**Figure S34:**^13^C NMR spectrum of compound **6l** (CDCl_3_, 100MHz)


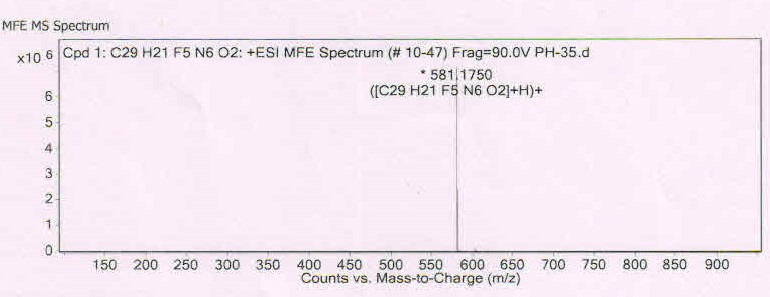


**Figure S35:**HRMS spectrum of compound **6l**

**Figure S36:**^1^H NMR spectrum of compound **6m** (CDCl_3_, 400MHz)

**Figure S37:**^13^C NMR spectrum of compound **6m** (CDCl_3_, 100MHz)


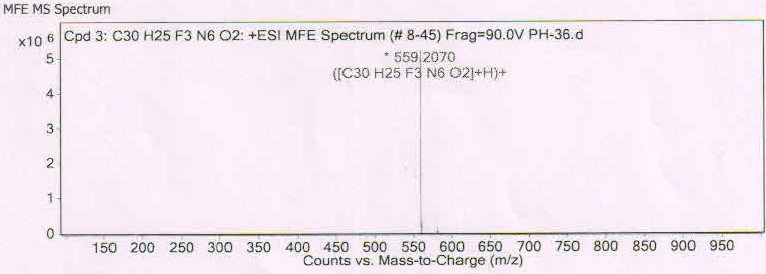


**Figure S38:**HRMS spectrum of compound **6m**

**Figure S39:**^1^H NMR spectrum of compound **6n** (CDCl_3_, 400MHz)

**Figure S40:**^13^C NMR spectrum of compound **6n** (CDCl_3_, 100MHz)


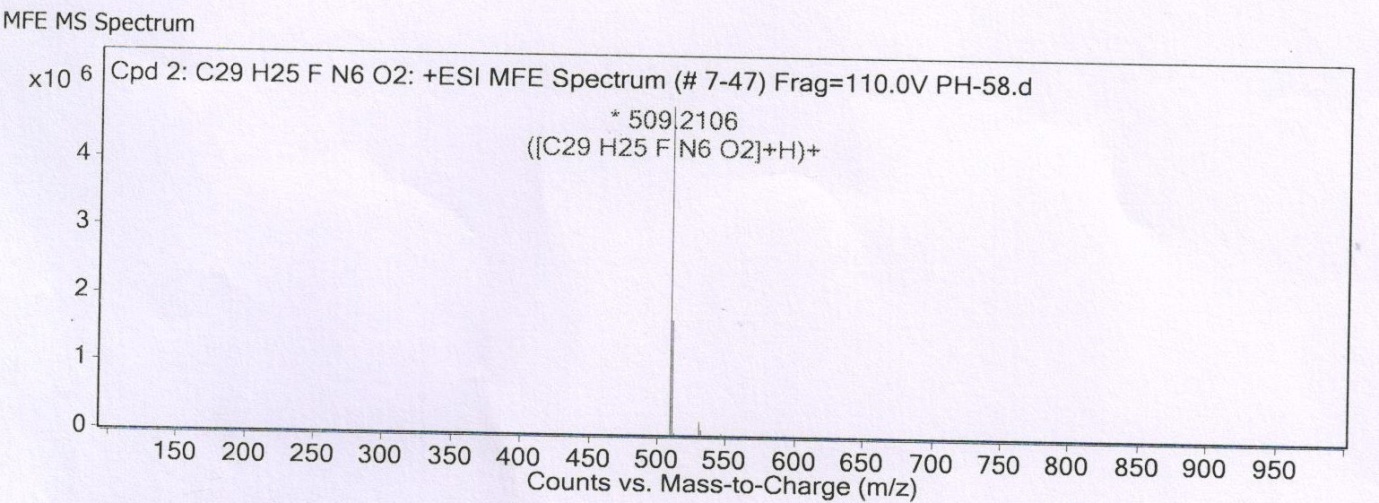


**Figure S41:**HRMS spectrum of compound **6n**

**Figure S42:**^1^H NMR spectrum of compound **6o** (CDCl_3_, 400MHz)

**Figure S43:**^13^C NMR spectrum of compound **6o** (CDCl_3_, 100MHz)


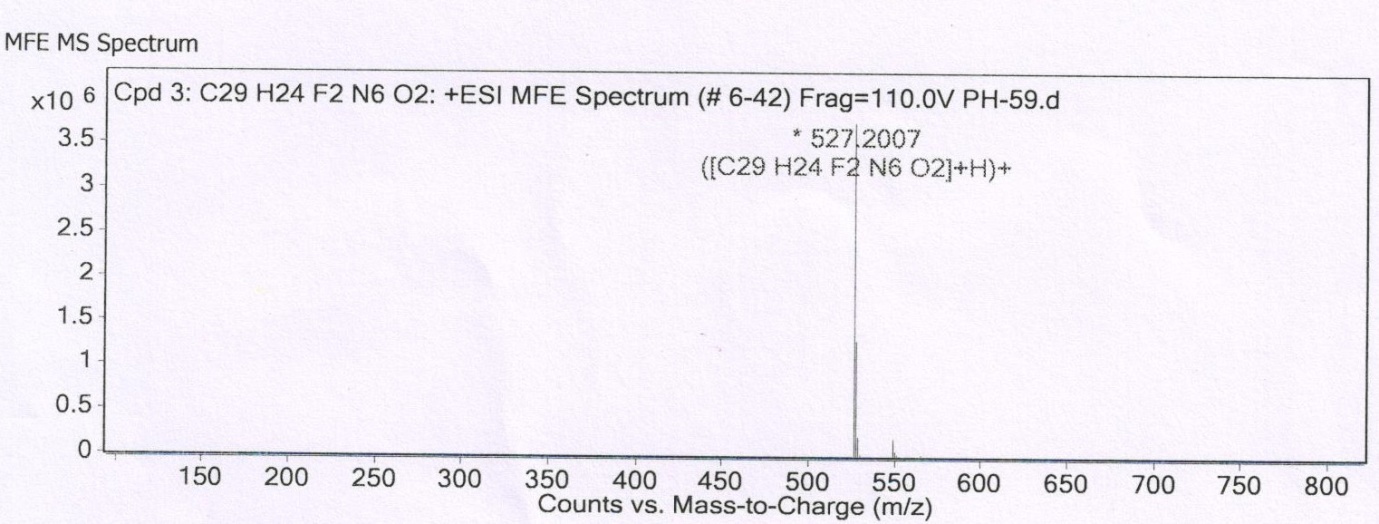


**Figure S44:**HRMS spectrum of compound **6o**

**Figure S45:**^1^H NMR spectrum of compound **6p** (CDCl_3_, 400MHz)

**Figure S46:**^13^C NMR spectrum of compound **6p** (CDCl_3_, 100MHz)


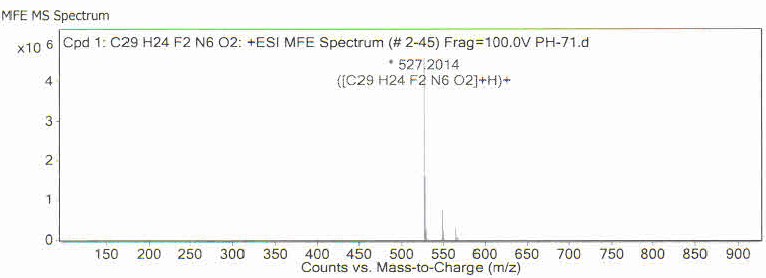


**Figure S47:**HRMS spectrum of compound **6p**

**Figure S48:**^1^H NMR spectrum of compound **6q** (CDCl_3_, 400MHz)

**Figure S49:**^13^C NMR spectrum of compound **6q** (CDCl_3_, 100MHz)


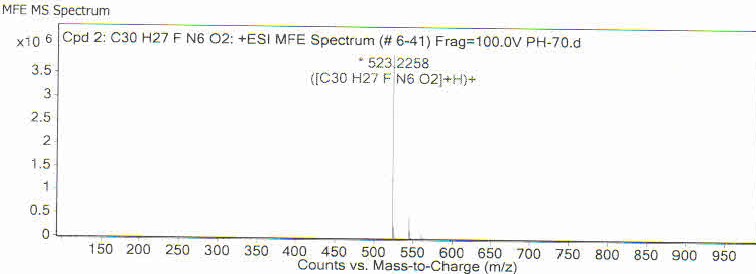


**Figure S50:** HRMS spectrum of compound **6q**

**Figure S51:**^1^H NMR spectrum of compound **6r** (CDCl_3_, 400MHz)

**Figure S52:**^13^C NMR spectrum of compound **6r** (CDCl_3_, 100MHz)


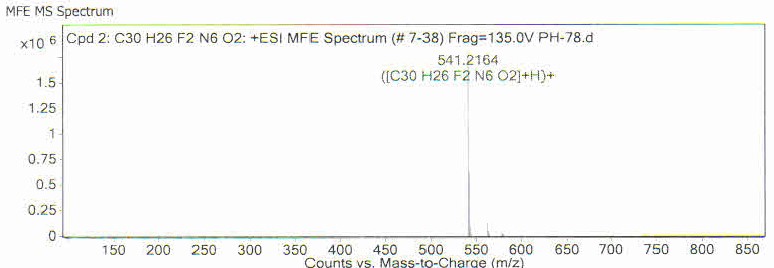


**Figure S53:**HRMS spectrum of compound **6r**

**Figure S54:**^1^H NMR spectrum of compound **6s** (CDCl_3_, 400MHz)

**Figure S55:**^13^C NMR spectrum of compound **6s** (CDCl_3_, 100MHz)

**Figure S56:**^1^H NMR spectrum of compound **6t** (CDCl_3_, 400MHz)

**Figure S57:**^13^C NMR spectrum of compound **6t** (CDCl_3_, 100MHz)


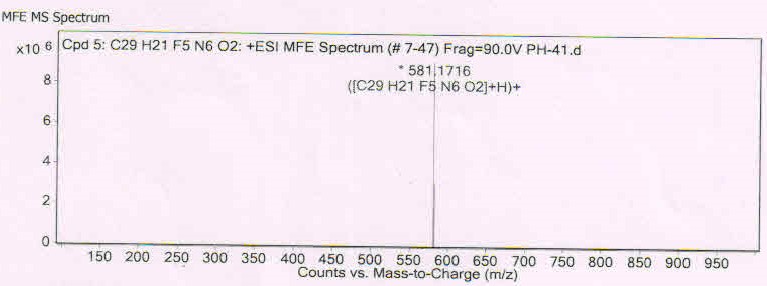


**Figure S58:**HRMS spectrum of compound **6t**

**Figure S59:**^1^H NMR spectrum of compound **6u** (CDCl_3_, 400MHz)

**Figure S60:**^13^C NMR spectrum of compound **6u** (CDCl_3_, 100MHz)


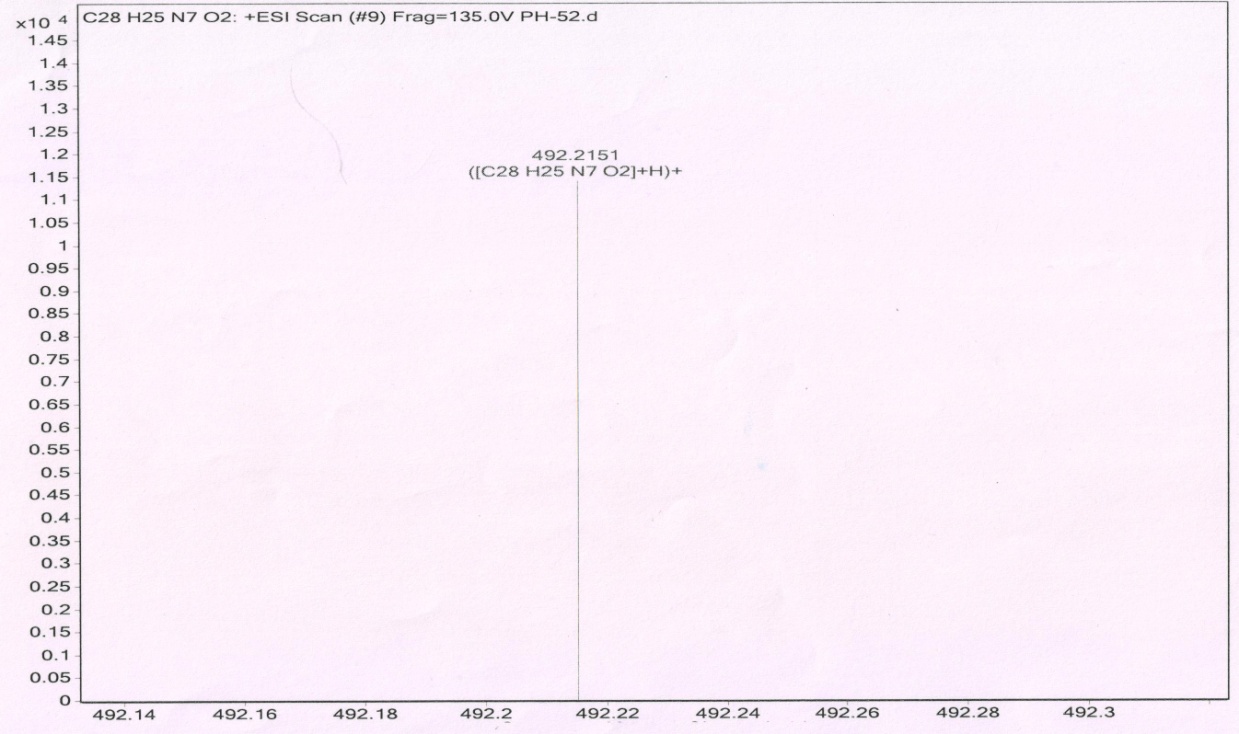


**Figure S61:**HRMS spectrum of compound **6u**

**Figure S62:**^1^H NMR spectrum of compound **6v** (CDCl_3_, 400MHz)

**Figure S63:**^13^C NMR spectrum of compound **6v** (CDCl_3_, 100MHz)


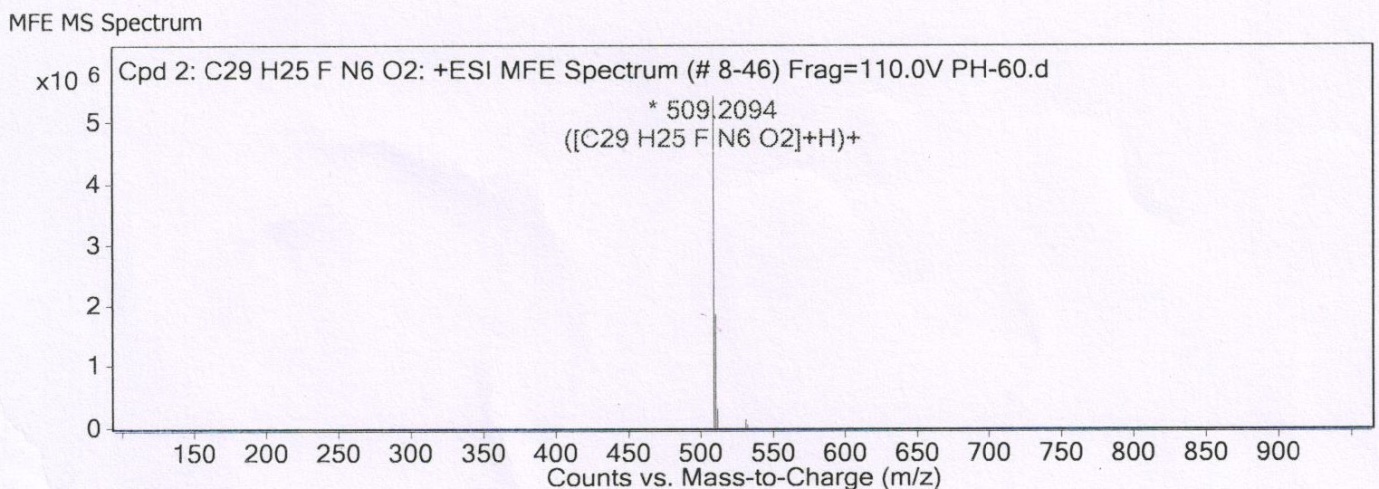


**Figure S64:**HRMS spectrum of compound **6v**

**Figure S65:**^1^H NMR spectrum of compound **6w** (CDCl_3_, 400MHz)

**Figure S66:**^13^C NMR spectrum of compound **6w** (CDCl_3_, 100MHz)


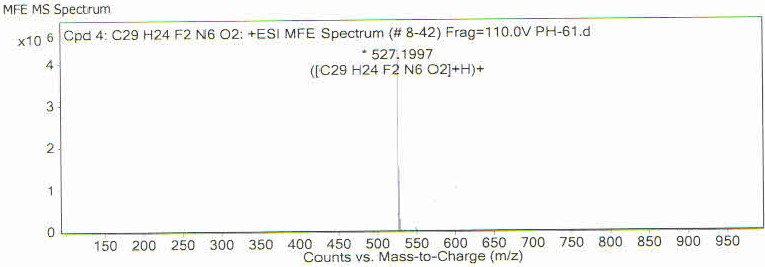


**Figure S67:**HRMS spectrum of compound **6w**

**Figure S68:**^1^H NMR spectrum of compound **6x** (CDCl_3_, 400MHz)

**Figure S69:**^13^C NMR spectrum of compound **6x** (CDCl_3_, 100MHz)


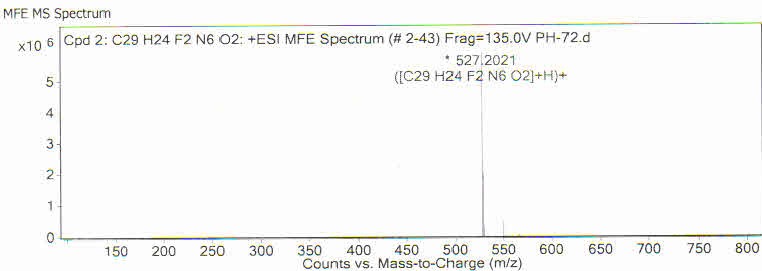


**Figure S70:**HRMS spectrum of compound **6x**

**Figure S71:**^1^H NMR spectrum of compound **6y** (CDCl_3_, 400MHz)

**Figure S72:**^13^C NMR spectrum of compound **6y** (CDCl_3_, 100MHz)


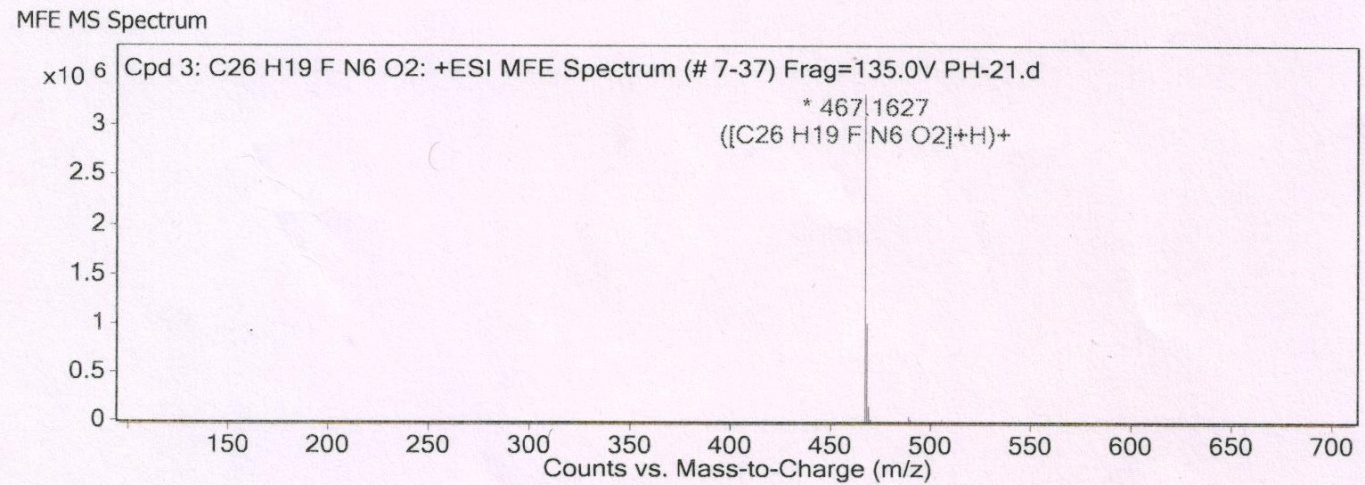


**Figure S73:**HRMS spectrum of compound **6y**

**Figure S74:**^1^H NMR spectrum of compound **6z** (CDCl_3_, 400MHz)

**Figure S75:**^13^C NMR spectrum of compound **6z** (CDCl_3_, 100MHz)


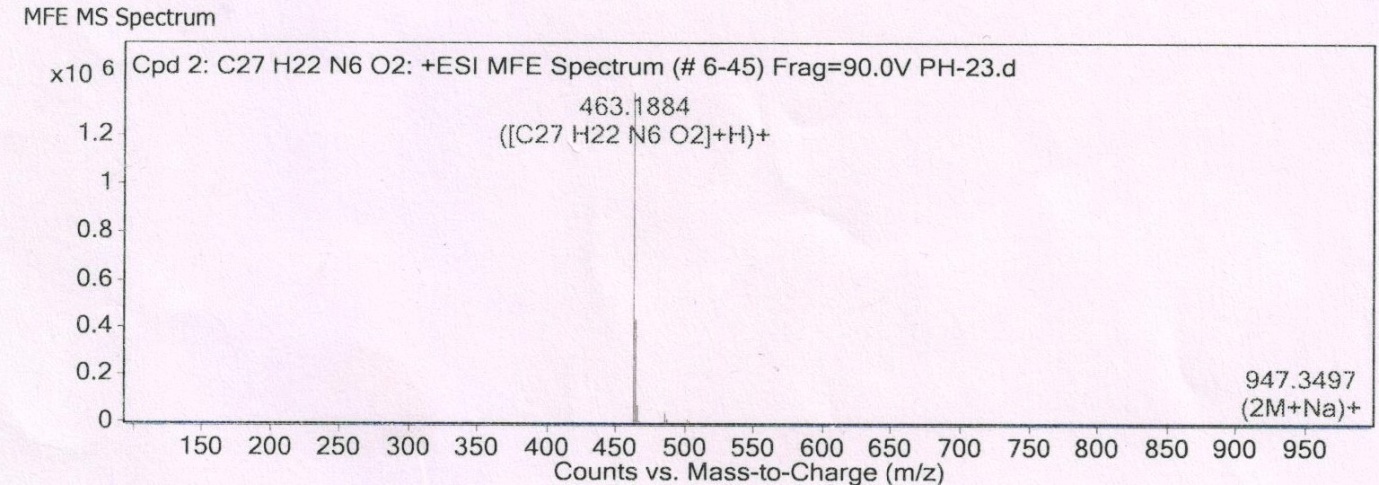


**Figure S76:**HRMS spectrum of compound **6z**

**Figure S77:**^1^H NMR spectrum of compound **6a’** (CDCl_3_, 400MHz)

**Figure S78:**^13^C NMR spectrum of compound **6a’** (CDCl_3_, 100MHz)


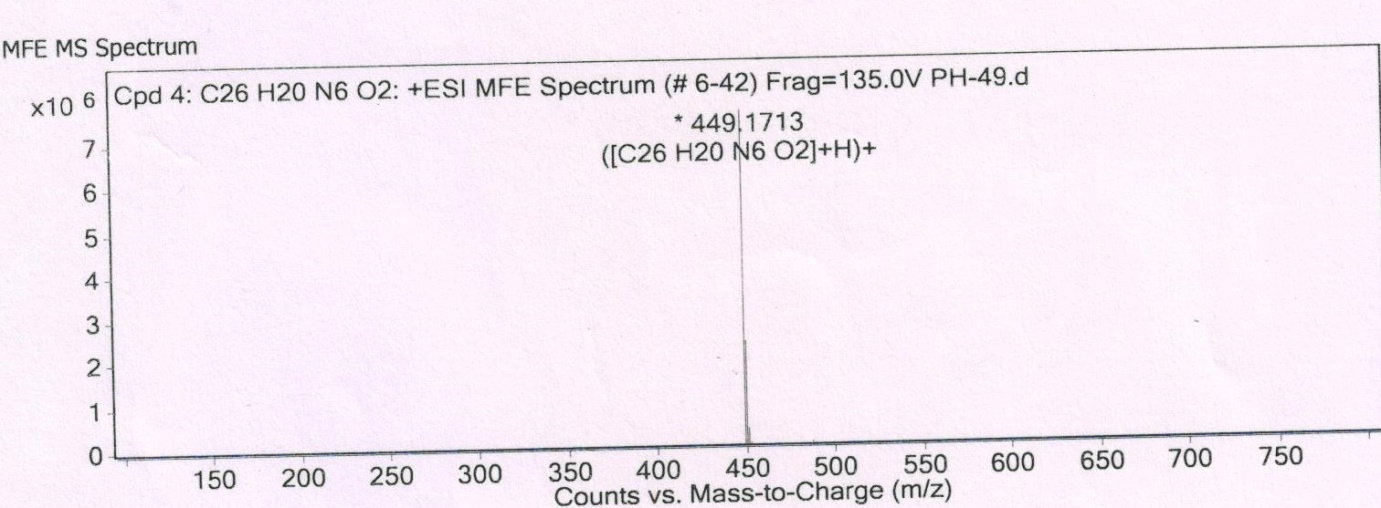


**Figure S79:**HRMS spectrum of compound **6a’**

**Figure S80:**^1^H NMR spectrum of compound **6b’** (CDCl_3_, 400MHz)

**Figure S81:**^13^C NMR spectrum of compound **6b’** (CDCl_3_, 100MHz)


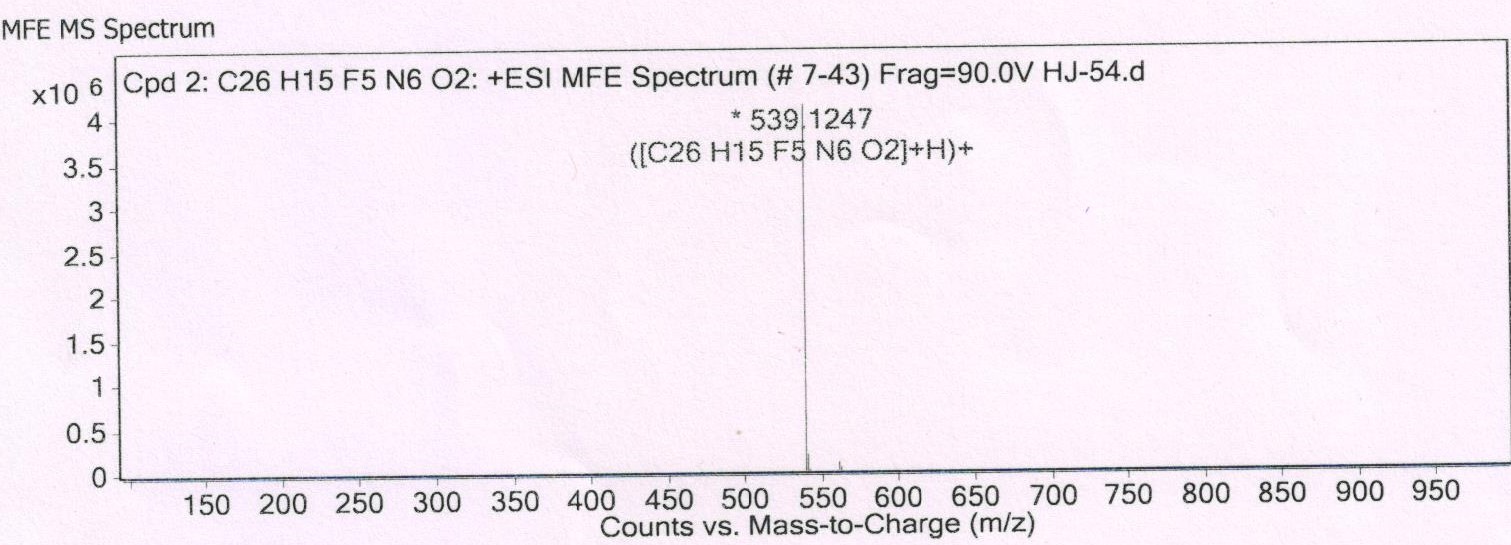


**Figure S82:**HRMS spectrum of compound **6b’**

**Figure S83:**^1^H NMR spectrum of compound **6c’** (CDCl_3_, 400MHz)

**Figure S84:**^13^C NMR spectrum of compound **6c’** (CDCl_3_, 100MHz)


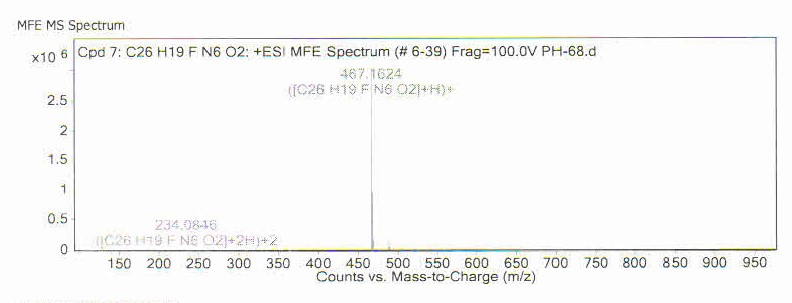


**Figure S85:**HRMS spectrum of compound **6c’**

**Figure S86:**^1^H NMR spectrum of compound **6d’** (CDCl_3_, 400MHz)

**Figure S87:**^13^C NMR spectrum of compound **6d’** (CDCl_3_, 100MHz)


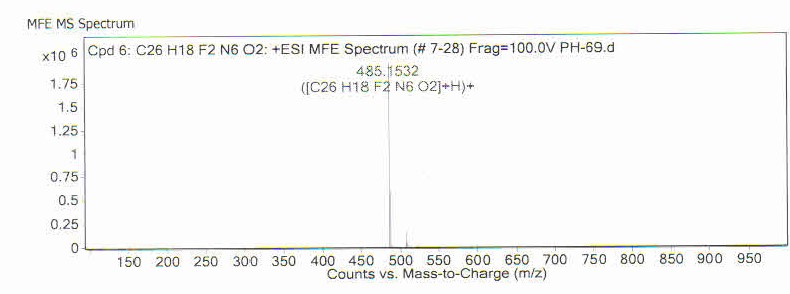


**Figure S88:**HRMS spectrum of compound **6d’**

**Figure S89:**^1^H NMR spectrum of compound **6e’** (CDCl_3_, 400MHz)

**Figure S90:**^13^C NMR spectrum of compound **6e’** (CDCl_3_, 100MHz)


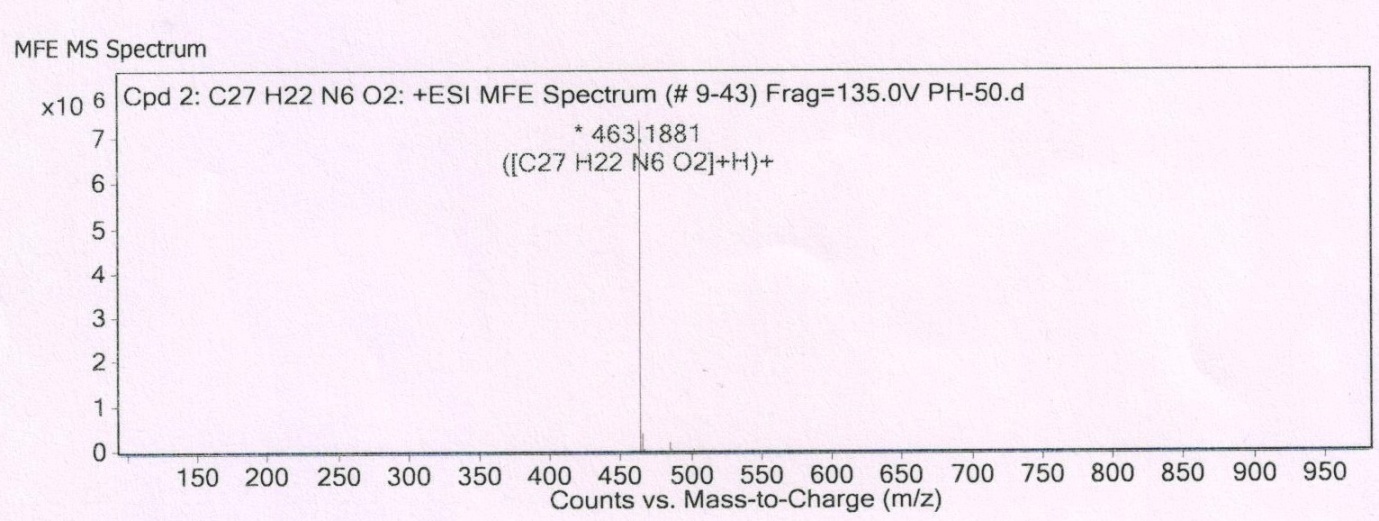


**Figure S91:**HRMS spectrum of compound **6e’**

**Figure S92:**^1^H NMR spectrum of compound **6f’** (CDCl_3_, 400MHz)

**Figure S93:**^13^C NMR spectrum of compound **6f’** (CDCl_3_, 100MHz)


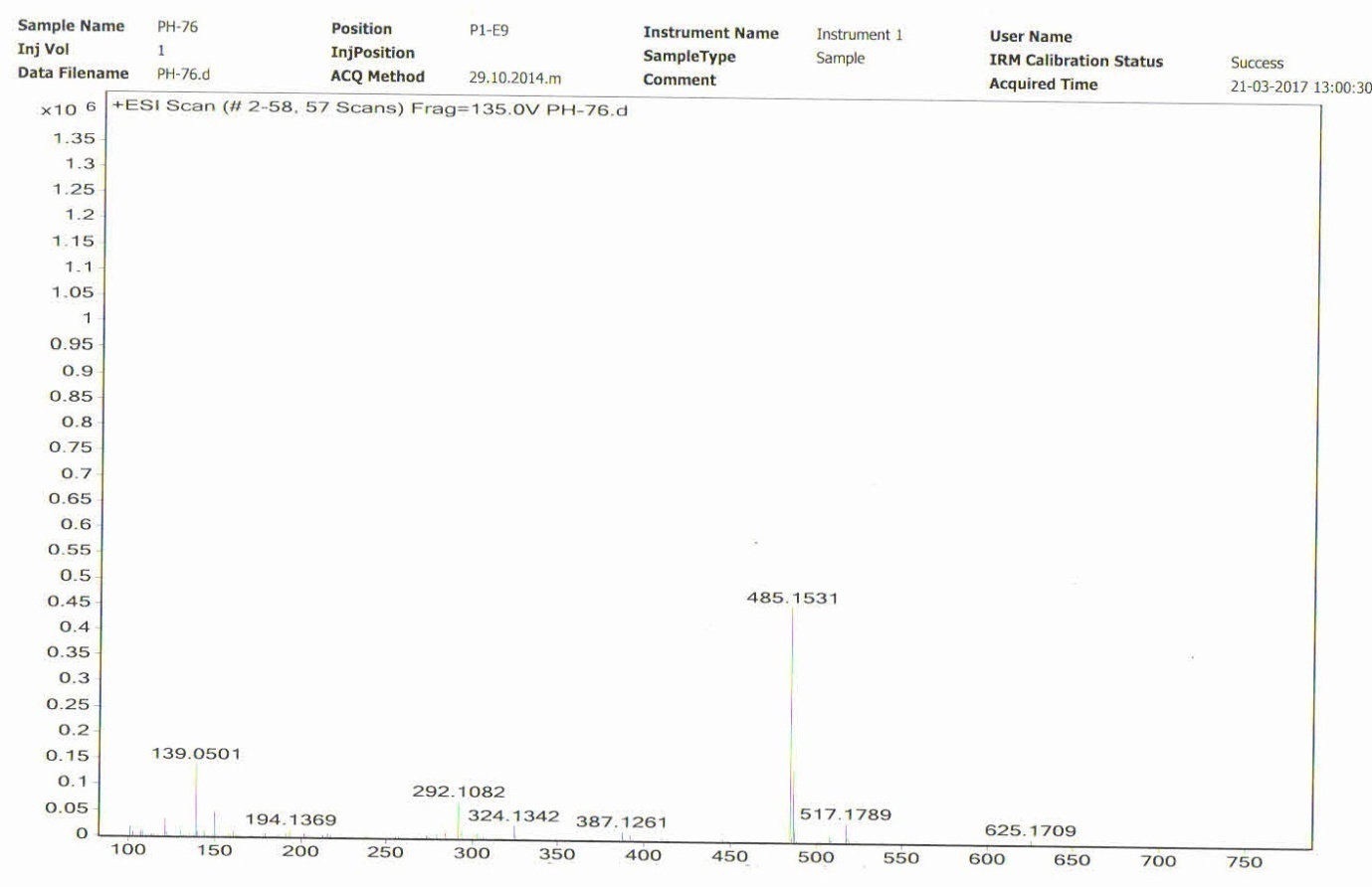


**Figure S94:**HRMS spectrum of compound **6f’**

**Figure S95:**^1^H NMR spectrum of compound **6g’** (CDCl_3_, 400MHz)

**Figure S96:**^13^C NMR spectrum of compound **6g’** (CDCl_3_, 100MHz)


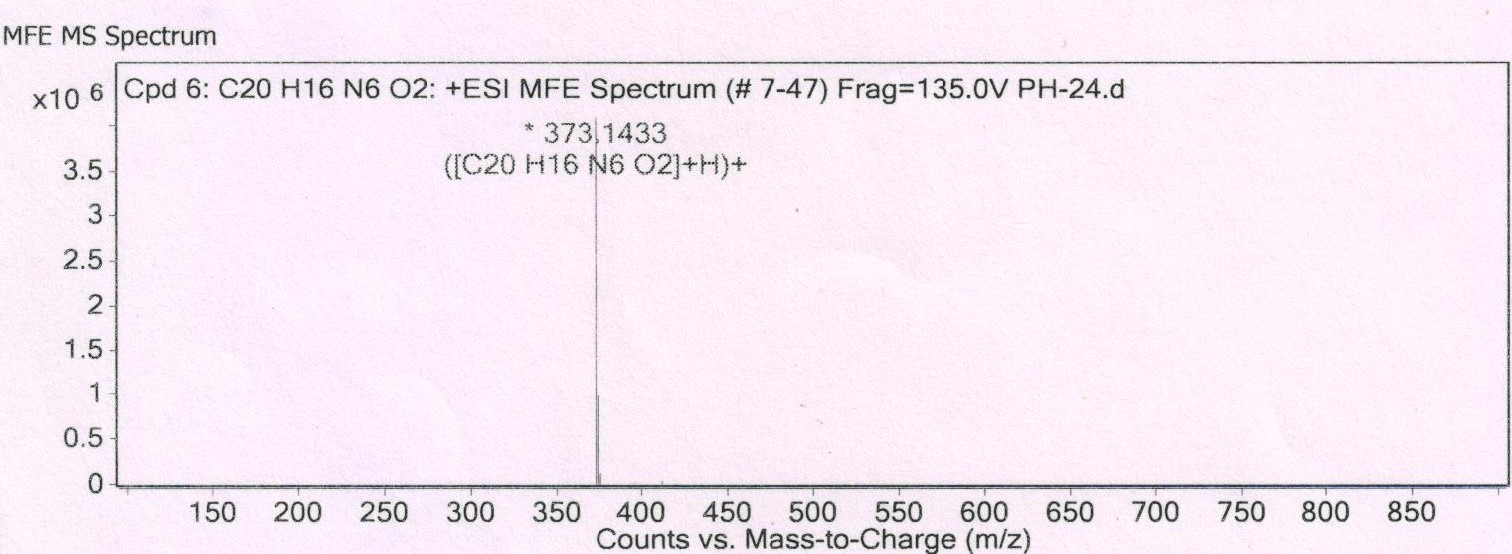


**Figure S97:**HRMS spectrum of compound **6g’**

**Figure S98:**^1^H NMR spectrum of compound **6h’** (CDCl_3_, 400MHz)

**Figure S99:**^13^C NMR spectrum of compound **6h’** (CDCl_3_, 100MHz)


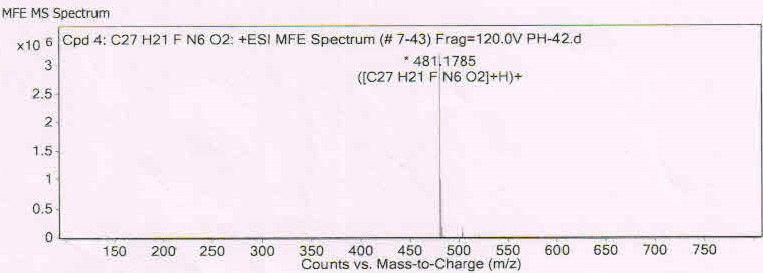


**Figure S100:**HRMS spectrum of compound **6h’**

**Figure S101:**^1^H NMR spectrum of compound **6i’** (CDCl_3_, 400MHz)

**Figure S102:**^13^C NMR spectrum of compound **6i’** (CDCl_3_, 100MHz)


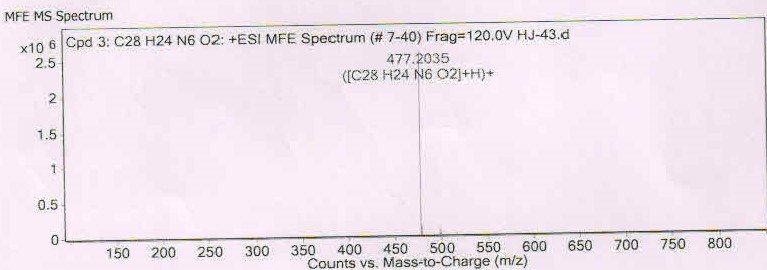


**Figure S103:**HRMS spectrum of compound **6i’**

**Figure S104:**^1^H NMR spectrum of compound **6j’** (CDCl_3_, 400MHz)

**Figure S105:**^13^C NMR spectrum of compound **6j’** (CDCl_3_, 100MHz)


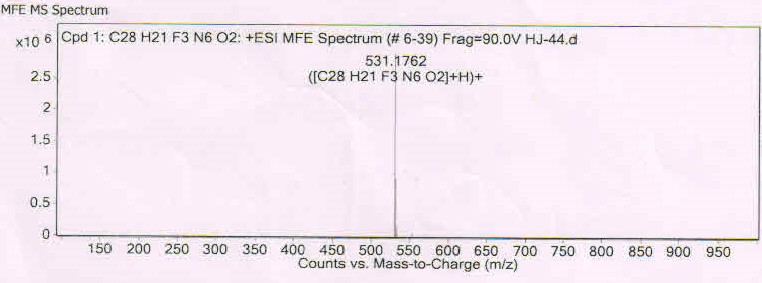


**Figure S106:**HRMS spectrum of compound **6j’**

**Figure S107:**^1^H NMR spectrum of compound **6k’** (CDCl_3_, 400MHz)

**Figure S108:**^13^C NMR spectrum of compound **6k’** (CDCl_3_, 100MHz)


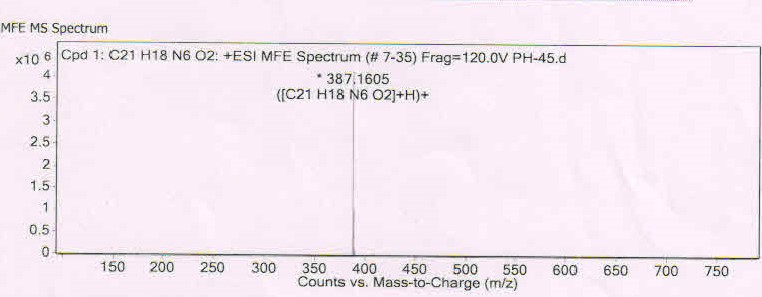


**Figure S109:**HRMS spectrum of compound **6k’**

**Figure S110:**^1^H NMR spectrum of compound **6l’** (CDCl_3_, 400MHz)

**Figure S111:**^13^C NMR spectrum of compound **6l’** (CDCl_3_, 100MHz)


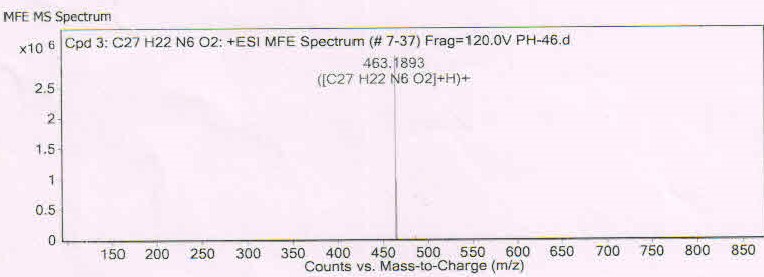


**Figure S112:**HRMS spectrum of compound **6l’**

**Figure S113:**^1^H NMR spectrum of compound **6m’** (CDCl_3_, 400MHz)

**Figure S114:**^13^C NMR spectrum of compound **6m’** (CDCl_3_, 100MHz)


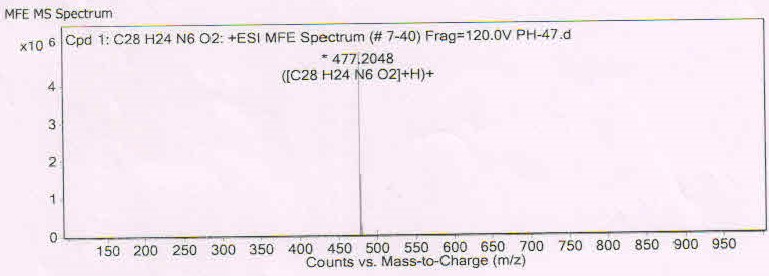


**Figure S115:**HRMS spectrum of compound **6m’**

**Figure S116:**^1^H NMR spectrum of compound **6n’** (CDCl_3_, 400MHz)

**Figure S117:**^13^C NMR spectrum of compound **6n’** (CDCl_3_, 100MHz)


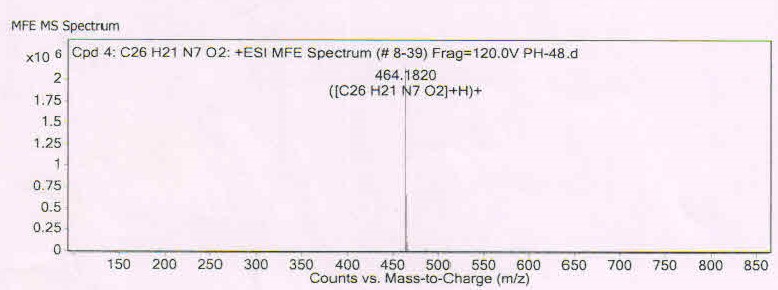


**Figure S118:**HRMS spectrum of compound **6n’**

**Figure S119:** ^1^H NMR spectrum of compound **6o’** (CDCl_3_, 400MHz)

**Figure S120:** ^13^C NMR spectrum of compound **6o’** (CDCl_3_, 100MHz)

**Figure S121:**^1^H NMR spectrum of compound **6p’** (CDCl_3_, 400MHz)

**Figure S122:** ^13^C NMR spectrum of compound **6p’** (CDCl_3_, 100MHz)


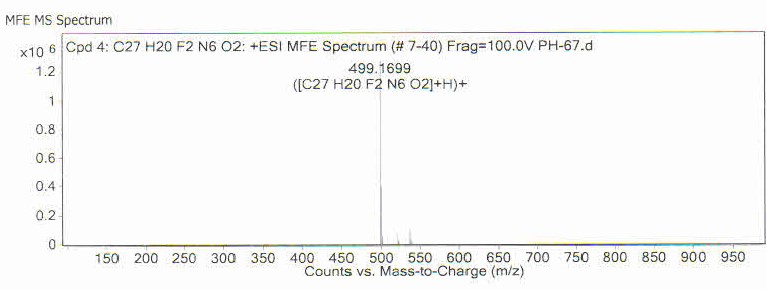


**Figure S123:** HRMS spectrum of compound **6p’**

**Figure S124:** ^1^H NMR spectrum of compound **6q’** (CDCl_3_, 400MHz)

**Figure S125:**^13^C NMR spectrum of compound **6q’** (CDCl_3_, 100MHz)


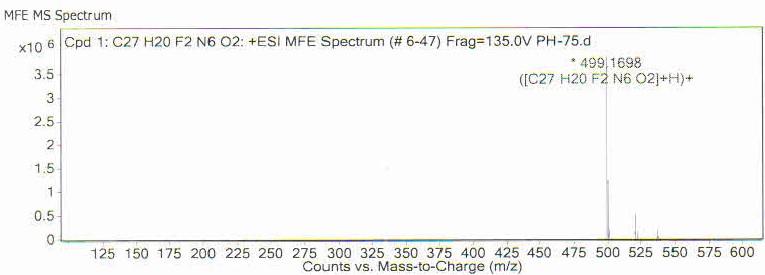


**Figure S126:** HRMS spectrum of compound**6q’**

**Figure S127:** ^1^H NMR spectrum of compound **6r’** (CDCl_3_, 400MHz)

**Figure S128:** ^13^C NMR spectrum of compound **6r’** (CDCl_3_, 100MHz)


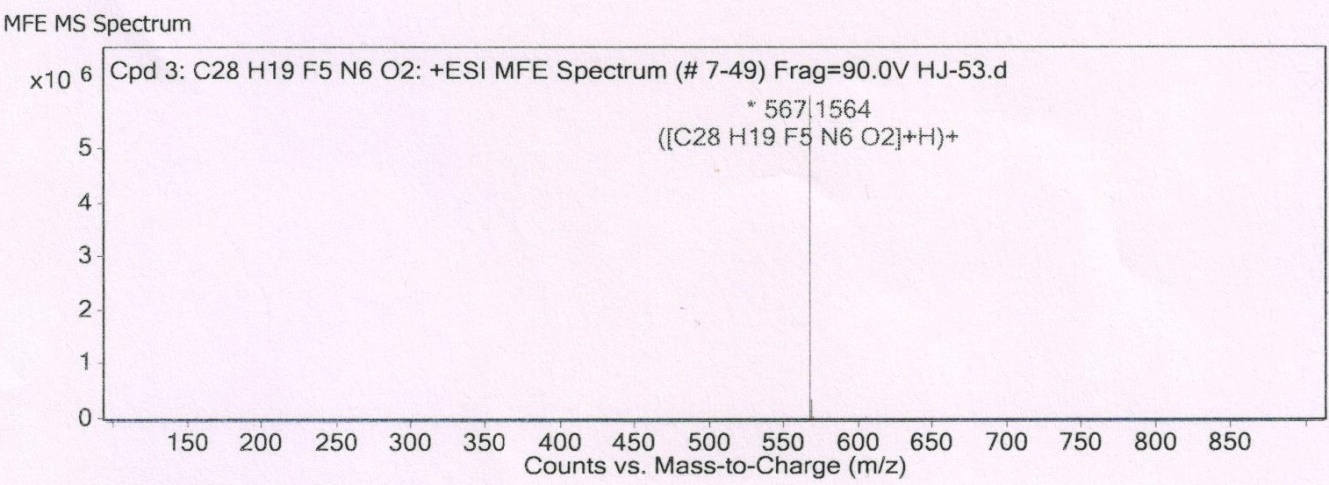


**Figure S129:** HRMS spectrum of compound**6r’**

**Figure S130:** ^1^H NMR spectrum of compound **6s’** (CDCl_3_, 400MHz)

**Figure S131:** ^13^C NMR spectrum of compound **6s’** (CDCl_3_, 100MHz)


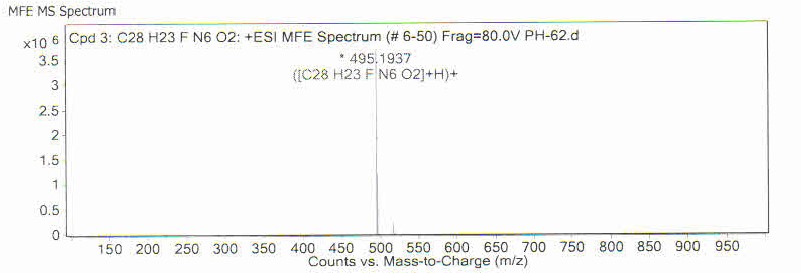


**Figure S132:** HRMS spectrum of compound **6s’**

**Figure S133:** ^1^H NMR spectrum of compound **6t’** (CDCl_3_, 400MHz)

**Figure S134:** ^13^C NMR spectrum of compound **6t’** (CDCl_3_, 100MHz)


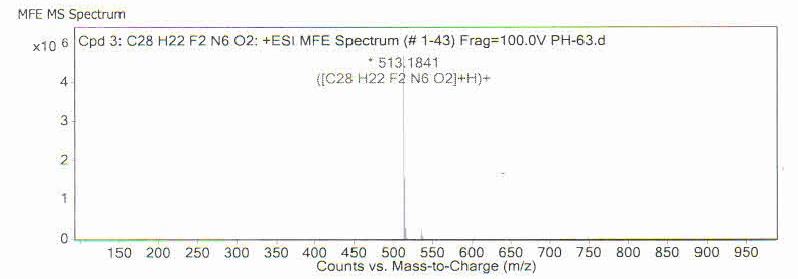


**Figure S135:** HRMS spectrum of compound **6t’**

**Figure S136:** ^1^H NMR spectrum of compound **6u’** (CDCl_3_, 400MHz)

**Figure S137:** ^13^C NMR spectrum of compound **6u’** (CDCl_3_, 100MHz)


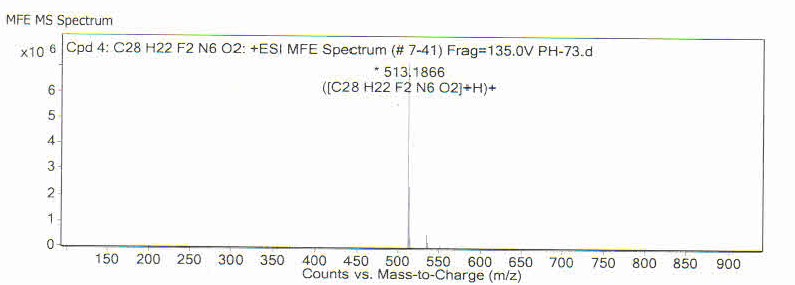


**Figure S138:** HRMS spectrum of compound **6u’**
